# Unified synthesis of mono/bis-arylated phenols *via* Rh^III^-catalyzed dehydrogenative coupling†
†Electronic supplementary information (ESI) available: Experimental procedures and characterization data for all new compounds. CCDC 1047606, 1426755, 1438599 and 1442845. For ESI and crystallographic data in CIF or other electronic format see DOI: 10.1039/c6sc03169b



**DOI:** 10.1039/c6sc03169b

**Published:** 2016-08-03

**Authors:** Qian Wu, Ying Chen, Dingyuan Yan, Muyue Zhang, Yi Lu, Wei-Yin Sun, Jing Zhao

**Affiliations:** a Shenzhen Key Lab of Nano-Micro Material Research , School of Chemical Biology and Biotechnology , Shenzhen Graduate School of Peking University , Shenzhen , 518055 , China . Email: jingzhao@nju.edu.cn; b State Key Laboratory of Coordination Chemistry , Institute of Chemistry and BioMedical Sciences , School of Chemistry and Chemical Engineering , Collaborative Innovation Center of Chemistry for Life Sciences , Nanjing University , Nanjing 210093 , China

## Abstract

2,6-Bis-arylated phenols are rarely reported and are synthetically challenging. Directed C–H functionalization reactions, using a directing group (DG), might provide a convenient solution to their synthesis. However, this strategy usually results in partial cleavage of the directing group, preventing further/second C–H activation cascades. Herein we report a general strategy that allows for the precise control of the oxidation pathways so that directing groups can be either preserved or cleaved. We found that *N*-phenoxyacetamides could undergo *ortho*-arylation reactions with or without an external oxidant, yielding products with different oxidation states, notably the rare bis-arylated phenols. Notably, a unique rhodacycle intermediate was isolated, characterized by X-ray crystallography, and confirmed to be an active catalyst. Switching between internal and external oxidation could be a general strategy in diverse directed C–H functionalization reactions to realize bis-functionalized products.

## Abstract 2

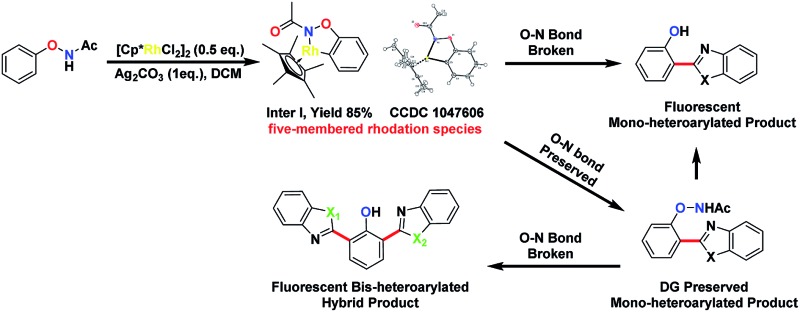
2,6-Bis-arylated phenols are rarely reported and synthetically challenging. We report that switching between internal and external oxidation could be a general strategy to realize these bis-functionalized products.

## Introduction

**Fig. 1:**
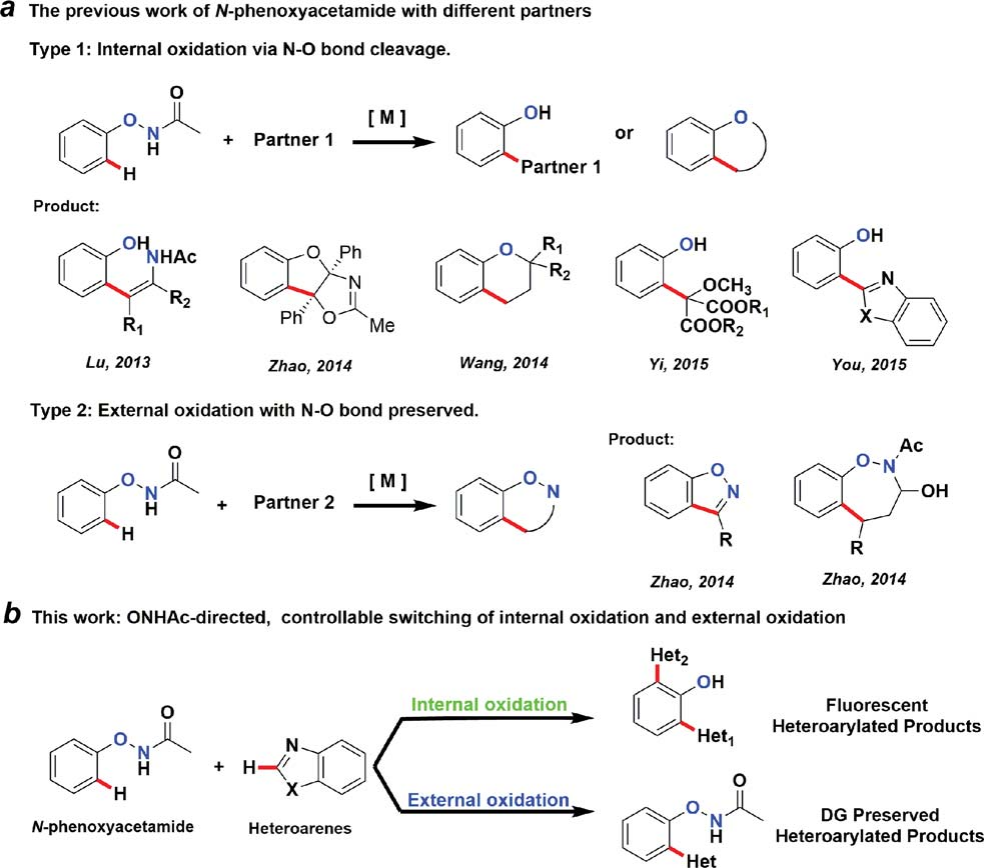
O–NHAc group-directed C–H activation reactions.

Transition metal-catalysed C–H activation reactions directed by a coordinative group have become one of the most efficient and straightforward synthetic strategies for the direct functionalization of inert C–H bonds.^1^ The role of a DG extends beyond a simple anchor for the selective cleavage of a neighbouring C–H bond. DGs can often undergo further *in situ* condensation reactions that yield products of great structural diversity.^2^ More recently, DGs containing an oxidative N–O or N–N bond were introduced to replace the required external oxidant to render the C–H functionalization reactions redox-neutral.^2d,3^ Among them, oxyacetamide (O–NHAc) is one of the most versatile functionalities for directed C–H functionalization reaction cascades (Fig. 1a). The reactions involving this unique DG can be classified into two types:

Type 1: internal oxidation with N–O bond cleavage. Lu’s group first reported O–NHAc as a superb directing group for redox-neutral olefination reactions of phenol derivatives by coupling with alkynes^3*l*^ and alkenes.^3*n*^ Subsequently, our group reported a number of reaction cascades using rhodium catalysis in which complexed heterocyclic scaffolds were synthesized in one step from *N*-phenoxyacetamides and alkynes with up to a quadruple cascade.^2*d*^ Wang and Yi made impressive progress on O–NHAc-directed C–H reactions using cyclopropenes^3*t*^ and carbenoids^3*x*^ as coupling partners, respectively. In these reactions, the N–O bond of the DG was cleaved and serves as an internal oxidant, leading to the corresponding phenol products. Notably, You’s group reported a pioneering C–H/C–H cross-coupling between *N*-phenoxyacetamides and heteroarenes through a traceless directing strategy to synthesize the highly functionalized 2-(2-hydroxyphenyl)azoles, which are novel optoelectronic materials.^3*v*^

Type 2: external oxidation with preservation of the N–O bond. In contrast to type 1 reactions, we recently described a series of oxidative C–H functionalization reactions. In the presence of a stoichiometric external oxidant, *N*-phenoxyacetamides could react with aldehydes or α,β-unsaturated aldehydes using palladium^4^ and rhodium^5^ catalysis. In these reactions, the N–O bond of the DG remained intact after the reaction and was retained in the products.

**Scheme 1:**
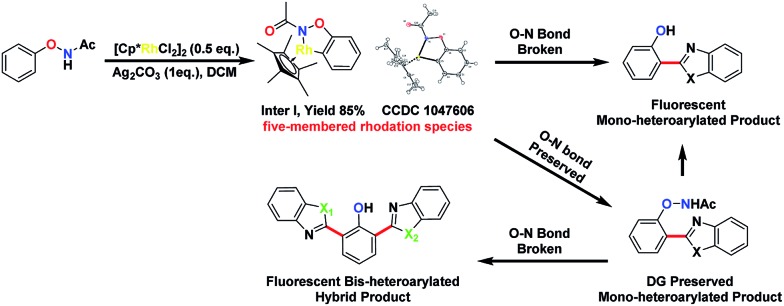
Proposed transformations based on an isolated organometallic intermediate.

We thought that the unification of type 1 and type 2 into a single reaction would allow general access to products with different oxidation states with controlling the cleavage of the N–O bond. For example, based on an isolated rhodacycle intermediate **Inter I** ^6^ from our previous studies,^5^ the O–NHAc-directed cross dehydrogenative coupling reactions with simple heteroarenes would be an attractive strategy to access diverse heteroarylated phenol scaffolds (Scheme 1).^1b,d,7–9^

Heteroarylated phenols, such as 2-(2-hydroxyphenyl)benzothiazole (HBT) and 2-(2-hydroxyphenyl)benzoxazole (HBO), possess high fluorescence quantum yields and a large Stokes shift due to the excited-state intramolecular proton transfer (ESIPT) effect, and are widely used in various fluorescent probes and related fields.^10^ If the redox activity of the N–O bond could be tuned using a proper external oxidant, switching between type 1 and 2 reactions could be enabled, and up to three coupling products could be obtained selectively in a unified fashion (Scheme 1). Based on this design, we set out to explore the combination of different reaction parameters from phenoxyacetamides to achieve a unified strategy.

## Results and discussion

We embarked on our design by investigating a reaction between *N*-phenoxyacetamide (**1a**) and benzothiazole (**2a**). As expected, the desired *ortho*-heteroarylated phenol (**3aa**) was obtained as the main product in the absence of any external oxidant. After careful condition optimization, [Cp*RhCl_2_]_2_ (5 mol%), AgNTf_2_ (25 mol%), and CsOAc (2.5 eq.) in 1 mL DMSO at 85 °C for 30 h under an N_2_ atmosphere was found as the best reaction conditions for **3aa**, affording an 85% isolated yield. A non-coordinating counter ion (SbF_6_, NTf_2_, CO_3_, OTf) was essential for the catalytic activity of Rh.^3d,k,t,11^

A series of substituted *N*-phenoxyacetamides were examined for substrate scope (Table 1). For methyl-substituted *N*-phenoxyacetamides, the *para*-methyl substrate **1b** gave the corresponding phenol product **3ba** in 80% yield. The *meta*-methyl analogue **1c** afforded a 72% yield, and the *ortho*-methyl derivative **1d** yielded 76% of the product. The yield for *meta*-OMe *N*-phenoxyacetamide **1j** was noticeably less. No obvious electronic effect was observed. Substrates bearing either electron-donating (phenyl and methoxy) or electron-withdrawing groups (ester), delivered their corresponding heteroaryl phenols in comparable yields.

**Table 1:** Substrate scope of fluorescent mono-arylated products **3** ^*a*^ ^,^ ^*b*^

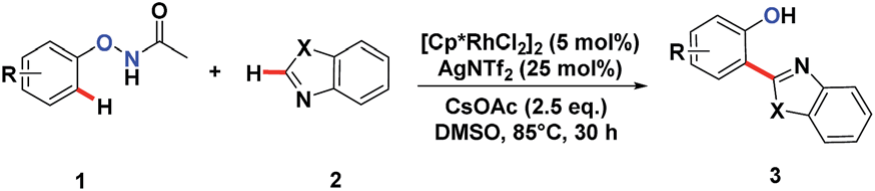
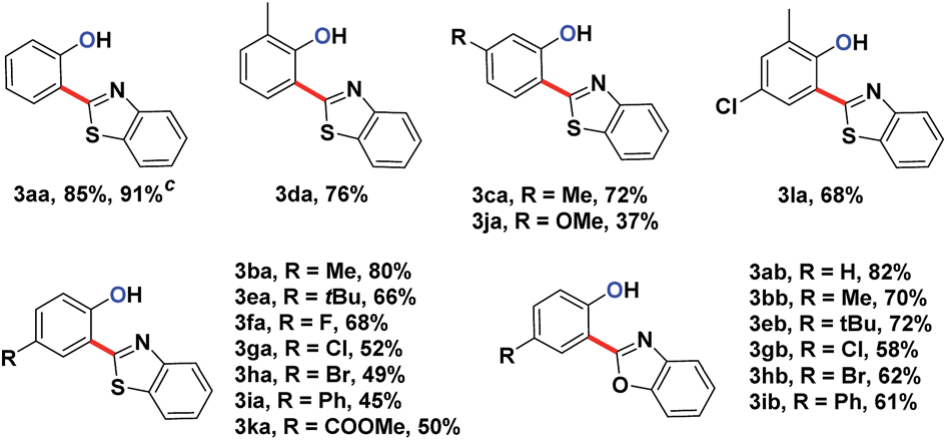

^*a*^Conditions: *N*-phenoxyacetamine (0.2 mmol), heteroarenes (0.3 mmol), [Cp*RhCl_2_]_2_ (5 mol%), AgNTf_2_ (25 mol%), CsOAc (2.5 eq.), DMSO (1 mL) at 85 °C for 30 hours under an N_2_ atmosphere.

^*b*^Isolated yield.

^*c*^GC yield.

In the reaction of the mono-heteroarylated product **3aa**, a different product with a stronger fluorescence was isolated in a small quantity. This double arylation product was determined as the bis-heteroarylated phenol by single crystal X-ray crystallography and it became predominant when a silver salt was introduced as the external oxidant along with 2.5 eq. of benzothiazole. Treating **1a** and benzothiazole with [Cp*RhCl_2_]_2_ (5 mol%) and PivOH (3 eq.) in THF at room temperature for 20 h afforded **4aa** in 78% isolated yield. Highly fluorescent bis-heteroarylated phenols were obtained in moderate to high yields under the optimized reaction conditions (Table 2). *Para*- and *meta*-substituents were well tolerated (**4ba**, **4ea**, **4ka** and **4ja**). In addition, benzoxazoles reacted with comparable efficiency (**4eb**).

**Table 2:** Substrate scope of fluorescent bis-arylated products **4** ^*a*^ ^,^ ^*b*^

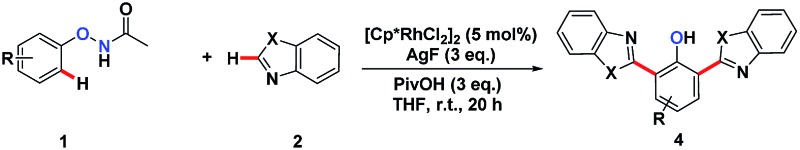
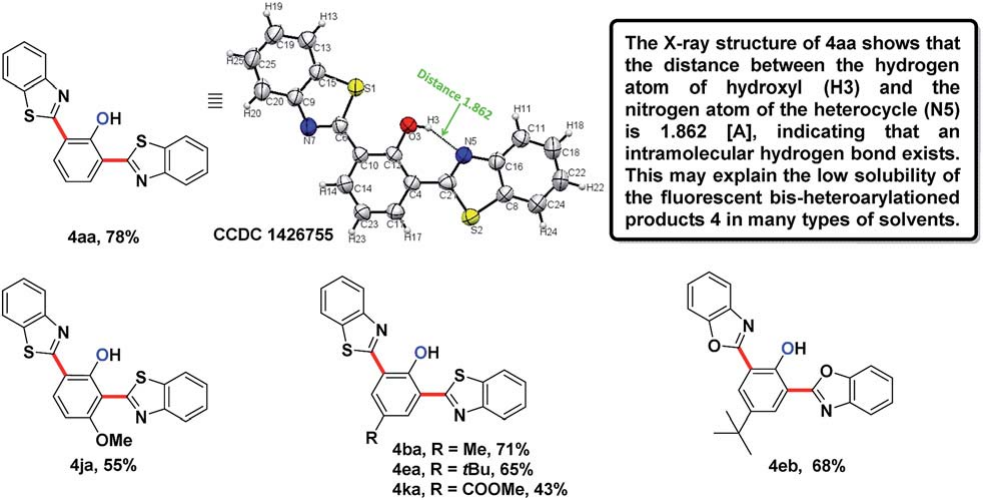

^*a*^Reaction conditions: *N*-phenoxyacetamine (0.2 mmol), heteroarenes (0.5 mmol), [Cp*RhCl_2_]_2_ (5 mol%), AgF (3 eq.), PivOH (3 eq.), THF (1 mL) at room temperature for 20 hours in air.

^*b*^Isolated yield.

**Fig. 2:**
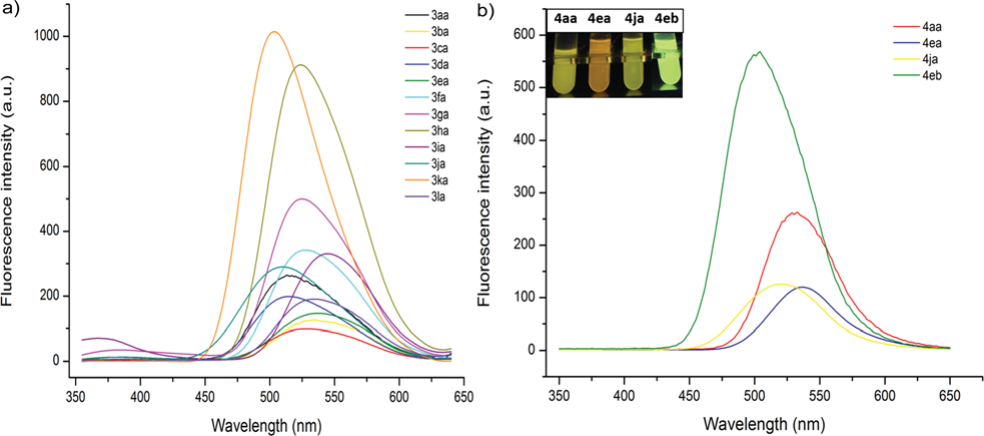
Fluorescence properties of **3** and **4**. (a) Fluorescence spectra of mono-substituted HBTs in DCM (2 × 10^–6^ mol L^–1^, *λ*_ex_ = 330 nm). (b) Fluorescence spectra of bis-substituted products in DCM (2 × 10^–6^ mol L^–1^, *λ*_ex_ = 360 nm).

Interestingly, subjecting product **3aa** and benzothiazole to various Rh catalysis conditions did not yield **4aa**, with or without silver oxidants. This result suggested that **3aa** was not one of the intermediates leading to **4aa**. The formation of the bis-heteroarylated phenol arose from the type 2 product B (Fig. 2). Based on this result, we decided to target this type 2 product. Despite previous reports that the N–O bond was always broken in the presence of a silver oxidant, we were excited to isolate a small amount of the mono-heteroarylated *N*-phenoxyacetamide **5aa** (8% yield) by replacing PivOH with excess triethylamine (10 eq.). The structure was unambiguously confirmed by NMR, HRMS and single crystal X-ray crystallography. Further condition optimization revealed that [Cp*RhCl_2_]_2_ (5 mol%), AgF (3 eq.) and benzothiazole (2 eq.) in a solvent mixture of ^i^PrOH : *N*,*N*-diisopropylethylamine (DIPEA) : H_2_O = 1 : 1 : 0.1 at room temperature for 18 h afforded **5aa** in a 20% yield. The starting material *N*-phenoxyacetamide **1aa** remained largely intact. Adding AgF in small portions (2 eq. upon mixing and 1 eq. every six hours for 4 eq. total) improved the yield of **5aa** to 68%.

The substrate scope for this type 2 heteroarylation reaction was explored using the optimized conditions. *para*-Substituted *N*-aryloxyacetamides afforded the corresponding products in moderate yields (**5ba**, **5ea**, **5ga**, **5ha**, **5ia**, **5ka** in Table 3). The yield for the *para*-COOMe substrate was particularly high (72%), suggesting that an electron-withdrawing group favoured the type 2 reaction. A lower yield was observed for those carrying a meta-substituent. *meta*-Methyl and *meta*-methoxy substrates gave the desired products in 30% and 35% yield, respectively (**5ca**, **5ja**). Both substituted benzothiazoles and benzoxazoles worked equally well (**5ac**, **5kd**).

**Table 3:** Substrate scope of DG-preserved arylated products **5** ^*a*^ ^,^ ^*b*^

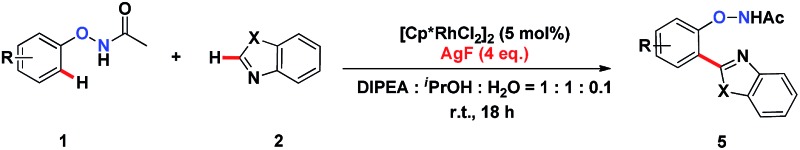
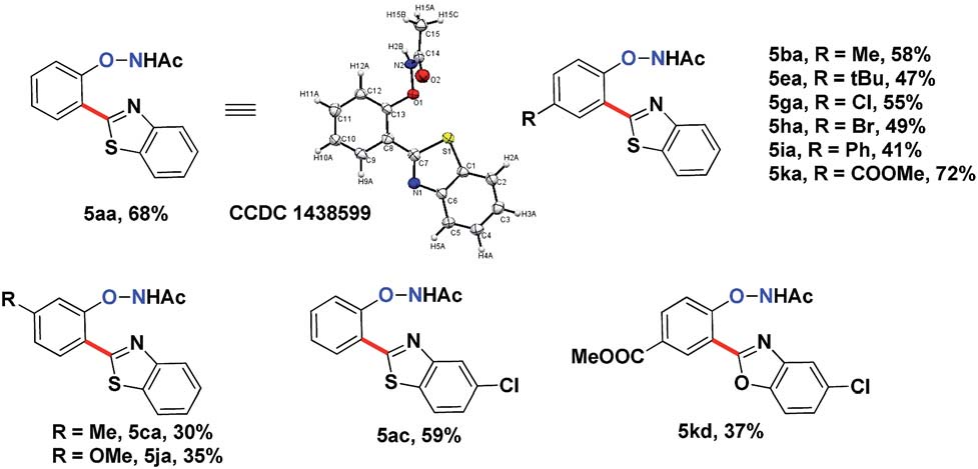

^*a*^Reaction conditions: *N*-phenoxyacetamine (0.2 mmol), heteroarenes (0.4 mmol), [Cp*RhCl_2_]_2_ (5 mol%), AgF (4 eq., 2 eq. added for the first time and 1 eq. every 6 hours twice), DIPEA : ^i^PrOH : H_2_O = 1 : 1 : 0.1 at room temperature for 18 hours under air.

^*b*^Isolated yield.

**Scheme 2:**
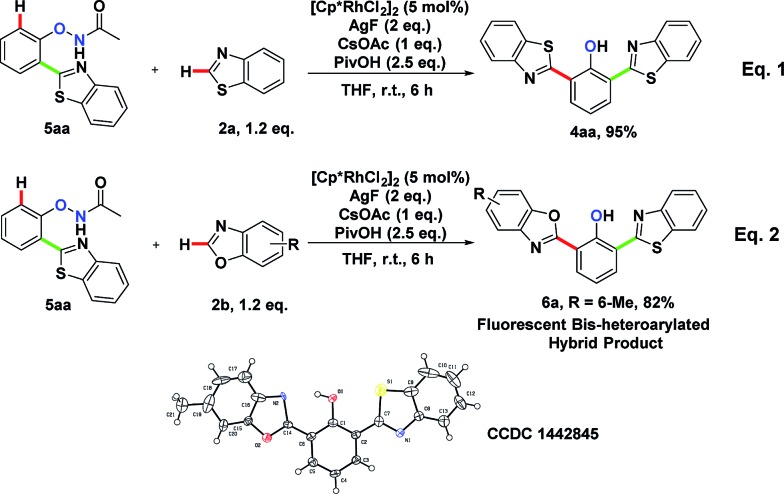
Route to novel fluorescent bis-heteroarylated hybrid products.

To explore the formation of bis-heteroarylated phenols, phenol **5aa** was subjected to the rhodium catalyst in the presence of an external oxidant AgF (2 eq.) and **4aa** was obtained nearly quantitatively (Scheme 2, eqn (1)). Encouraged by this result, we attempted to synthesize more interesting hybrid bis-heteroarylated phenols. Gratifyingly, the hybrid product **6a** was obtained in high yield (Scheme 2, eqn (2)). This type 2–type 1 sequence enhanced the structural diversity of these fluorescent bis-heteroarylated phenols and provided a general strategy for devising better fluorescent probes.

**Scheme 3:**
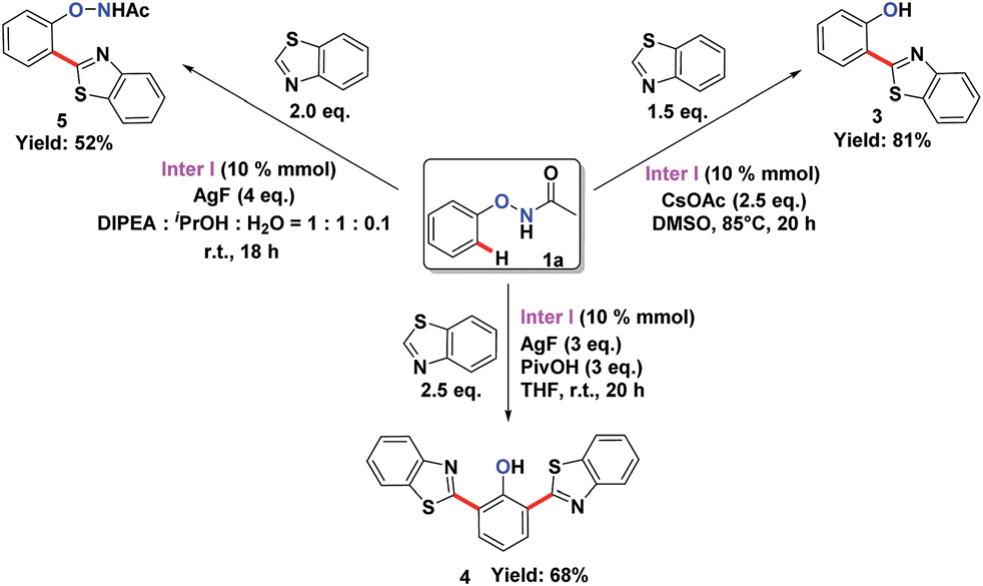
The confirmation of catalytically active species **Inter I**.

To understand the mechanism of the O–NHAc-directed C–H activation reactions, we obtained the five-membered rhodation intermediate **Inter I** in 85% yield. The structure was confirmed by NMR spectroscopy, HRMS, and X-ray crystallography (Fig. S1†). When **Inter I** was used as the catalyst, our three reaction conditions led to three expected heteroarylated products, suggesting the rhodation species **Inter I** was the active intermediate (Scheme 3).

You’s group had demonstrated that the reaction might start from the cyclometalation of *N*-aryloxyacetamide rather than the heteroarene by *ortho*-deuterium labelling experiments. You’s group reported that the KIE value was 1.04 for the *N*-phenoxyacetamide substrate, while the KIE value was 2.89 for the benzoxazole substrate, suggesting that the rate-limiting step might involve the C–H bond breaking of heteroarenes.^3v,12^ Thus, we proposed a mechanism with two pathways of internal oxidation and external oxidation to afford different products (Fig. S1†). First, after the generation of the real [Rh^III^] catalyst by the anion exchange of [NTf_2_]^–^, a five-membered rhodation species **Inter I** formed, which was demonstrated as an active intermediate. Second, the heteroarene was inserted to give a heteroaryl–Rh^III^–phenyl intermediate, which would undergo reductive elimination to a Rh^I^ complex (**Inter III** in Fig. S1†). Third, two pathways are possible depending on the presence of the external oxidants: (1) in the absence of the external oxidants, the Rh^I^ complex **Inter III** would undergo an internally oxidizing pathway to form a Rh^III^ complex (**Inter IV** in Fig. S1†) with O–N bond cleavage.^3l,n^ Protonation would afford the mono-arylated product **3**. (2) In the presence of the external oxidants, the Rh^I^ complex **Inter III** could undergo an external oxidation pathway to form the O–N bond-preserved mono-arylated product **5**. As the DG was retained in product **5**, it could subsequently react with another heteroarene to afford the bis-arylated products **4**.

## Application

The fluorescent properties of mono- and bis-heteroarylated phenols were evaluated (Fig. 2). Considering the solvent effect on ESIPT, a series of common organic solvents was screened, and dichloromethane was chosen for measurement (Fig. S6†).^13^ The fluorescence spectra of mono-substituted HBTs showed a strong ESIPT emission band in the region of 480–540 nm. Both the *λ*_max_ and the intensity of the absorption were affected by the substituents. A methyl group in the *para*- and *meta*-positions caused a bathochromic shift (∼15 nm), while the *ortho*-counterpart showed no obvious change. Halogen substituents (–F, Cl, Br) led to increased fluorescence intensity with small red shifts (∼10 nm). Products with an extra phenyl group resulted in the highest red shift (∼25 nm), and the ester group caused the highest blue shift. By contrast, the bis-substituted products demonstrated significant bathochromic shifts with strong yellow fluorescence.

Considering that the fluorescence of the DG-preserved products **5** was effectively blocked due to the O–N bond, a small molecule that can cleave the O–N bond would have great potential for developing fluorescent probes.

## Conclusions

In summary, we developed a unified strategy for cross dehydrogenative coupling reactions between arenes and heteroarenes. Internal and external oxidation could be controlled using N–O bond cleavage or a silver oxidant. Mono- and the rarely reported bis-arylated phenol derivatives of different oxidation states were prepared in one step. This convenient, one-step synthesis of a series of DG-preserved products could facilitate the continued generation of a library of fluorescent probes. Switching between internal and external oxidation could be a general strategy in other directed C–H functionalization reactions to realize the bis-functionalized products.
