# Supplementary material for: Unified synthesis of mono/bis-arylated phenols via RhIII-catalyzed dehydrogenative coupling
Source: Chem Sci. 2016 Aug 3;8(1):169–73. doi: 10.1039/c6sc03169b (PMC5308286; doi:10.1039/c6sc03169b)
Supplement: Supplementary file 1 [file SC-008-C6SC03169B-s001.pdf]

Electronic Supplementary Material (ESI) for Chemical Science.
This journal is © The Royal Society of Chemistry 2016

Unified Synthesis of Mono/Bis-arylated Phenols via Rh(III)-Catalyzed Dehydrogenative Coupling

Qian Wu, Ying Chen, Dingyuan Yan, Muyue Zhang, Yi Lu, Wei-Yin Sun, and Jing Zhao*

Supporting Information

Table of contents

I. General ……………………………………………………….…………………S2

II. Preparation of substrates……………………………………………………..... S2

III. The fluorescent mono-heteroarylated products

General procedure A……………………………………...………………..S3

Characterization of products 3…………….………………….……….…..…S3

IV. The fluorescent bis-heteroarylated products

General procedure B……………………………….……..………………..S7

Characterization of products 4………………………………..….……….…..…S7

V. External oxidation pathway towards DG preserved mono-heteroarylated products

General procedure C……………………………..……..…………………..S9

Characterization of products 5…………………………………..………….…..…S9

VI. The fluorescent bis-heteroarylated hybrid products

General procedure D……………………………………..………………S11

Characterization of products 6……………………...……..……………….…….S11

VII. Mechanism study……………………………….…...……………….…………S11

VIII. X-ray crystallographic data……….………...………………………......S12

IX. The excitation and emission spectras………............………………………....S35

X. References……………………………………………………………...…………S36

XI. ^{1}H and ^{13}C NMR spectra…………………………...……………………..S37

I. General

All reactions were carried out under an atmosphere of nitrogen unless otherwise noted. Reaction temperatures are reported as those of the oil bath. The dry solvents used were purified by distillation and were transferred under nitrogen.

Commercially available chemicals were obtained from Sigma-Aldrich, Alfa Aesar, TCI and Aladdin and used as received unless otherwise stated. Dichloro (η^5-pentamethylcyclopentadienyl) rhodium(III) dimer (99%) was purchased from Sinocompound Catalysts Co., Ltd.

Reactions were monitored with analytical thin-layer chromatography (TLC) on silica. ^{1}H NMR and ^{13}C NMR data were recorded on Bruker nuclear resonance (400 MHz) spectrometers unless otherwise specified, respectively. Chemical shifts (δ) are given in ppm relative to TMS. The residual solvent signals were used as references and the chemical shifts converted to the TMS scale ($CDCl_3$: δ_H=7.26 ppm, δ_c=77.16 ppm; CD_2Cl_2: δ_H=5.32 ppm, δ_c=53.84 ppm; DMSO: δ_H=2.50 ppm, δ_c=39.52 ppm). HRMS (ESI) analysis was performed by The Analytical Instrumentation Center at University, Shenzhen Graduate School and (HRMS) data were reported with ion mass/charge (m/z) ratios as values in atomic mass units. The visible spectroscopy was detected Shimadzu UV-2600 UV-Vis spectrophotometer and the fluorescence spectrophotometry was detected on Shimadzu RF-5301PC spectrofluorophotometer.

II. Preparation of substrates

Synthesis of 1a-1l

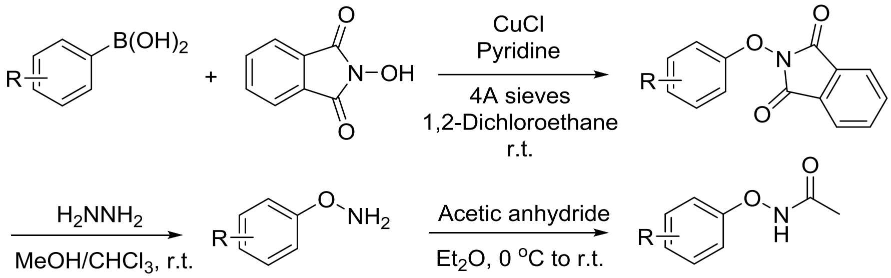

1a - 1l

General procedure:

Following literature reports[1], in a 50mL round-bottom flask, *N*-hydroxy - phthalimide (1.0 eq.), cooper (I) chloride (1.0 eq.), freshly activated 4 Å molecular sieves (250 mg/mmol), and phenylboronic acid (2.0 eq.) were combined in 1,2-dichloroethane (0.2 M). The pyridine (1.1 eq.) was then added to the suspension. The reaction mixture was open to the atmosphere and stirred at room temperature over 24-48 h. Upon completion, silica gel was added to the flask and the solvent was removed under vacuum. The desired *N*-aryloxyphthalimides were obtained by flash column chromatography on silica gel.

Hydrazine monohydrate (3.0 eq.) was added to the solution of *N*-aryloxyphth - alimide (1.0 eq.) in 10% MeOH in $CHCl_3$ (0.1 M). The reaction was allowed to stir at room temperature over 12 h. Upon completion, the reaction mixture was filtered off

and washed with CH_2Cl_2. The filtrate was concentrated under reduced pressure, and purified by flash silica gel column chromatography to give the corresponding *N*-aryloxyamine.

In a 20 mL round-bottom flask, *N*-aryloxyamine (1.0 eq.) was dissolved in ether (0.2 M).The flask was cooled in an ice bath, to which acetic anhydride (2.0 eq.) was slowly added. The ice bath was allowed to warm to room temperature and the mixture was stirred for 3 h at room temperature. The reaction mixture was concentrated under reduced pressure and purified by flash silica gel column chromatography to give the corresponding *N*-phenoxyacetamide.

III. The fluorescent mono-heteroarylated products

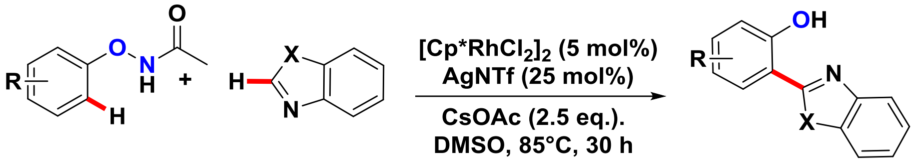

1 2 3

General procedure A:

N-phenoxyacetamide (**1**) (0.2 mmol), $[Cp^*RhCl_2]_2$ (5 mol%), benzothiazole or benzoxazole (**2**) (0.3 mmol), AgNTf (25 mol%), and CsOAc (2.5 eq.) without external oxidant were weighed into a 10 mL pressure tube, to which was added DMSO (1 mL) in a glove box. The reaction vessel was stirred at 85 ℃ for 30h. Then the mixture was concentrated under vacuum and the residue was purified by column chromatography on silica gel with a gradient eluent of petroleum ether and ethyl acetate to afford the corresponding product.

Characterization of products 3:

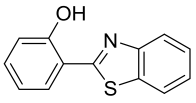

3aa
2-(benzo[*d*]thiazol-2-yl)phenol

The title compound was obtained in 85% yield as white solid; ^1H NMR (500 MHz, $CDCl_3$) δ 12.52 (s, 1H), 7.99 (d, J = 8.2 Hz, 1H), 7.91 (d, J = 7.9 Hz, 1H), 7.70 (dd, J = 7.8, 1.5 Hz, 1H), 7.51 (s, 1H), 7.40 (d, J = 13.1 Hz, 2H), 7.11 (dd, J = 8.3, 0.8 Hz, 1H), 6.96 (s, 1H). ^{13}C NMR (126 MHz, $CDCl_3$) δ 169.54, 158.11, 152.02, 132.91, 132.76, 128.57, 126.84, 125.70, 122.34, 121.66, 119.67, 118.03, 116.96. HRMS (ESI): Calcd. for C13H9NOS (M+H) 228.0483; Found: 228.0478.

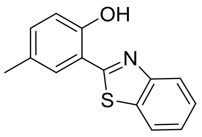

3ba
2-(benzo[*d*]thiazol-2-yl)-4-methylphenol

The title compound was obtained in 80% yield as white solid; ^1H NMR (400 MHz, $CDCl_3$) δ 12.31 (s, 1H), 7.98 (d, J = 8.7 Hz, 1H), 7.90 (d, J = 7.4 Hz, 1H), 7.54 – 7.46 (m, 2H), 7.44 – 7.38 (m, 1H), 7.19 (dd, J = 8.4, 2.1 Hz, 1H), 7.01 (d, J = 8.4 Hz, 1H), 2.36 (s, 3H). ^{13}C NMR (101 MHz, $CDCl_3$) δ 169.53, 155.92, 152.05, 133.86, 132.73, 128.79, 128.44, 126.75, 125.54, 122.24, 121.60, 117.78, 116.47, 20.60. HRMS (ESI): Calcd. for C14H11NOS (M+H) 242.0640; Found: 242.0635.

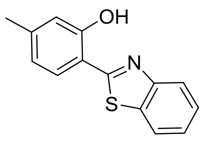

3ca
2-(benzo[*d*]thiazol-2-yl)-5-methylphenol

The title compound was obtained in 72% yield as white solid; ^{1}H NMR (400 MHz, $CDCl_3$) δ 12.45 (s, 1H), 7.96 (d, J = 8.2 Hz, 1H), 7.88 (d, J = 8.0 Hz, 1H), 7.56 (d, J = 8.0 Hz, 1H), 7.51 – 7.46 (m, 1H), 7.38 (ddd, J = 8.3, 7.2, 1.2 Hz, 1H), 6.92 (d, J = 0.8 Hz, 1H), 6.77 (dd, J = 8.0, 1.6 Hz, 1H), 2.37 (s, 3H). ^{13}C NMR (101 MHz, $CDCl_3$) δ 169.56, 157.99, 152.03, 143.93, 132.57, 128.37, 126.72, 125.42, 122.11, 121.59, 120.84, 118.20, 114.49, 21.88. HRMS (ESI): Calcd. for C14H11NOS (M+H) 242.0640; Found: 242.0631.

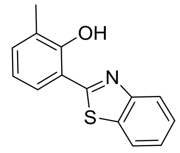

3da
2-(benzo[*d*]thiazol-2-yl)-6-methylphenol

The title compound was obtained in 76% yield as white solid; ^{1}H NMR (400 MHz, $CDCl_3$) δ 12.76 (s, 1H), 7.96 (d, J = 8.1 Hz, 1H), 7.89 (d, J = 7.1 Hz, 1H), 7.55 (d, J = 8.0 Hz, 1H), 7.52 – 7.47 (m, 1H), 7.42 – 7.37 (m, 1H), 7.28 – 7.23 (m, 1H), 6.86 (t, J = 7.6 Hz, 1H), 2.37 (s, 3H). ^{13}C NMR (101 MHz, $CDCl_3$) δ 169.94, 156.41, 151.97, 133.81, 132.84, 127.07, 126.75, 126.19, 125.55, 122.19, 121.60, 119.11, 116.16, 16.18. HRMS (ESI): Calcd. for C14H11NOS (M+H) 242.0640; Found: 242.0635.

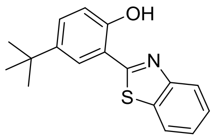

3ea
2-(benzo[*d*]thiazol-2-yl)-4-(*tert*-butyl)phenol

The title compound was obtained in 66% yield as white solid; ^{1}H NMR (400 MHz, $CDCl_3$) δ 12.33 (s, 1H), 7.99 (d, *J* = 9.0 Hz, 1H), 7.91 (d, *J* = 8.1 Hz, 1H), 7.65 (d, *J* = 2.4 Hz, 1H), 7.53 – 7.48 (m, 1H), 7.46 – 7.38 (m, 2H), 7.05 (d, *J* = 8.7 Hz, 1H), 1.37 (s, 9H). ^{13}C NMR (101 MHz, $CDCl_3$) δ 169.94, 155.85, 152.11, 142.44, 132.71, 130.52, 126.74, 125.52, 124.72, 122.26, 121.59, 117.61, 116.09, 34.25, 31.52. HRMS (ESI): Calcd. for C17H17NOS (M+H) 284.1109; Found: 284.1104.

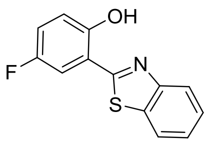

3fa
2-(benzo[*d*]thiazol-2-yl)-4-fluorophenol

The title compound was obtained in 68% yield as white solid; ^{1}H NMR (400 MHz, $CDCl_3$) δ 12.31 (s, 1H), 8.01 (d, J = 7.2 Hz, 1H), 7.92 (d, J = 7.2 Hz, 1H), 7.56 – 7.50 (m, 1H), 7.47 – 7.41 (m, 1H), 7.38 (dd, J = 8.8, 2.8 Hz, 1H), 7.16 – 7.01 (m, 2H). ^{13}C NMR (75 MHz, $CDCl_3$) δ 168.32, 157.36, 154.32, 154.20, 151.92, 132.83, 127.06, 126.04, 122.55, 121.75, 120.15, 119.84, 119.26, 119.16, 116.82, 116.71, 113.97, 113.65. HRMS (ESI): Calcd. for C13H8FNOS (M+H) 246.0389; Found: 246.0385.

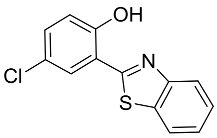

3ga
2-(benzo[*d*]thiazol-2-yl)-4-chlorophenol

The title compound was obtained in 52% yield as white solid; ^{1}H NMR (400 MHz, $CDCl_3$) δ 12.54 (s, 1H), 8.01 (d, J = 8.2 Hz, 1H), 7.93 (d, J = 7.5 Hz, 1H), 7.66 (d, J = 2.5 Hz, 1H), 7.56 – 7.51 (m, 1H), 7.47 – 7.42 (m, 1H), 7.33 (dd, J = 8.9, 2.5 Hz, 1H), 7.06 (d, J = 8.8 Hz, 1H). ^{13}C NMR (126 MHz, $CDCl_3$) δ 168.08, 156.75, 151.88, 132.82, 132.66, 127.68, 127.08, 126.09, 124.31, 122.54, 121.76, 119.58, 117.88. HRMS (ESI): Calcd. for C13H8ClNOS (M+H) 262.0093; Found: 262.0089.

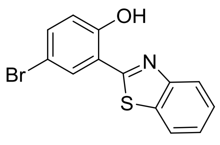

3ha
2-(benzo[*d*]thiazol-2-yl)-4-bromophenol

The title compound was obtained in 49% yield as white solid; ^{1}H NMR (400 MHz, Chloroform-d) δ 12.56 (s, 1H), 8.00 (d, J = 8.2 Hz, 1H), 7.93 (d, J = 8.2 Hz, 1H), 7.79 (d, J = 2.3 Hz, 1H), 7.55 – 7.51 (m, 1H), 7.47 – 7.41 (m, 2H), 7.00 (d, J = 8.8 Hz, 1H). ^{13}C NMR (101 MHz, $CDCl_3$) δ 167.79, 157.02, 151.64, 135.33, 132.62, 130.49, 126.95, 125.97, 122.39, 121.65, 119.83, 118.35, 111.03. HRMS (ESI): Calcd. for C13H8BrNOS (M+H) 305.9588; Found: 305.9580.

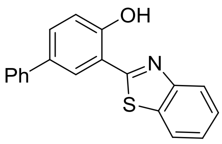

3ia
3-(benzo[*d*]thiazol-2-yl)-[1,1'-biphenyl]-4-ol

The title compound was obtained in 45% yield as white solid; ^{1}H NMR (400 MHz, Chloroform-d) δ 12.55 (s, 1H), 7.98 (d, J = 8.1 Hz, 1H), 7.89 (d, J = 7.9 Hz, 1H), 7.85 (d, J = 2.2 Hz, 1H), 7.62 – 7.54 (m, 3H), 7.52 – 7.30 (m, 5H), 7.15 (d, J = 8.6 Hz, 1H). ^{13}C NMR (101 MHz, $CDCl_3$) δ 169.44, 157.59, 152.00, 140.26, 133.07, 132.77, 131.81, 129.05, 127.25, 126.91, 126.90, 125.78, 122.38, 121.71, 118.48, 117.11, 117.08. HRMS (ESI): Calcd. for C19H13NOS (M+H) 304.0796; Found: 304.0794.

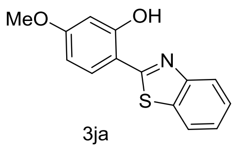

3ja
2-(benzo[*d*]thiazol-2-yl)-5-methoxyphenol

The title compound was obtained in 37% yield as white solid; ^{1}H NMR (400 MHz, $CDCl_3$) δ 12.74 (s, 1H), 7.93 (d, J = 7.8 Hz, 1H), 7.87 (d, J = 7.4 Hz, 1H), 7.58 (d, J = 8.7 Hz, 1H), 7.51 – 7.45 (m, 1H), 7.40 – 7.34 (m, 1H), 6.60 (d, J = 2.5 Hz, 1H), 6.54 (dd, J = 8.7, 2.5 Hz, 1H), 3.86 (s, 3H). ^{13}C NMR (101 MHz, $CDCl_3$) δ 169.42, 163.58, 160.09, 151.99, 132.30, 129.76, 126.69, 125.16, 121.81, 121.55, 110.54, 107.83, 101.47, 55.64. HRMS (ESI): Calcd. for C14H11NO2S (M+H) 258.0589; Found: 258.0584.

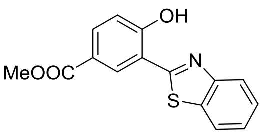

3ka
methyl 3-(benzo[*d*]thiazol-2-yl)-4-hydroxybenzoate

The title compound was obtained in 50% yield as white solid; ^{1}H NMR (400 MHz, $CDCl_3$) δ 13.13 (s, 1H), 8.43 (d, J = 2.0 Hz, 1H), 8.05 (dd, J = 8.7, 2.1 Hz, 1H), 8.01 (d, J = 8.1 Hz, 1H), 7.96 – 7.92 (m, 1H), 7.53 (ddd, J = 8.3, 7.3, 1.3 Hz, 1H), 7.45 (ddd, J = 8.3, 7.2, 1.2 Hz, 1H), 7.13 (d, J = 8.7 Hz, 1H), 3.94 (s, 3H). ^{13}C NMR (101 MHz, $CDCl_3$) δ 168.78, 166.31, 161.86, 151.61, 133.95, 132.78, 130.73, 127.04, 126.06, 122.40, 121.79, 121.77, 118.10, 116.67, 52.27. HRMS (ESI): Calcd. for C15H11NO3S (M+H) 286.0538; Found: 286.0544.

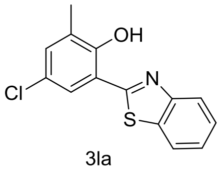

3la
2-(benzo[*d*]thiazol-2-yl)-4-chloro-6-methylphenol

The title compound was obtained in 68% yield as white solid; ^{1}H NMR (400 MHz, $CDCl_3$) δ 12.76 (s, 1H), 7.97 (d, J = 8.0 Hz, 1H), 7.91 (d, J = 8.1 Hz, 1H), 7.54 – 7.48 (m, 2H), 7.45 – 7.40 (m, 1H), 7.21 (d, J = 2.3 Hz, 1H), 2.33 (s, 3H). ^{13}C NMR (101 MHz, $CDCl_3$) δ 168.48, 155.05, 151.74, 133.28, 132.81, 129.18, 126.95, 125.90, 125.09, 123.55, 122.34, 121.68, 116.88, 16.14. HRMS (ESI): Calcd. for C14H10ClNOS (M+H) 276.0250; Found: 276.0237.

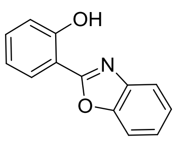

3ab
2-(benzo[*d*]oxazol-2-yl)phenol

The title compound was obtained in 82% yield as white solid; ^{1}H NMR (500 MHz, $CDCl_3$) δ 11.48 (s, 1H), 8.02 (dd, J = 7.9, 1.7 Hz, 1H), 7.76 – 7.70 (m, 1H), 7.63 – 7.58 (m, 1H), 7.47 – 7.42 (m, 1H), 7.41 – 7.35 (m, 2H), 7.13 (d, J = 8.2 Hz, 1H), 7.05 – 6.98 (m, 1H). ^{13}C NMR (126 MHz, $CDCl_3$) δ 163.02, 158.87, 149.26, 140.15, 133.68, 127.24, 125.49, 125.12, 119.68, 119.37, 117.54, 110.77. HRMS (ESI): Calcd. for C13H9NO2 (M+H) 212.0712; Found: 212.0720.

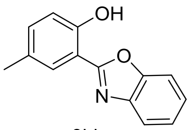

3bb
2-(benzo[*d*]oxazol-2-yl)-
4-methylphenol

The title compound was obtained in 70% yield as white solid; ^{1}H NMR (500 MHz, $CDCl_3$) δ 11.27 (s, 1H), 7.81 (d, J = 2.3 Hz, 1H), 7.74 – 7.69 (m, 1H), 7.62 – 7.57 (m, 1H), 7.39 – 7.35 (m, 2H), 7.24 (dd, J = 8.4, 2.3 Hz, 1H), 7.02 (d, J = 8.5 Hz, 1H), 2.37 (s, 3H). ^{13}C NMR (126 MHz, $CDCl_3$) δ 162.99, 156.65, 149.11, 140.11, 134.50, 128.73, 126.91, 125.25, 124.92, 119.19, 117.18, 110.56, 110.10, 20.47. HRMS (ESI): Calcd. for C14H11NO2 (M+H) 226.0868; Found: 226.0863.

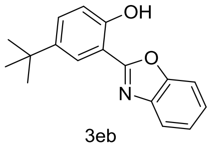

3eb
2-(benzo[*d*]oxazol-2-yl)-
4-(*tert*-butyl)phenol

The title compound was obtained in 72% yield as white solid; ^{1}H NMR (500 MHz, $CDCl_3$) δ 11.34 (s, 1H), 8.03 (d, J = 2.5 Hz, 1H), 7.74 – 7.70 (m, 1H), 7.65 – 7.60 (m, 1H), 7.50 (dd, J = 8.7, 2.5 Hz, 1H), 7.37 (dd, J = 6.0, 3.2 Hz, 2H), 7.08 (d, J = 8.7 Hz, 1H), 1.40 (s, 9H). ^{13}C NMR (126 MHz, $CDCl_3$) δ 163.37, 156.75, 149.23, 142.44, 140.26, 131.24, 125.33, 125.03, 123.44, 119.29, 117.15, 110.71, 109.88, 34.36, 31.58. HRMS (ESI): Calcd. for C17H17NO2 (M+H) 268.1338; Found: 268.1332.

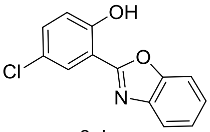

3gb
2-(benzo[*d*]oxazol-2-yl)-
4-chlorophenol

The title compound was obtained in 58% yield as white solid; ^{1}H NMR (400 MHz, $CDCl_3$) δ 11.45 (s, 1H), 8.01 (d, J = 2.6 Hz, 1H), 7.77 – 7.73 (m, 1H), 7.65 – 7.60 (m, 1H), 7.43 – 7.37 (m, 3H), 7.07 (d, J = 8.9 Hz, 1H). ^{13}C NMR (101 MHz, $CDCl_3$) δ 161.84, 157.37, 149.30, 139.94, 133.54, 126.55, 125.99, 125.41, 124.59, 119.60, 119.10, 111.72, 110.95. HRMS (ESI): Calcd. for C13H9NO2Cl (M+H) 246.0322; Found: 246.0317.

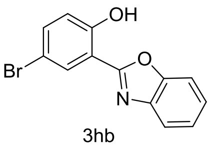

3hb
2-(benzo[*d*]oxazol-2-yl)-
4-bromophenol

The title compound was obtained in 62% yield as white solid; ^{1}H NMR (500 MHz, $CDCl_3$) δ 11.44 (s, 1H), 8.11 (d, J = 2.5 Hz, 1H), 7.75 – 7.69 (m, 1H), 7.63 – 7.57 (m, 1H), 7.49 (dd, J = 8.8, 2.4 Hz, 1H), 7.42 – 7.37 (m, 2H), 7.00 (d, J = 9.0 Hz, 1H). ^{13}C NMR (126 MHz, $CDCl_3$) δ 161.67, 157.80, 149.24, 139.88, 136.29, 129.45, 125.96, 125.37, 119.55, 119.46, 112.25, 111.43, 110.90. HRMS (ESI): Calcd. for C13H8BrNO2 (M+H) 289.9817; Found: 289.9814.

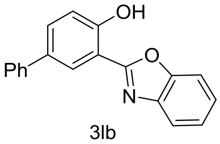

3lb
3-(benzo[*d*]oxazol-2-yl)-
[1,1'-biphenyl]-4-ol

The title compound was obtained in 61% yield as white solid; ^{1}H NMR (500 MHz, $CDCl_3$) δ 11.52 (s, 1H), 8.26 (d, J = 2.3 Hz, 1H), 7.77 – 7.72 (m, 1H), 7.69 (dd, J = 8.6, 2.4 Hz, 1H), 7.65 – 7.61 (m, 3H), 7.47 (t, J = 7.7 Hz, 2H), 7.42 – 7.35 (m, 3H), 7.20 (d, J = 8.6 Hz, 1H). ^{13}C NMR (126 MHz, $CDCl_3$) δ 162.96, 158.33, 149.29, 140.15, 132.98, 132.45, 129.00,

127.22, 126.83, 125.60, 125.49, 125.19, 119.43, 118.01, 110.88, 110.81. HRMS (ESI): Calcd. for C19H13NO2 (M+H) 288.1025; Found: 288.1026.

IV. The fluorescent mono-heteroarylated products

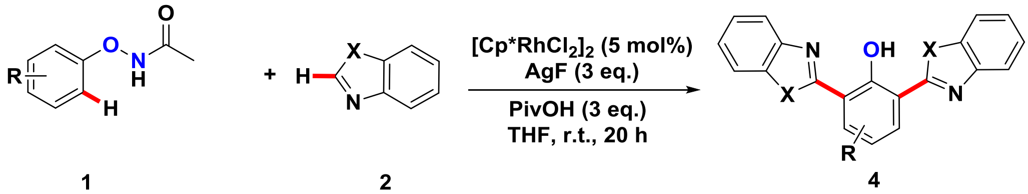

General procedure B:

N-phenoxyacetamide (**1**) (0.2 mmol), $[Cp^*RhCl_2]_2$ (5 mol%), benzothiazole or benzoxazole (**2**) (0.5 mmol), AgF (3 eq.), and PivOH (3 eq.) were weighed into a 10 mL pressure tube, to which was added THF (1 mL) in a glove box. The reaction vessel was stirred at room temperature for 20h. Then the mixture was concentrated under vacuum and the residue was purified by column chromatography on silica gel with a gradient eluent of petroleum ether and ethyl acetate to afford the corresponding product.

Characterization of products 4:

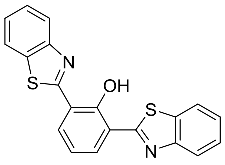

4aa
2,6-bis(benzo[*d*]thiazol-2-yl)phenol

The title compound was obtained in 78% yield as yellow solid; ^{1}H NMR (400 MHz, CD_2Cl_2) δ 8.32 (d, J = 7.6 Hz, 2H), 8.10 (d, J = 8.1 Hz, 2H), 8.00 (d, J = 7.9 Hz, 2H), 7.60 – 7.53 (m, 2H), 7.49 – 7.43 (m, 2H), 7.18 (t, J = 7.8 Hz, 1H). ^{13}C NMR (101 MHz, CD_2Cl_2) δ 156.30, 151.56, 147.02, 131.67, 126.71, 125.55, 122.44, 121.71, 119.83. HRMS (ESI): Calcd. for C20H12N2OS2 (M+H) 361.0469; Found: 361.0463.

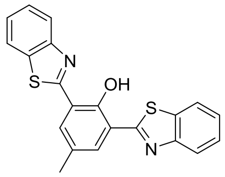

4ba
2,6-bis(benzo[*d*]thiazol-2-yl)-4-methylphenol

The title compound was obtained in 71% yield as yellow solid; ^{1}H NMR (400 MHz, CD_2Cl_2) δ 8.19 (s, 2H), 8.12 (d, J = 8.2 Hz, 2H), 8.00 (d, J = 8.0 Hz, 2H), 7.56 (t, J = 7.3 Hz, 2H), 7.46 (t, J = 7.6 Hz, 2H), 2.50 (s, 3H). ^{13}C NMR (101 MHz, CD_2Cl_2) δ 150.96, 134.16, 132.21, 129.39, 126.70, 125.52, 122.10, 121.62, 20.23. HRMS (ESI): Calcd. for C21H14N2OS2 (M+H) 375.0626; Found: 375.0620.

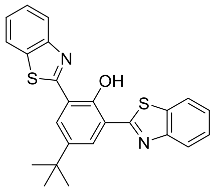

4ea
2,6-bis(benzo[*d*]thiazol-2-yl)-4-(*tert*-butyl)phenol

The title compound was obtained in 65% yield as yellow solid; ^{1}H NMR (500 MHz, CD_2Cl_2) δ 8.32 (s, 2H), 8.10 (d, J = 7.8 Hz, 2H), 8.00 (d, J = 7.9 Hz, 2H), 7.58 – 7.52 (m, 2H), 7.47 – 7.42 (m, 2H), 1.48 (s, 9H). ^{13}C NMR (126 MHz, CD_2Cl_2) δ 166.85, 154.69, 152.30, 143.24, 135.07, 129.22, 127.08, 125.88, 122.94, 122.14, 119.86, 34.99, 31.75. HRMS (ESI): Calcd. for C24H20N2OS2 (M+H) 417.1095; Found: 417.1091.

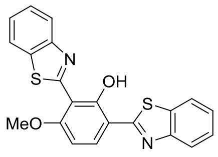

4ja
2,6-bis(benzo[*d*]thiazol-2-yl)-3-methoxyphenol

The title compound was obtained in 55% yield as yellow solid; ^{1}H NMR (400 MHz, CD_2Cl_2) δ 8.72 (d, J = 9.0 Hz, 1H), 8.07 (dd, J = 8.2, 3.4 Hz, 2H), 8.02 – 7.94 (m, 2H), 7.57 (ddd, J = 8.2, 7.1, 1.2 Hz, 1H), 7.53 – 7.44 (m, 2H), 7.41 – 7.36 (m, 1H), 6.79 (d, J = 9.0 Hz, 1H), 4.16 (s, 3H). ^{13}C NMR (101 MHz, CD_2Cl_2) δ 165.71, 163.84, 160.79, 159.61, 151.75, 148.97, 136.04, 135.27, 133.86, 133.46, 127.25, 126.60, 125.89, 124.94, 122.42, 121.94, 121.79, 115.36, 107.53, 102.73, 56.76. HRMS (ESI): Calcd. for C21H14N2O2S2 (M+H) 391.0575; Found: 391.0570.

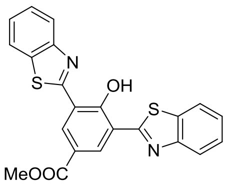

4ka
methyl 3,5-bis(benzo[*d*]thiazol-2-yl)-4-hydroxybenzoate

The title compound was obtained in 43% yield as yellow solid; ^{1}H NMR (500 MHz, Methylene Chloride-d2) δ 8.92 (s, 1H), 8.12 (d, J = 7.9 Hz, 1H), 8.02 (d, J = 7.8 Hz, 1H), 7.58 (t, J = 7.4 Hz, 1H), 7.48 (t, J = 7.4 Hz, 1H), 3.99 (s, 2H). ^{13}C NMR (126 MHz, CD_2Cl_2) δ 166.25, 160.00, 152.13, 135.19, 133.08, 127.37, 126.35, 123.22, 122.81, 122.30, 120.65, 52.83, 30.32. HRMS (ESI): Calcd. for C22H14N2O3S2 (M+1) 419.0524; Found: 419.0519.

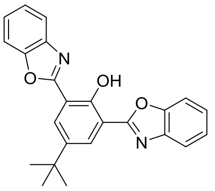

4eb
2,6-bis(benzo[*d*]oxazol-2-yl)-4-(*tert*-butyl)phenol

The title compound was obtained in 68% yield as yellow solid; ^{1}H NMR (500 MHz, CD_2Cl_2) δ 8.34 (s, 2H), 7.83 – 7.79 (m, 2H), 7.71 – 7.67 (m, 2H), 7.45 – 7.40 (m, 4H), 1.47 (s, 9H). ^{13}C NMR (126 MHz, CD_2Cl_2) δ 162.81, 156.34, 150.64, 143.18, 141.63, 130.37, 126.08, 125.47, 120.31, 114.20, 111.30, 35.02, 31.75. HRMS (ESI): Calcd. for C24H20N2O3 (M+H) 385.1552; Found: 385.1547.

V. External oxidation pathway towards DG preserved mono-hetero arylated products

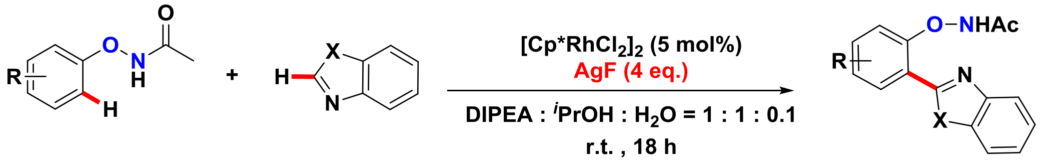

1 2 5

General procedure B:

N-phenoxyacetamide (**1**) (0.2 mmol), $[Cp^*RhCl_2]_2$ (5 mol%), benzothiazole or benzoxazole (**2**) (0.4 mmol), AgF (4 equiv., 2 eq. added for the first time and 1 eq. every 6 hours for 2 times) in ^{i}PrOH: ethyldiisopropylamine (DIPEA): H_2O= 1: 1: 0.1, at room temperature for 18 hours. Then the mixture was concentrated under vacuum and the residue was purified by column chromatography on silica gel with a gradient eluent of dichloromethane and ethyl acetate to afford the corresponding product.

Characterization of products 5:

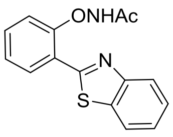

5aa
N-(2-(benzo[*d*]thiazol-2-yl) phenoxy)acetamide

The title compound was obtained in 68% yield as white solid; ^{1}H NMR NMR (400 MHz, DMSO-d^{6}) δ 12.15 (s, 1H), 8.40 (d, J = 8.0 Hz, 1H), 8.15 (d, J = 8.0 Hz, 1H), 8.09 (d, J = 8.1 Hz, 1H), 7.60 – 7.50 (m, 2H), 7.48 – 7.43 (m, 1H), 7.35 (d, J = 8.5 Hz, 1H), 7.25 (t, J = 7.5 Hz, 1H), 2.00 (s, 3H). ^{13}C NMR (101 MHz, DMSO) δ 167.63, 157.20, 151.62, 135.59, 132.28, 128.95, 126.37, 125.15, 122.88, 122.62, 121.96, 119.52, 113.21, 19.47. HRMS (ESI): Calcd. for $C_{15}H_{12}N_2O_2NaS$ (M+H) 285.0698; Found: 285.0695.

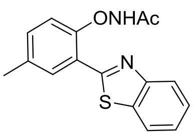

5ba
N-(2-(benzo[*d*]thiazol-2-yl)-4-methylphenoxy)acetamide

The title compound was obtained in 58% yield as white solid; ^{1}H NMR NMR (400 MHz, DMSO-d^{6}) δ 12.09 (s, 1H), 8.21 (d, J = 2.2 Hz, 1H), 8.15 (d, J = 7.5 Hz, 1H), 8.08 (d, J = 7.9 Hz, 1H), 7.58 – 7.52 (m, 1H), 7.48 – 7.42 (m, 1H), 7.35 (d, J = 11.0 Hz, 1H), 7.23 (d, J = 8.6 Hz, 1H), 2.38 (s, 3H), 1.99 (s, 3H). ^{13}C NMR (101 MHz, DMSO) δ 167.51, 161.68, 155.34, 151.62, 135.61, 132.76, 131.89, 128.83, 126.34, 125.09, 122.55, 121.93, 119.25, 113.39, 20.04, 19.45. HRMS (ESI): Calcd. for $C_{16}H_{14}N_2O_2S$ (M+H) 299.0854; Found: 299.0847.

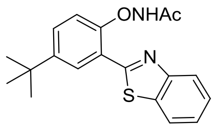

5ea
N-(2-(benzo[*d*]thiazol-2-yl)-4-(*tert*-butyl)phenoxy)acetamide

The title compound was obtained in 47% yield as white solid; ^{1}H NMR NMR (400 MHz, DMSO-d^{6}) δ 12.12 (s, 1H), 8.38 (s, 1H), 8.18 – 8.07 (m, 2H), 7.56 (dd, J = 21.0, 8.4 Hz, 2H), 7.49 – 7.42 (m, 1H), 7.26 (d, J = 8.8 Hz, 1H), 1.98 (s, 3H), 1.34 (s, 9H). ^{13}C NMR (101 MHz, DMSO) δ 167.99, 162.48, 155.76, 152.08, 145.59, 136.09, 129.97, 126.79, 125.55, 123.12, 122.38, 119.31, 113.66, 34.61, 31.65, 19.94. HRMS (ESI): Calcd. for $C_{19}H_{20}N_2O_2S$ (M+H) 341.1324; Found: 341.1320.

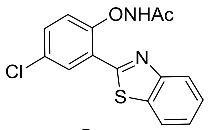

5ga
N-(2-(benzo[*d*]thiazol-2-yl)-4-chlorophenoxy)acetamide

The title compound was obtained in 55% yield as white solid; ^{1}H NMR NMR (300 MHz, DMSO-d^{6}) δ 12.24 (s, 1H), 8.35 (d, J = 2.6 Hz, 1H), 8.16 (d, J = 7.7 Hz, 1H), 8.09 (d, J = 8.1 Hz, 1H), 7.61 – 7.52 (m, 2H), 7.49 – 7.43 (m, 1H), 7.38 (d, J = 9.0 Hz, 1H), 1.98 (s, 3H). ^{13}C NMR (75 MHz, DMSO) δ 168.32, 160.51, 156.46, 151.87, 136.21, 132.15, 128.21, 127.23, 127.08, 125.99, 123.33, 122.58, 121.44, 116.02, 19.90. HRMS (ESI): Calcd. For $C_{15}H_{11}ClN_2O_2S$ (M+H) 319.0308; Found: 319.0304.

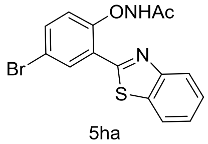

5ha
N-(2-(benzo[*d*]thiazol-2-yl)-4-bromophenoxy)acetamide

The title compound was obtained in 49% yield as white solid; ^{1}H NMR NMR (500 MHz, DMSO-d^{6}) δ 12.29 (s, 1H), 8.50 (d, J = 2.5 Hz, 1H), 8.17 (d, J = 7.9 Hz, 1H), 8.12 (d, J = 7.9 Hz, 1H), 7.71 (dd, J = 8.9, 2.6 Hz, 1H), 7.58 (t, J = 7.7 Hz, 1H), 7.48 (t, J = 7.5 Hz, 1H), 7.35 (d, J = 8.9 Hz, 1H), 2.00 (s, 3H). ^{13}C NMR (126 MHz, DMSO) δ 160.47, 156.97, 151.89, 136.23, 134.92, 131.07, 126.99, 125.91, 123.29, 122.47, 121.88, 116.46, 114.72, 19.76. HRMS (ESI): Calcd. for $C_{15}H_{11}BrN_2O_2S$ (M+H) 362.9803; Found: 362.9792.

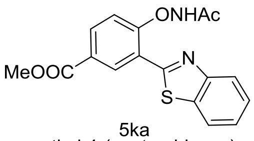

5ka
methyl 4-(acetamidooxy)-
3-(benzo[*d*]thiazol-2-yl)benzoate

The title compound was obtained in 72% yield as white solid; ^{1}H NMR (500 MHz, DMSO-d6) δ 12.35 (s, 1H), 9.04 (d, J = 2.4 Hz, 1H), 8.21 – 8.10 (m, 3H), 7.54 (dt, J = 46.4, 7.8 Hz, 3H), 3.92 (s, 3H), 2.05 (s, 3H). ^{13}C NMR (126 MHz, DMSO) δ 165.17, 160.41, 160.25, 151.45, 135.55, 132.89, 130.22, 126.45, 125.34, 124.22, 122.76, 121.92, 119.46, 113.50, 52.14, 19.35. HRMS (ESI): Calcd. for C17H14N2O4S (M+H) 343.0753; Found: 343.736.

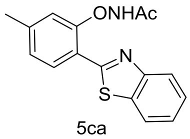

5ca
N-(2-(benzo[*d*]thiazol-2-yl)-
5-methylphenoxy)acetamide

The title compound was obtained in 30% yield as white solid; ^{1}H NMR (400 MHz, DMSO-d6) δ 12.14 (s, 1H), 8.26 (d, J = 8.0 Hz, 1H), 8.10 (d, J = 7.9 Hz, 1H), 8.03 (d, J = 8.1 Hz, 1H), 7.52 (d, J = 7.2 Hz, 1H), 7.41 (t, J = 7.0 Hz, 1H), 7.14 (s, 1H), 7.03 (d, J = 8.0 Hz, 1H), 2.36 (s, 3H), 1.99 (s, 3H). ^{13}C NMR (101 MHz, DMSO) δ 167.99, 162.26, 157.65, 152.12, 143.27, 135.94, 129.26, 126.75, 125.40, 124.14, 122.91, 122.35, 117.49, 113.94, 21.78, 19.96. HRMS (ESI): Calcd. for C16H14N2O2S (M+H) 299.0854; Found: 299.0856.

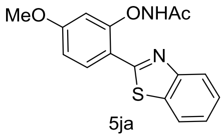

5ja
N-(2-(benzo[*d*]thiazol-2-yl)-
5-methoxyphenoxy)acetamide

The title compound was obtained in 35% yield as white solid; ^{1}H NMR (400 MHz, DMSO-d6) δ 12.11 (s, 1H), 8.35 (d, J = 8.7 Hz, 1H), 8.11 (d, J = 7.9 Hz, 1H), 8.02 (d, J = 8.1 Hz, 1H), 7.56 – 7.49 (m, 1H), 7.45 – 7.38 (m, 1H), 6.85 (d, J = 9.2 Hz, 2H), 3.85 (s, 3H), 2.01 (s, 3H). ^{13}C NMR (101 MHz, DMSO) δ 168.26, 163.15, 162.16, 159.11, 152.16, 135.60, 130.92, 126.69, 125.15, 122.66, 122.29, 113.10, 109.02, 99.39, 56.21, 19.97. HRMS (ESI): Calcd. for C16H14N2O3S (M+H) 315.0803; Found: 315.0797.

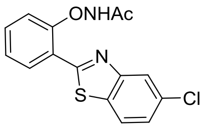

5ac
N-(2-(5-chlorobenzo[*d*]
thiazol-2-yl)phenoxy)acetamide

The title compound was obtained in 59% yield as white solid; ^{1}H NMR (500 MHz, DMSO-d6) δ 12.18 (s, 1H), 8.39 (d, J = 7.9 Hz, 1H), 8.20 (d, J = 8.5 Hz, 1H), 8.16 (d, J = 2.0 Hz, 1H), 7.57 (t, J = 7.8 Hz, 1H), 7.51 (dd, J = 8.5, 2.0 Hz, 1H), 7.36 (d, J = 8.3 Hz, 1H), 7.26 (t, J = 7.5 Hz, 1H), 2.00 (s, 3H). ^{13}C NMR (126 MHz, DMSO) δ 157.28, 152.52, 134.31, 132.60, 131.06, 129.31, 128.91, 125.17, 123.42, 122.86, 121.88, 119.19, 113.22, 112.80, 19.35. HRMS (ESI): Calcd. for C15H11ClN2O2S (M+H) 319.0308; Found: 319.0302.

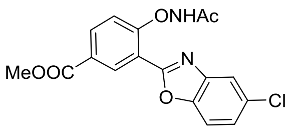

5kd
methyl 4-(acetamidooxy)-3-
(5-chlorobenzo[*d*]oxazol-2-yl)benzoate

The title compound was obtained in 37% yield as white solid; ^{1}H NMR (400 MHz, DMSO-d6) δ 12.16 (s, 1H), 8.65 (d, J = 2.2 Hz, 1H), 8.12 (d, J = 9.0 Hz, 1H), 7.94 (d, J = 2.1 Hz, 1H), 7.84 (d, J = 8.7 Hz, 1H), 7.46 (dd, J = 8.7, 2.2 Hz, 2H), 3.85 (s, 3H), 1.96 (s, 3H). ^{13}C NMR (101 MHz, DMSO) δ 168.19, 164.98, 161.61, 160.81, 148.89, 142.47, 134.44, 132.09, 129.21, 125.94, 124.01, 119.76, 114.00, 112.79, 112.47, 52.40, 19.51. HRMS (ESI): Calcd. for C17H13ClN2O5 (M+H) 361.0591; Found: 361.0587.

VI. The fluorescent bis-heteroarylated hybrid products

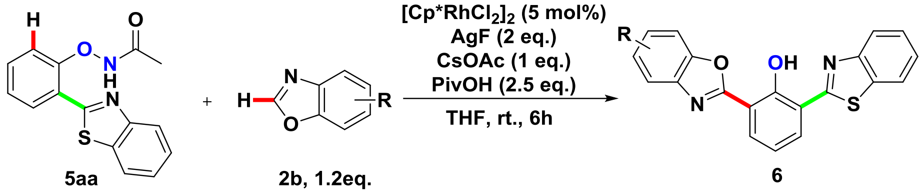

5aa 2b, 1.2eq. 6

Fluorescent Bis-heteroarylationed Hybrid Product

General procedure D:

DG preserved mono-hetero arylated products, *N*-(2-(benzo[*d*]thiazol-2-yl) -phenoxy)acetamide (**5aa)** (0.20 mmol), $[Cp^*RhCl_2]_2$ (5 mol%), benzoxazole (**2b**) (0.24 mmol), AgF (2 eq.), PivOH (2.5 eq.) and CsOAc (1 eq.) were weighed into a 5 mL pressure tube, to which was added THF (1 mL). The reaction vessel was stirred at room temperature for 6 h. Then the mixture was concentrated under vacuum and the residue was purified by column chromatography on silica gel with a gradient eluent of dichloromethane and ethyl acetate to afford the corresponding product.

Characterization of products 6:

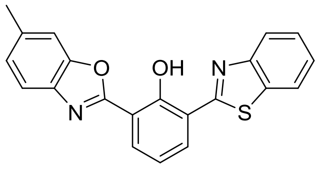

6a
2-(benzo[d]thiazol-2-yl)-6-(6-methylbenzo[d]oxazol-2-yl)phenol

The title compound was obtained in 82% yield as white solid; ^{1}H NMR (500 MHz, CD_2Cl_2) δ 8.93 (d, J = 7.9 Hz, 1H), 8.28 (dd, J = 16.4, 7.9 Hz, 2H), 8.04 (d, J = 8.0 Hz, 1H), 7.68 (d, J = 8.1 Hz, 1H), 7.61 (t, J = 7.6 Hz, 1H), 7.54 – 7.48 (m, 2H), 7.29 (t, J = 8.2 Hz, 2H), 2.55 (s, 3H). ^{13}C NMR (126 MHz, CD_2Cl_2) δ 179.85, 172.00, 162.67, 157.18, 150.23, 141.83, 137.35, 133.37, 130.25, 127.11, 126.76, 125.54, 123.35, 122.10, 120.38, 119.30, 111.56, 111.49, 22.18. HRMS (ESI): Calcd. for C21H14N2O2S (M+H) 359.0854; Found: 359.0846.

VII. Mechanism study

(1) The synthesis of the five-membered rhodation species

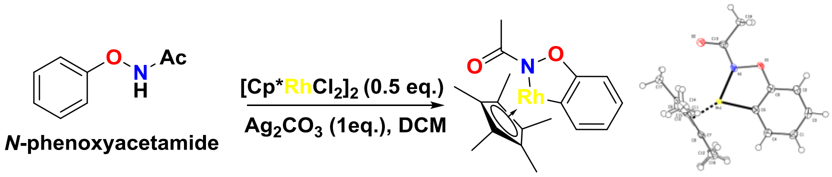

7, Yield 85% CCDC 1047606

five-membered rhodation species

Characterization of rhodation species:

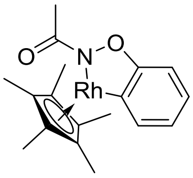

The rhodation species was obtained in 85% yield as red solid; ^{1}H NMR (400 MHz, Chloroform-d) δ 8.35 (d, J = 7.7 Hz, 1H), 7.26 (d, J = 7.9 Hz, 1H), 7.19 (t, J = 6.8 Hz, 1H), 6.97 (t, J = 8.0 Hz, 1H), 2.54 (s, 3H), 1.85 (s, 15H). ^{13}C NMR (101 MHz, CDCl3) δ 178.05, 166.48, 158.22, 157.76, 135.29, 127.65, 118.18, 110.02, 97.89, 97.82, 21.99, 10.52. HRMS (ESI): Calcd. for C18H22NO2Rh (M+H) 388.0784; Found: 388.0783.

(2) Plausible catalytic cycle

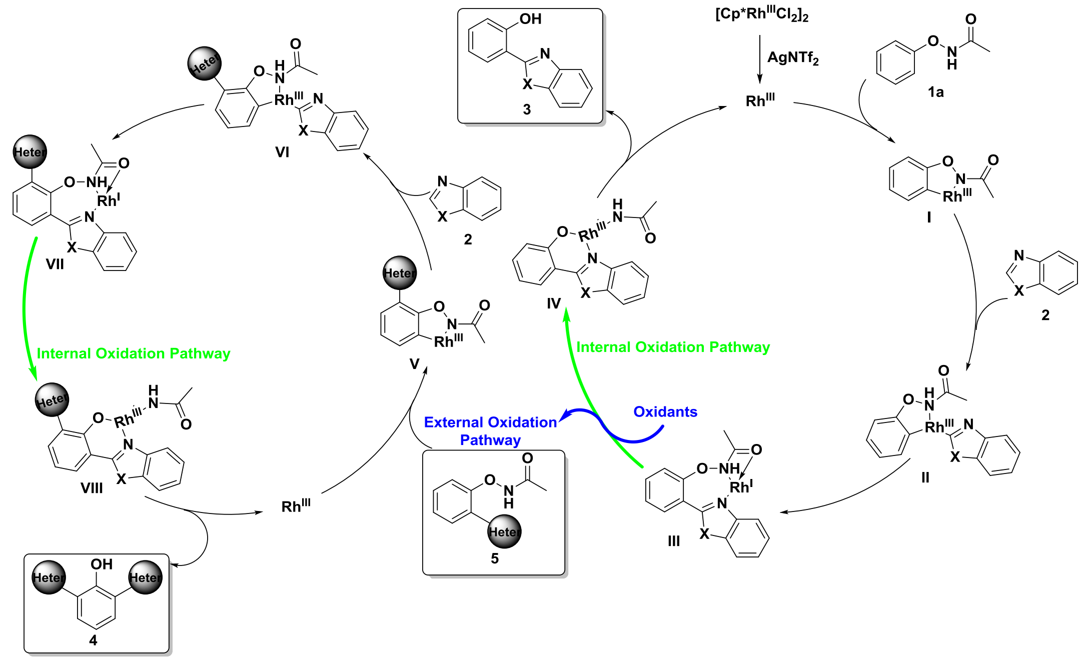

Figure S1. Proposed mechanism

VIII. X-ray Crystallographic Data

Figure S2 Molecular structure and atom numbering scheme for **4aa**.

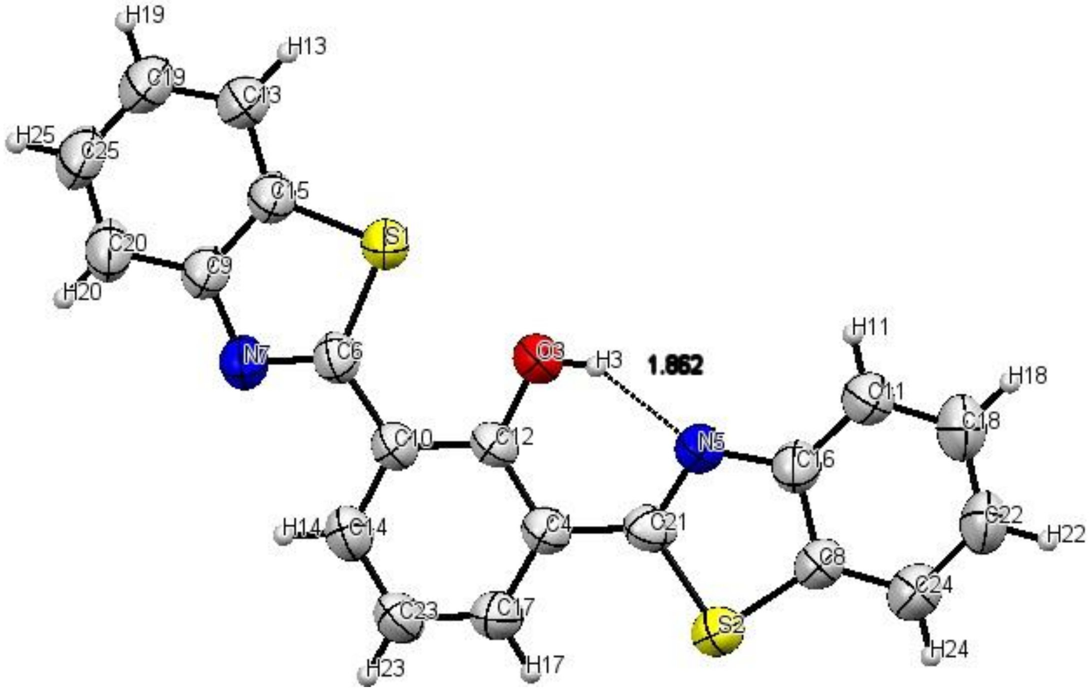

Table S1.1 Crystal data and structure refinement for **4aa**.

Identification code	shelx	
Empirical formula	C20 H12 N2 O S2	
Formula weight	360.44	
Temperature	293(2) K	
Wavelength	1.54187 Å	
Crystal system	Orthorhombic	
Space group	P b c a	
Unit cell dimensions	a = 7.38990(10) Å	α= 90 °.
	b = 13.5531(2) Å	β= 90 °.
	c = 32.123(2) Å	γ = 90 °.
Volume	3217.3(2) Å^3	
Z	8	
Density (calculated)	1.488 Mg/m^3	
Absorption coefficient	3.082 mm^{-1}	
F(000)	1488	
Crystal size	0.200 x 0.080 x 0.010 mm^3	
Theta range for data collection	6.532 to 68.345 °.	
Index ranges	-8<=h<=8, -16<=k<=15, -36<=l<=37	
Reflections collected	19105	
Independent reflections	2903 [R(int) = 0.1139]	
Completeness to theta = 67.687 °	99.2 %	
Absorption correction	Semi-empirical from equivalents	
Max. and min. transmission	0.540 and 0.309	
Refinement method	Full-matrix least-squares on F^2	
Data / restraints / parameters	2903 / 0 / 226	
Goodness-of-fit on F^2	1.095	
Final R indices [I>2sigma(I)]	R1 = 0.0684, wR2 = 0.1715	
R indices (all data)	R1 = 0.1153, wR2 = 0.2246	
Extinction coefficient	n/a	
Largest diff. peak and hole	0.296 and -0.439 e.Å^{-3}	

Table S1.2 Bond lengths [Å] and angles [°] for **4aa**.

S(1)-C(15)	1.727(5)
S(1)-C(6)	1.739(5)
S(2)-C(8)	1.716(5)
S(2)-C(21)	1.739(5)
O(3)-C(12)	1.348(5)
O(3)-H(3)	0.8200
N(5)-C(21)	1.318(6)
N(5)-C(16)	1.387(6)
N(7)-C(6)	1.308(6)
N(7)-C(9)	1.378(6)
C(4)-C(17)	1.379(6)
C(4)-C(12)	1.408(6)
C(4)-C(21)	1.463(6)
C(6)-C(10)	1.483(6)
C(8)-C(24)	1.396(7)
C(8)-C(16)	1.409(6)
C(9)-C(20)	1.396(6)
C(9)-C(15)	1.401(7)
C(10)-C(14)	1.387(6)
C(10)-C(12)	1.404(6)
C(11)-C(18)	1.379(6)
C(11)-C(16)	1.390(6)
C(11)-H(11)	0.9300
C(13)-C(19)	1.384(6)
C(13)-C(15)	1.401(6)
C(13)-H(13)	0.9300
C(14)-C(23)	1.379(6)
C(14)-H(14)	0.9300
C(17)-C(23)	1.374(7)
C(17)-H(17)	0.9300
C(18)-C(22)	1.376(8)
C(18)-H(18)	0.9300
C(19)-C(25)	1.385(7)
C(19)-H(19)	0.9300
C(20)-C(25)	1.367(7)
C(20)-H(20)	0.9300

C(22)-C(24)	1.377(7)
C(22)-H(22)	0.9300
C(23)-H(23)	0.9300
C(24)-H(24)	0.9300
C(25)-H(25)	0.9300
C(15)-S(1)-C(6)	89.1(2)
C(8)-S(2)-C(21)	89.9(2)
C(12)-O(3)-H(3)	109.5
C(21)-N(5)-C(16)	111.3(4)
C(6)-N(7)-C(9)	110.3(4)
C(17)-C(4)-C(12)	119.0(4)
C(17)-C(4)-C(21)	121.3(4)
C(12)-C(4)-C(21)	119.7(4)
N(7)-C(6)-C(10)	120.7(4)
N(7)-C(6)-S(1)	115.8(4)
C(10)-C(6)-S(1)	123.5(3)
C(24)-C(8)-C(16)	120.0(5)
C(24)-C(8)-S(2)	129.9(4)
C(16)-C(8)-S(2)	110.0(3)
N(7)-C(9)-C(20)	125.8(5)
N(7)-C(9)-C(15)	115.4(4)
C(20)-C(9)-C(15)	118.8(5)
C(14)-C(10)-C(12)	118.5(4)
C(14)-C(10)-C(6)	118.9(4)
C(12)-C(10)-C(6)	122.6(4)
C(18)-C(11)-C(16)	118.8(5)
C(18)-C(11)-H(11)	120.6
C(16)-C(11)-H(11)	120.6
O(3)-C(12)-C(10)	117.7(4)
O(3)-C(12)-C(4)	122.3(4)
C(10)-C(12)-C(4)	120.0(4)
C(19)-C(13)-C(15)	116.8(5)
C(19)-C(13)-H(13)	121.6
C(15)-C(13)-H(13)	121.6
C(23)-C(14)-C(10)	121.6(5)
C(23)-C(14)-H(14)	119.2
C(10)-C(14)-H(14)	119.2
C(9)-C(15)-C(13)	122.0(5)

C(9)-C(15)-S(1)	109.4(4)
C(13)-C(15)-S(1)	128.6(4)
N(5)-C(16)-C(11)	125.7(5)
N(5)-C(16)-C(8)	114.1(4)
C(11)-C(16)-C(8)	120.2(5)
C(23)-C(17)-C(4)	121.6(5)
C(23)-C(17)-H(17)	119.2
C(4)-C(17)-H(17)	119.2
C(22)-C(18)-C(11)	120.9(5)
C(22)-C(18)-H(18)	119.6
C(11)-C(18)-H(18)	119.6
C(13)-C(19)-C(25)	121.8(5)
C(13)-C(19)-H(19)	119.1
C(25)-C(19)-H(19)	119.1
C(25)-C(20)-C(9)	119.6(5)
C(25)-C(20)-H(20)	120.2
C(9)-C(20)-H(20)	120.2
N(5)-C(21)-C(4)	123.1(4)
N(5)-C(21)-S(2)	114.8(3)
C(4)-C(21)-S(2)	122.1(4)
C(18)-C(22)-C(24)	121.7(5)
C(18)-C(22)-H(22)	119.1
C(24)-C(22)-H(22)	119.1
C(17)-C(23)-C(14)	119.3(5)
C(17)-C(23)-H(23)	120.4
C(14)-C(23)-H(23)	120.4
C(22)-C(24)-C(8)	118.3(5)
C(22)-C(24)-H(24)	120.8
C(8)-C(24)-H(24)	120.8
C(20)-C(25)-C(19)	120.9(5)
C(20)-C(25)-H(25)	119.6
C(19)-C(25)-H(25)	119.6

Symmetry transformations used to generate equivalent atoms:

Figure S3 Molecular structure and atom numbering scheme for **5aa**

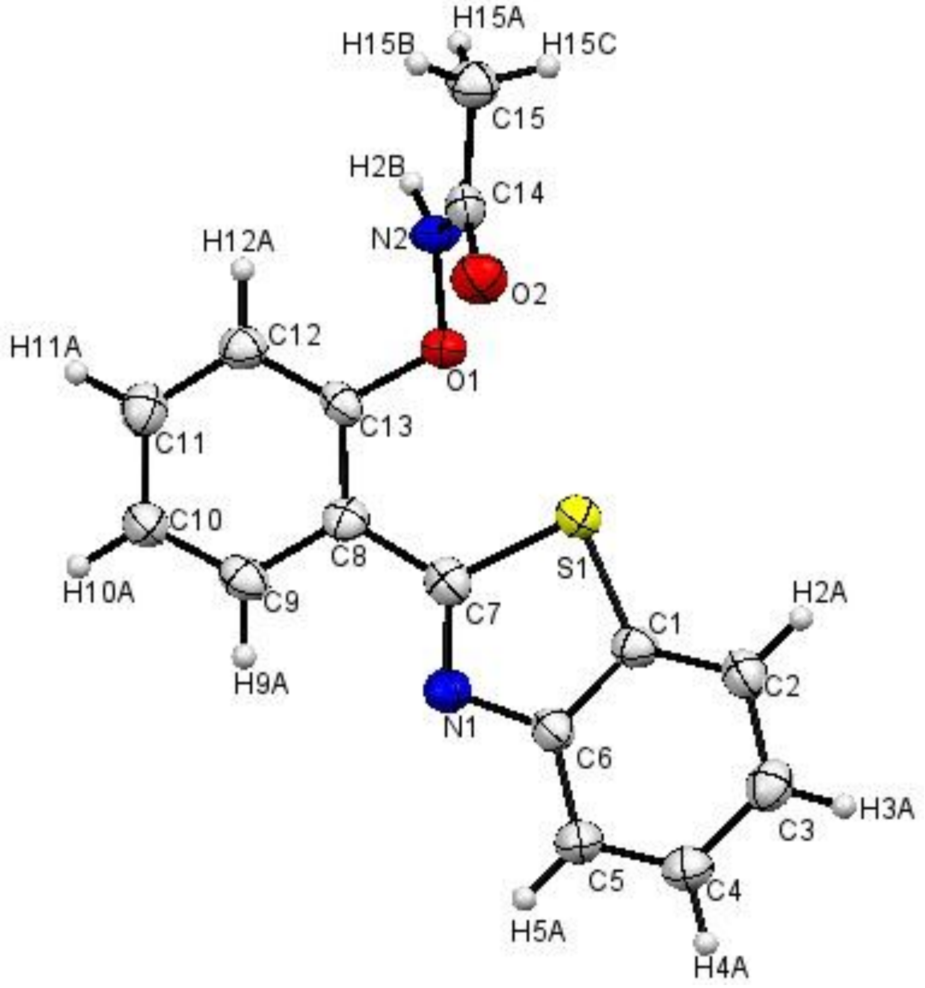

Table S2.1 Crystal data and structure refinement for **5aa**

Identification code	A	
Empirical formula	C3.53 H2.82 N0.47 O0.47 S0.24	
Formula weight	66.90	
Temperature	150(2) K	
Wavelength	0.71073 Å	
Crystal system	Orthorhombic	
Space group	$P2_12_12_1$	
Unit cell dimensions	a = 4.7941(7) Å	α= 90 °.
	b = 15.333(2) Å	β= 90 °.
	c = 17.698(3) Å	γ = 90 °.
Volume	1301.0(3) $Å^3$	
Z	17	
Density (calculated)	1.452 Mg/m^3	
Absorption coefficient	0.251 mm^{-1}	
F(000)	592	
Crystal size	0.186 x 0.086 x 0.053 mm^3	
Theta range for data collection	3.516 to 27.482 °.	
Index ranges	-6<=h<=5, -17<=k<=19, -22<=l<=22	
Reflections collected	7897	
Independent reflections	2850 [R(int) = 0.0835]	
Completeness to theta = 25.242 °	99.4 %	
Absorption correction	Semi-empirical from equivalents	
Max. and min. transmission	0.987 and 0.974	
Refinement method	Full-matrix least-squares on F^2	
Data / restraints / parameters	2850 / 0 / 181	
Goodness-of-fit on F^2	1.042	
Final R indices [I>2sigma(I)]	R1 = 0.0567, wR2 = 0.1193	
R indices (all data)	R1 = 0.0895, wR2 = 0.1374	
Absolute structure parameter	0.13(13)	
Extinction coefficient	n/a	
Largest diff. peak and hole	0.272 and -0.426 e.$Å^{-3}$	

Table S2.2 Bond lengths [Å] and angles [°] for **5aa.**

S(1)-C(1)	1.734(5)
S(1)-C(7)	1.764(5)
N(1)-C(7)	1.310(6)
N(1)-C(6)	1.397(6)
N(2)-C(14)	1.356(6)
N(2)-O(1)	1.413(5)
N(2)-H(2B)	0.8800
O(1)-C(13)	1.394(6)
O(2)-C(14)	1.233(6)
C(1)-C(6)	1.402(7)
C(1)-C(2)	1.403(7)
C(2)-C(3)	1.388(7)
C(2)-H(2A)	0.9500
C(3)-C(4)	1.403(8)
C(3)-H(3A)	0.9500
C(4)-C(5)	1.384(7)
C(4)-H(4A)	0.9500
C(5)-C(6)	1.402(7)
C(5)-H(5A)	0.9500
C(7)-C(8)	1.481(7)
C(8)-C(13)	1.402(7)
C(8)-C(9)	1.406(7)
C(9)-C(10)	1.379(7)
C(9)-H(9A)	0.9500
C(10)-C(11)	1.395(7)
C(10)-H(10A)	0.9500
C(11)-C(12)	1.395(8)
C(11)-H(11A)	0.9500
C(12)-C(13)	1.388(7)
C(12)-H(12A)	0.9500
C(14)-C(15)	1.503(7)
C(15)-H(15A)	0.9800
C(15)-H(15B)	0.9800
C(15)-H(15C)	0.9800
C(1)-S(1)-C(7)	89.0(2)
C(7)-N(1)-C(6)	110.9(4)

C(14)-N(2)-O(1)	116.5(4)
C(14)-N(2)-H(2B)	121.8
O(1)-N(2)-H(2B)	121.8
C(13)-O(1)-N(2)	113.9(3)
C(6)-C(1)-C(2)	121.1(5)
C(6)-C(1)-S(1)	110.1(4)
C(2)-C(1)-S(1)	128.8(4)
C(3)-C(2)-C(1)	118.0(5)
C(3)-C(2)-H(2A)	121.0
C(1)-C(2)-H(2A)	121.0
C(2)-C(3)-C(4)	120.9(5)
C(2)-C(3)-H(3A)	119.5
C(4)-C(3)-H(3A)	119.5
C(5)-C(4)-C(3)	121.3(5)
C(5)-C(4)-H(4A)	119.4
C(3)-C(4)-H(4A)	119.4
C(4)-C(5)-C(6)	118.3(5)
C(4)-C(5)-H(5A)	120.8
C(6)-C(5)-H(5A)	120.8
N(1)-C(6)-C(1)	114.8(4)
N(1)-C(6)-C(5)	124.9(5)
C(1)-C(6)-C(5)	120.3(5)
N(1)-C(7)-C(8)	121.5(4)
N(1)-C(7)-S(1)	115.3(4)
C(8)-C(7)-S(1)	123.3(4)
C(13)-C(8)-C(9)	117.1(5)
C(13)-C(8)-C(7)	124.2(5)
C(9)-C(8)-C(7)	118.7(5)
C(10)-C(9)-C(8)	121.7(5)
C(10)-C(9)-H(9A)	119.2
C(8)-C(9)-H(9A)	119.2
C(9)-C(10)-C(11)	120.0(5)
C(9)-C(10)-H(10A)	120.0
C(11)-C(10)-H(10A)	120.0
C(10)-C(11)-C(12)	119.9(5)
C(10)-C(11)-H(11A)	120.0
C(12)-C(11)-H(11A)	120.0
C(13)-C(12)-C(11)	119.3(5)

C(13)-C(12)-H(12A)	120.3
C(11)-C(12)-H(12A)	120.3
C(12)-C(13)-O(1)	122.2(4)
C(12)-C(13)-C(8)	122.0(4)
O(1)-C(13)-C(8)	115.8(4)
O(2)-C(14)-N(2)	122.3(5)
O(2)-C(14)-C(15)	123.0(5)
N(2)-C(14)-C(15)	114.7(4)
C(14)-C(15)-H(15A)	109.5
C(14)-C(15)-H(15B)	109.5
H(15A)-C(15)-H(15B)	109.5
C(14)-C(15)-H(15C)	109.5
H(15A)-C(15)-H(15C)	109.5
H(15B)-C(15)-H(15C)	109.5

Symmetry transformations used to generate equivalent atoms:

Figure S4 Molecular structure and atom numbering scheme for **6a.**

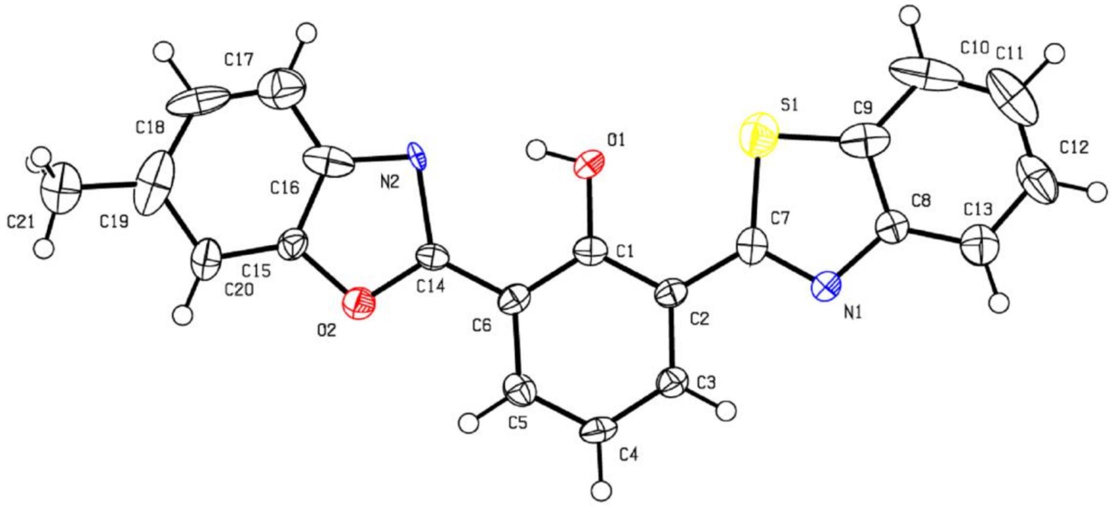

Table S3.1 Crystal data and structure refinement for **6a**.

Identification code	a	
Empirical formula	C21 H14 N2 O2 S	
Formula weight	358.40	
Temperature	150(2) K	
Wavelength	1.54178 Å	
Crystal system	Triclinic	
Space group	P-1	
Unit cell dimensions	a = 4.6897(7) Å	α= 109.35(2)°.
	b = 12.449(3) Å	β= 96.794(16)°.
	c = 15.119(4) Å	γ = 99.101(16)°.
Volume	808.4(3) $Å^3$	
Z	2	
Density (calculated)	1.472 Mg/m^3	
Absorption coefficient	1.935 mm^{-1}	
F(000)	372	
Crystal size	? x ? x ? mm^3	
Theta range for data collection	3.151 to 61.992°.	
Index ranges	-5<=h<=3, -13<=k<=14, -16<=l<=17	
Reflections collected	3871	
Independent reflections	2528 [R(int) = 0.0455]	
Completeness to theta = 61.992°	98.9 %	
Refinement method	Full-matrix least-squares on F^2	
Data / restraints / parameters	2528 / 6 / 236	
Goodness-of-fit on F^2	1.188	
Final R indices [I>2sigma(I)]	R1 = 0.1127, wR2 = 0.2996	
R indices (all data)	R1 = 0.1509, wR2 = 0.3289	
Extinction coefficient	n/a	
Largest diff. peak and hole	2.164 and -0.499 $e.Å^{-3}$	

Table S3.2 Bond lengths [Å] and angles [°] for **6a**.

S(1)-C(9)	1.647(9)
S(1)-C(7)	1.726(7)
N(1)-C(7)	1.316(8)
N(1)-C(8)	1.383(8)
O(2)-C(14)	1.358(8)
O(2)-C(15)	1.386(7)
O(1)-C(1)	1.349(7)
O(1)-H(1A)	0.8501
N(2)-C(16)	1.374(10)
N(2)-C(14)	1.584(7)
C(1)-C(6)	1.407(9)
C(1)-C(2)	1.414(9)
C(2)-C(3)	1.388(9)
C(2)-C(7)	1.463(9)
C(3)-C(4)	1.373(9)
C(3)-H(3A)	0.9500
C(4)-C(5)	1.375(9)
C(4)-H(4A)	0.9500
C(5)-C(6)	1.413(9)
C(5)-H(5A)	0.9500
C(6)-C(14)	1.435(9)
C(8)-C(13)	1.395(10)
C(8)-C(9)	1.412(10)
C(9)-C(10)	1.415(13)
C(10)-C(11)	1.385(15)
C(10)-H(10A)	0.9500
C(11)-C(12)	1.336(14)
C(11)-H(11A)	0.9500
C(12)-C(13)	1.367(11)
C(12)-H(12A)	0.9500
C(13)-H(13A)	0.9500
C(15)-C(20)	1.364(10)
C(15)-C(16)	1.410(10)
C(16)-C(17)	1.353(12)
C(17)-C(18)	1.309(14)
C(17)-H(17A)	0.9500

C(18)-C(19)	1.450(14)
C(18)-H(18A)	0.9500
C(19)-C(20)	1.441(11)
C(19)-C(21)	1.449(12)
C(20)-H(20A)	0.9500
C(21)-H(21A)	0.9800
C(21)-H(21B)	0.9800
C(21)-H(21C)	0.9800
C(9)-S(1)-C(7)	90.7(3)
C(7)-N(1)-C(8)	109.3(5)
C(14)-O(2)-C(15)	106.0(5)
C(1)-O(1)-H(1A)	106.2
C(16)-N(2)-C(14)	95.5(5)
O(1)-C(1)-C(6)	121.3(5)
O(1)-C(1)-C(2)	118.6(6)
C(6)-C(1)-C(2)	120.1(6)
C(3)-C(2)-C(1)	118.5(6)
C(3)-C(2)-C(7)	119.4(6)
C(1)-C(2)-C(7)	122.1(6)
C(4)-C(3)-C(2)	121.9(6)
C(4)-C(3)-H(3A)	119.0
C(2)-C(3)-H(3A)	119.0
C(3)-C(4)-C(5)	120.1(6)
C(3)-C(4)-H(4A)	119.9
C(5)-C(4)-H(4A)	119.9
C(4)-C(5)-C(6)	120.5(6)
C(4)-C(5)-H(5A)	119.8
C(6)-C(5)-H(5A)	119.7
C(5)-C(6)-C(1)	118.8(6)
C(5)-C(6)-C(14)	120.8(6)
C(1)-C(6)-C(14)	120.4(6)
N(1)-C(7)-C(2)	121.0(6)
N(1)-C(7)-S(1)	115.1(5)
C(2)-C(7)-S(1)	123.8(5)
N(1)-C(8)-C(13)	126.1(6)
N(1)-C(8)-C(9)	114.4(6)
C(13)-C(8)-C(9)	119.5(7)

C(8)-C(9)-C(10)	119.5(8)
C(8)-C(9)-S(1)	110.6(6)
C(10)-C(9)-S(1)	129.9(7)
C(11)-C(10)-C(9)	117.6(8)
C(11)-C(10)-H(10A)	121.2
C(9)-C(10)-H(10A)	121.2
C(12)-C(11)-C(10)	122.3(9)
C(12)-C(11)-H(11A)	118.9
C(10)-C(11)-H(11A)	118.9
C(11)-C(12)-C(13)	121.8(9)
C(11)-C(12)-H(12A)	119.1
C(13)-C(12)-H(12A)	119.1
C(12)-C(13)-C(8)	119.2(8)
C(12)-C(13)-H(13A)	120.4
C(8)-C(13)-H(13A)	120.4
O(2)-C(14)-C(6)	120.3(5)
O(2)-C(14)-N(2)	113.5(5)
C(6)-C(14)-N(2)	126.1(5)
C(20)-C(15)-O(2)	128.5(6)
C(20)-C(15)-C(16)	123.5(7)
O(2)-C(15)-C(16)	108.0(6)
C(17)-C(16)-N(2)	119.5(7)
C(17)-C(16)-C(15)	123.4(8)
N(2)-C(16)-C(15)	117.0(6)
C(18)-C(17)-C(16)	114.1(8)
C(18)-C(17)-H(17A)	122.9
C(16)-C(17)-H(17A)	123.0
C(17)-C(18)-C(19)	127.5(8)
C(17)-C(18)-H(18A)	116.3
C(19)-C(18)-H(18A)	116.2
C(20)-C(19)-C(18)	116.9(8)
C(20)-C(19)-C(21)	121.5(9)
C(18)-C(19)-C(21)	121.6(9)
C(15)-C(20)-C(19)	114.6(7)
C(15)-C(20)-H(20A)	122.7
C(19)-C(20)-H(20A)	122.7
C(19)-C(21)-H(21A)	109.5
C(19)-C(21)-H(21B)	109.5

H(21A)-C(21)-H(21B)	109.5
C(19)-C(21)-H(21C)	109.5
H(21A)-C(21)-H(21C)	109.5
H(21B)-C(21)-H(21C)	109.5

Symmetry transformations used to generate equivalent atoms:

Figure S5 Molecular structure and atom numbering scheme for **rhodation species.**

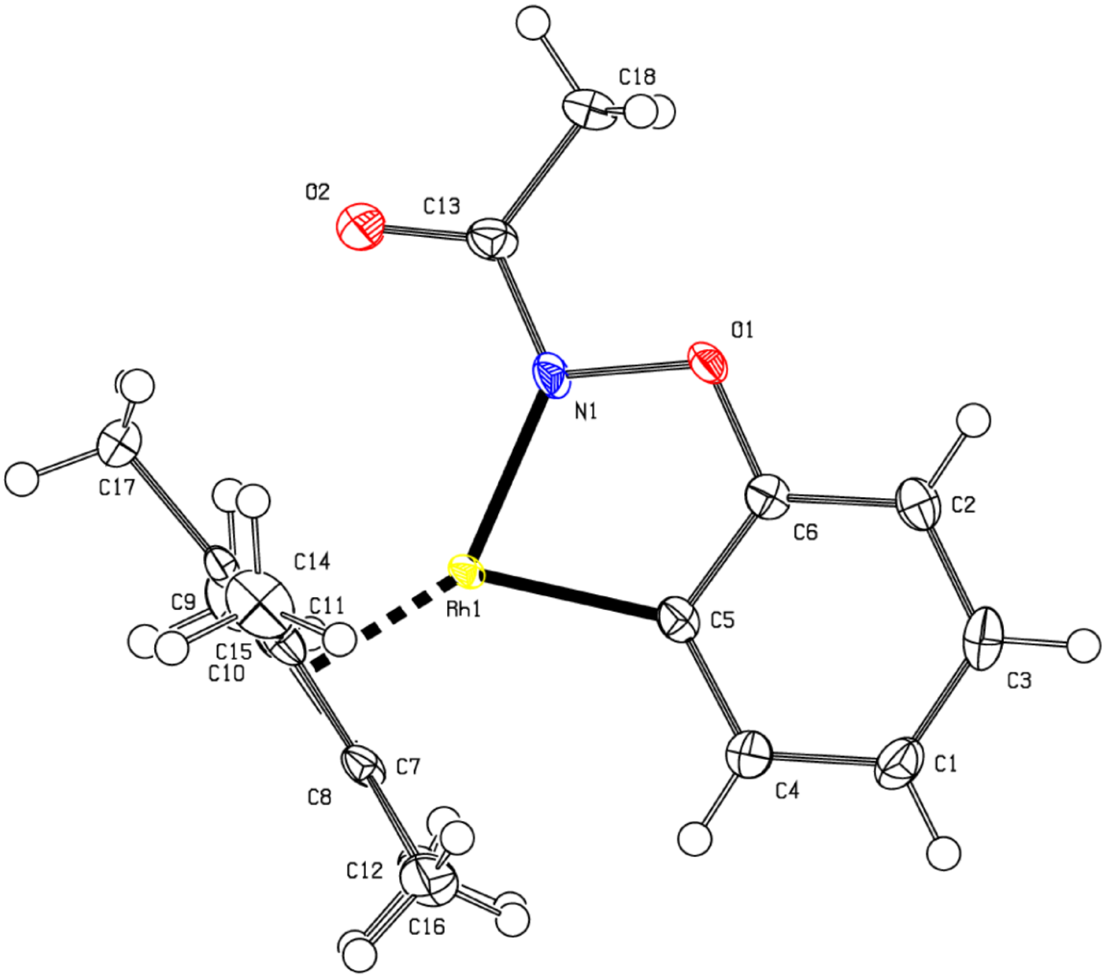

Table S4.1 Crystal data and structure refinement for **rhodation species**

Identification code	p
Empirical formula	C18 H22 N O2 Rh
Formula weight	387.28
Temperature	293(2) K
Wavelength	1.54178 A
Crystal system, space group	Monoclinic, P2(1)/c
Unit cell dimensions	a = 7.6555(3) A alpha = 90 deg.
	b = 13.0617(5) A beta = 96.793(3) deg.
	c = 16.9622(5) A gamma = 90 deg.
Volume	1684.21(10) A^3
Z, Calculated density	4, 1.527 Mg/m^3
Absorption coefficient	8.244 mm^-1
F(000)	792
Crystal size	0.25 x 0.21 x 0.19 mm
Theta range for data collection	4.28 to 67.09 deg.
Limiting indices	-8<=h<=9, -15<=k<=15, -20<=l<=20
Reflections collected / unique	19846 / 2989 [R(int) = 0.0658]
Completeness to theta = 67.09	99.4 %
Absorption correction	Semi-empirical from equivalents
Max. and min. transmission	0.3034 and 0.2324
Refinement method	Full-matrix least-squares on F^2
Data / restraints / parameters	2989 / 6 / 199
Goodness-of-fit on F^2	1.028
Final R indices [I>2sigma(I)]	R1 = 0.0425, wR2 = 0.1095
R indices (all data)	R1 = 0.0451, wR2 = 0.1137
Largest diff. peak and hole	1.296 and -1.656 e.A^-3

Table S4.2 Bond lengths [A] and angles [deg] for **rhodation species.**

Rh(1)-N(1)	1.975(2)
Rh(1)-C(5)	2.004(3)
Rh(1)-C(8)	2.141(2)
Rh(1)-C(7)	2.159(3)
Rh(1)-C(11)	2.168(3)
Rh(1)-C(10)	2.190(2)
Rh(1)-C(9)	2.260(2)
N(1)-C(13)	1.375(3)
N(1)-O(1)	1.448(3)
O(1)-C(6)	1.356(3)
O(2)-C(13)	1.223(3)
C(18)-C(13)	1.504(4)
C(18)-H(18A)	0.9600
C(18)-H(18B)	0.9600
C(18)-H(18C)	0.9600
C(1)-C(4)	1.389(4)
C(1)-C(3)	1.392(4)
C(1)-H(1A)	0.9300
C(2)-C(3)	1.381(4)
C(2)-C(6)	1.391(4)
C(2)-H(2A)	0.9300
C(3)-H(3A)	0.9300
C(4)-C(5)	1.411(4)
C(4)-H(4A)	0.9300
C(5)-C(6)	1.405(4)
C(7)-C(8)	1.423(4)
C(7)-C(11)	1.449(4)
C(7)-C(16)	1.491(4)
C(8)-C(10)	1.452(4)
C(8)-C(12)	1.495(4)
C(9)-C(11)	1.421(4)
C(9)-C(10)	1.422(4)
C(9)-C(17)	1.492(4)
C(10)-C(15)	1.482(4)
C(11)-C(14)	1.492(4)

C(12)-H(12A)	0.9600
C(12)-H(12B)	0.9600
C(12)-H(12C)	0.9600
C(14)-H(14A)	0.9600
C(14)-H(14B)	0.9600
C(14)-H(14C)	0.9600
C(15)-H(15A)	0.9600
C(15)-H(15B)	0.9600
C(15)-H(15C)	0.9600
C(16)-H(16A)	0.9600
C(16)-H(16B)	0.9600
C(16)-H(16C)	0.9600
C(17)-H(17A)	0.9600
C(17)-H(17B)	0.9600
C(17)-H(17C)	0.9600
N(1)-Rh(1)-C(5)	79.23(10)
N(1)-Rh(1)-C(8)	159.06(10)
C(5)-Rh(1)-C(8)	105.71(10)
N(1)-Rh(1)-C(7)	160.98(10)
C(5)-Rh(1)-C(7)	104.36(10)
C(8)-Rh(1)-C(7)	38.66(10)
N(1)-Rh(1)-C(11)	126.35(10)
C(5)-Rh(1)-C(11)	134.16(11)
C(8)-Rh(1)-C(11)	64.78(10)
C(7)-Rh(1)-C(11)	39.13(11)
N(1)-Rh(1)-C(10)	125.20(9)
C(5)-Rh(1)-C(10)	137.51(10)
C(8)-Rh(1)-C(10)	39.17(10)
C(7)-Rh(1)-C(10)	64.50(9)
C(11)-Rh(1)-C(10)	63.27(9)
N(1)-Rh(1)-C(9)	112.51(9)
C(5)-Rh(1)-C(9)	168.11(10)
C(8)-Rh(1)-C(9)	64.09(9)
C(7)-Rh(1)-C(9)	63.95(10)
C(11)-Rh(1)-C(9)	37.35(10)
C(10)-Rh(1)-C(9)	37.23(10)
C(13)-N(1)-O(1)	109.14(19)

C(13)-N(1)-Rh(1)	132.81(17)
O(1)-N(1)-Rh(1)	118.03(14)
C(6)-O(1)-N(1)	109.45(18)
C(13)-C(18)-H(18A)	109.5
C(13)-C(18)-H(18B)	109.5
H(18A)-C(18)-H(18B)	109.5
C(13)-C(18)-H(18C)	109.5
H(18A)-C(18)-H(18C)	109.5
H(18B)-C(18)-H(18C)	109.5
C(4)-C(1)-C(3)	120.5(3)
C(4)-C(1)-H(1A)	119.7
C(3)-C(1)-H(1A)	119.7
C(3)-C(2)-C(6)	118.7(3)
C(3)-C(2)-H(2A)	120.6
C(6)-C(2)-H(2A)	120.6
C(2)-C(3)-C(1)	120.1(3)
C(2)-C(3)-H(3A)	119.9
C(1)-C(3)-H(3A)	119.9
C(1)-C(4)-C(5)	121.2(3)
C(1)-C(4)-H(4A)	119.4
C(5)-C(4)-H(4A)	119.4
C(6)-C(5)-C(4)	116.0(2)
C(6)-C(5)-Rh(1)	113.43(18)
C(4)-C(5)-Rh(1)	130.5(2)
O(1)-C(6)-C(2)	117.0(2)
O(1)-C(6)-C(5)	119.6(2)
C(2)-C(6)-C(5)	123.4(2)
C(8)-C(7)-C(11)	106.9(2)
C(8)-C(7)-C(16)	127.7(3)
C(11)-C(7)-C(16)	125.3(3)
C(8)-C(7)-Rh(1)	69.96(14)
C(11)-C(7)-Rh(1)	70.77(15)
C(16)-C(7)-Rh(1)	126.89(19)
C(7)-C(8)-C(10)	107.6(2)
C(7)-C(8)-C(12)	127.3(2)
C(10)-C(8)-C(12)	125.0(2)
C(7)-C(8)-Rh(1)	71.38(14)
C(10)-C(8)-Rh(1)	72.24(14)

C(12)-C(8)-Rh(1)	125.02(18)
C(11)-C(9)-C(10)	107.0(2)
C(11)-C(9)-C(17)	127.1(3)
C(10)-C(9)-C(17)	125.8(2)
C(11)-C(9)-Rh(1)	67.81(15)
C(10)-C(9)-Rh(1)	68.68(14)
C(17)-C(9)-Rh(1)	130.82(19)
C(9)-C(10)-C(8)	108.8(2)
C(9)-C(10)-C(15)	126.2(2)
C(8)-C(10)-C(15)	124.9(2)
C(9)-C(10)-Rh(1)	74.09(14)
C(8)-C(10)-Rh(1)	68.60(13)
C(15)-C(10)-Rh(1)	125.91(19)
C(9)-C(11)-C(7)	109.4(2)
C(9)-C(11)-C(14)	125.6(3)
C(7)-C(11)-C(14)	124.8(3)
C(9)-C(11)-Rh(1)	74.84(16)
C(7)-C(11)-Rh(1)	70.10(15)
C(14)-C(11)-Rh(1)	125.10(19)
C(8)-C(12)-H(12A)	109.5
C(8)-C(12)-H(12B)	109.5
H(12A)-C(12)-H(12B)	109.5
C(8)-C(12)-H(12C)	109.5
H(12A)-C(12)-H(12C)	109.5
H(12B)-C(12)-H(12C)	109.5
O(2)-C(13)-N(1)	119.3(2)
O(2)-C(13)-C(18)	121.6(2)
N(1)-C(13)-C(18)	119.1(2)
C(11)-C(14)-H(14A)	109.5
C(11)-C(14)-H(14B)	109.5
H(14A)-C(14)-H(14B)	109.5
C(11)-C(14)-H(14C)	109.5
H(14A)-C(14)-H(14C)	109.5
H(14B)-C(14)-H(14C)	109.5
C(10)-C(15)-H(15A)	109.5
C(10)-C(15)-H(15B)	109.5
H(15A)-C(15)-H(15B)	109.5
C(10)-C(15)-H(15C)	109.5

H(15A)-C(15)-H(15C)	109.5
H(15B)-C(15)-H(15C)	109.5
C(7)-C(16)-H(16A)	109.5
C(7)-C(16)-H(16B)	109.5
H(16A)-C(16)-H(16B)	109.5
C(7)-C(16)-H(16C)	109.5
H(16A)-C(16)-H(16C)	109.5
H(16B)-C(16)-H(16C)	109.5
C(9)-C(17)-H(17A)	109.5
C(9)-C(17)-H(17B)	109.5
H(17A)-C(17)-H(17B)	109.5
C(9)-C(17)-H(17C)	109.5
H(17A)-C(17)-H(17C)	109.5
H(17B)-C(17)-H(17C)	109.5

Symmetry transformations used to generate equivalent atoms:

IX. The excitation and emission spectras

Figure S6 a) The Normalized fluorescence spectra of 3aa in different solvents (2×10^{-6} mol L^{-1}, λ_{ex} = 330 nm); b) The fluorescence spectra of mono-subtituted products (Benzoxazoles) in DCM (2×10^{-6} mol L^{-1}, λ_{ex} = 330 nm); c) The normalized absorption spectra in DCM (2×10^{-6} mol L^{-1}) and fluorescence spectra of bis-subtituted products in DCM (2×10^{-6} mol L^{-1}, λ_{ex} = 360 nm) [2-4].

a)

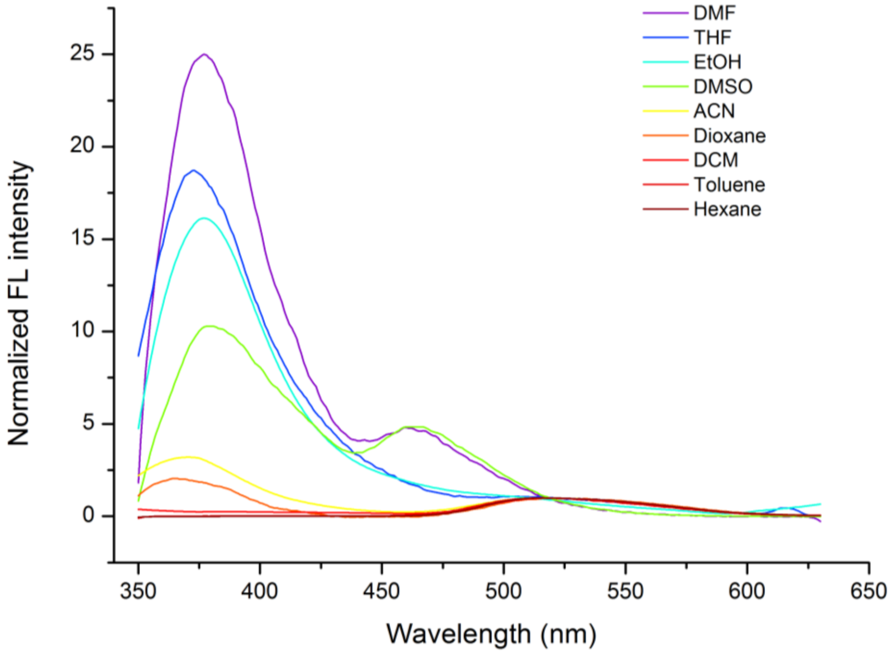

b)

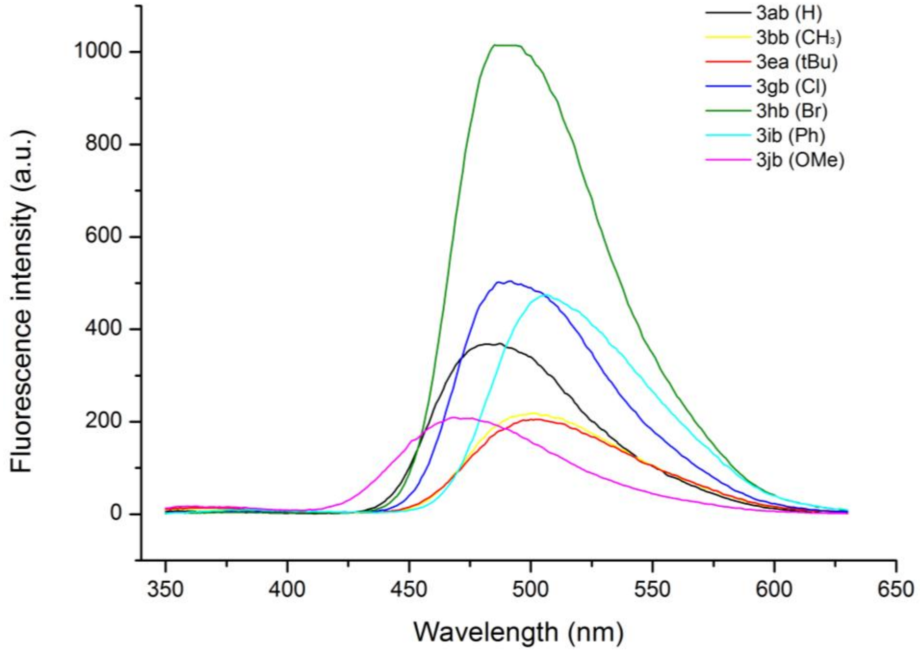

c)

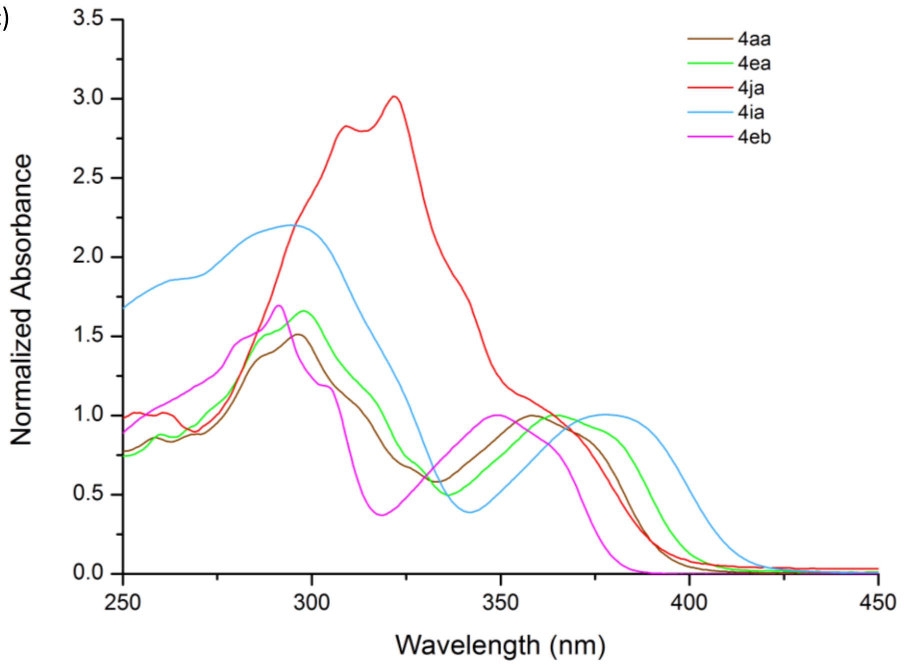

X. References

(1) Petrassi, H. M.; Sharpless, K. B.; Kelly, J. W. The copper-mediated cross-coupling of phenylboronic acids and N-hydroxyphthalimide at room temperature: synthesis of aryloxyamines. *Org.Lett.* **3**, 139-142 (2001).

(2) Kim, Y. H., Roh, S. G. & Cho, D. W. Excited-state intramolecular proton transfer on 2-(2'-hydroxy-4'-R-phenyl)benzothiazole nanoparticles and fluorescence wavelength depending on substituent and temperature. Photochem. Photobiol. Sci. **9**, 722-729 (2010).

(3) Wang, R., Liu, D., Xu, K. & Li, J. Substituent and solvent effects on excited state intramolecular proton transfer in novel 2-(2′-hydroxyphenyl)benzothiazole derivatives. *J. Photochem. Photobiol., A* **205**, 61-69 (2009).

XI. VII. ^{1}H and ^{13}C NMR spectra

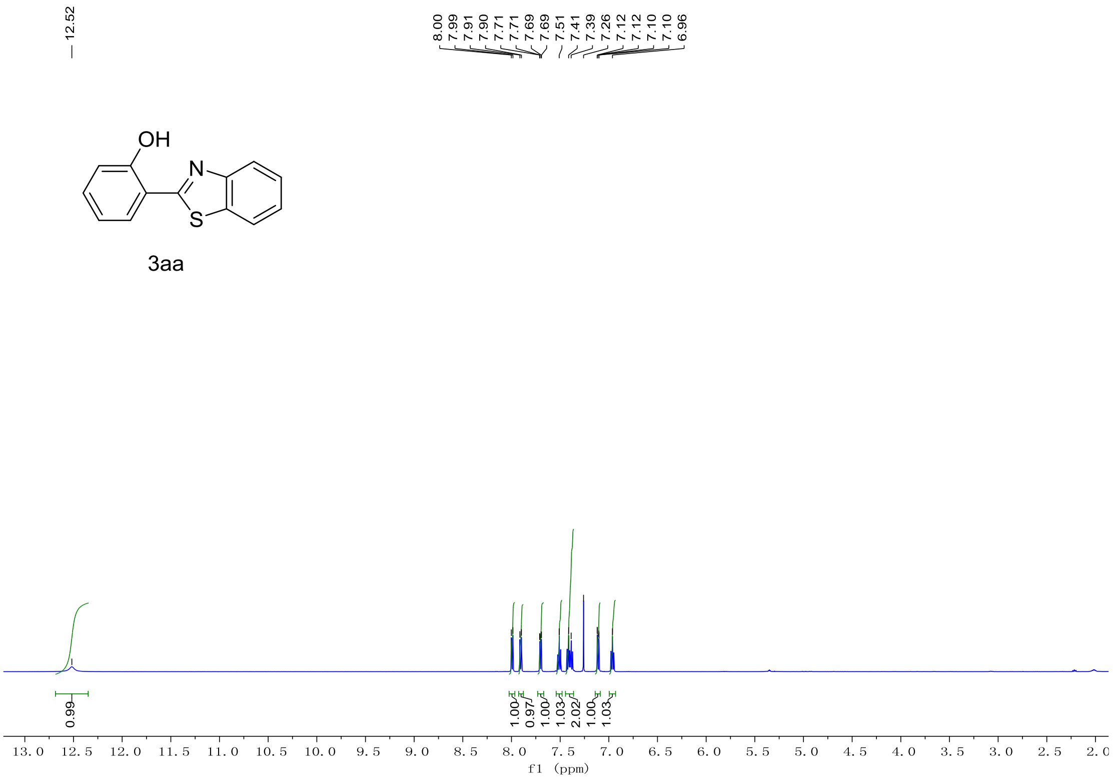

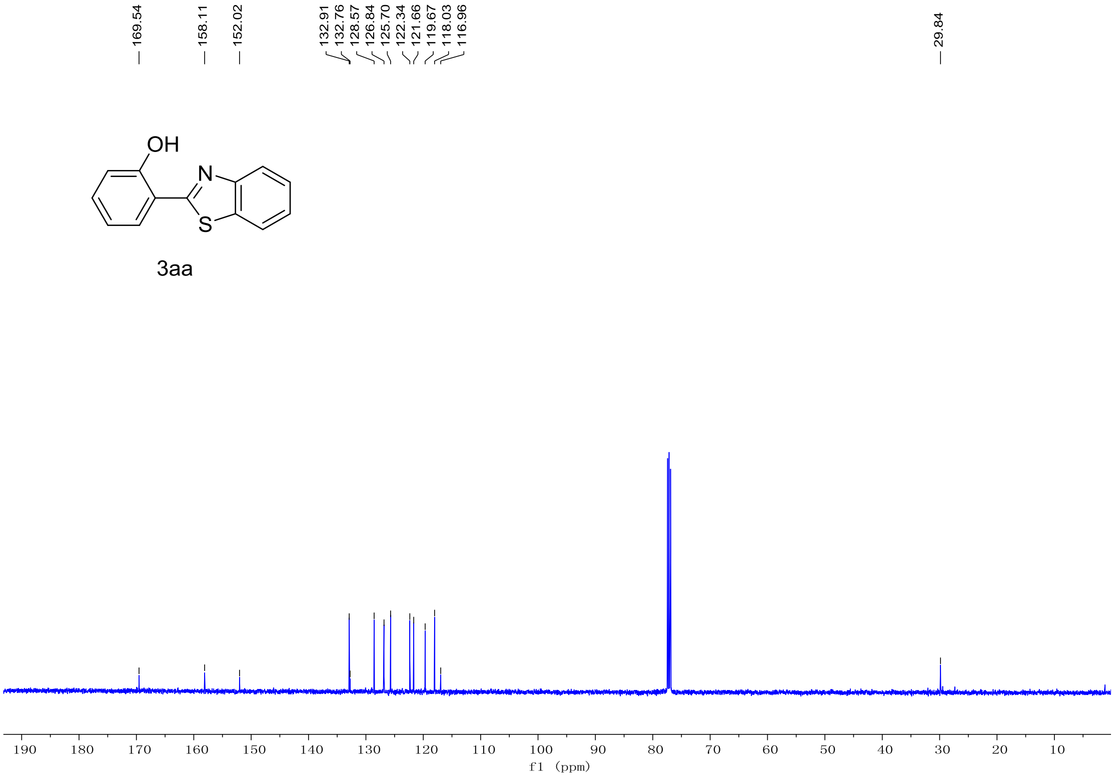

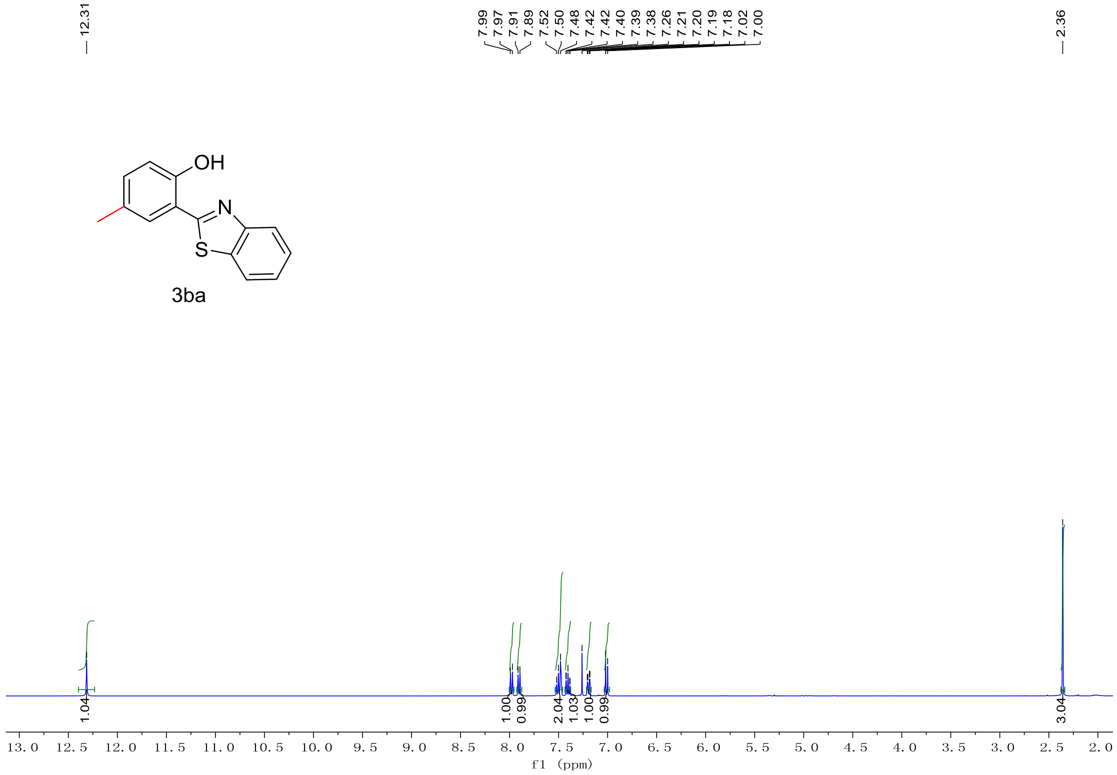
12.31
7.99
7.97
7.91
7.89
7.52
7.50
7.48
7.42
7.42
7.40
7.39
7.38
7.26
7.21
7.20
7.19
7.18
7.02
7.00
2.36
OH
N
S
3ba
1.04
1.00
0.99
2.04
1.03
1.00
0.99
3.04
f1 (ppm)

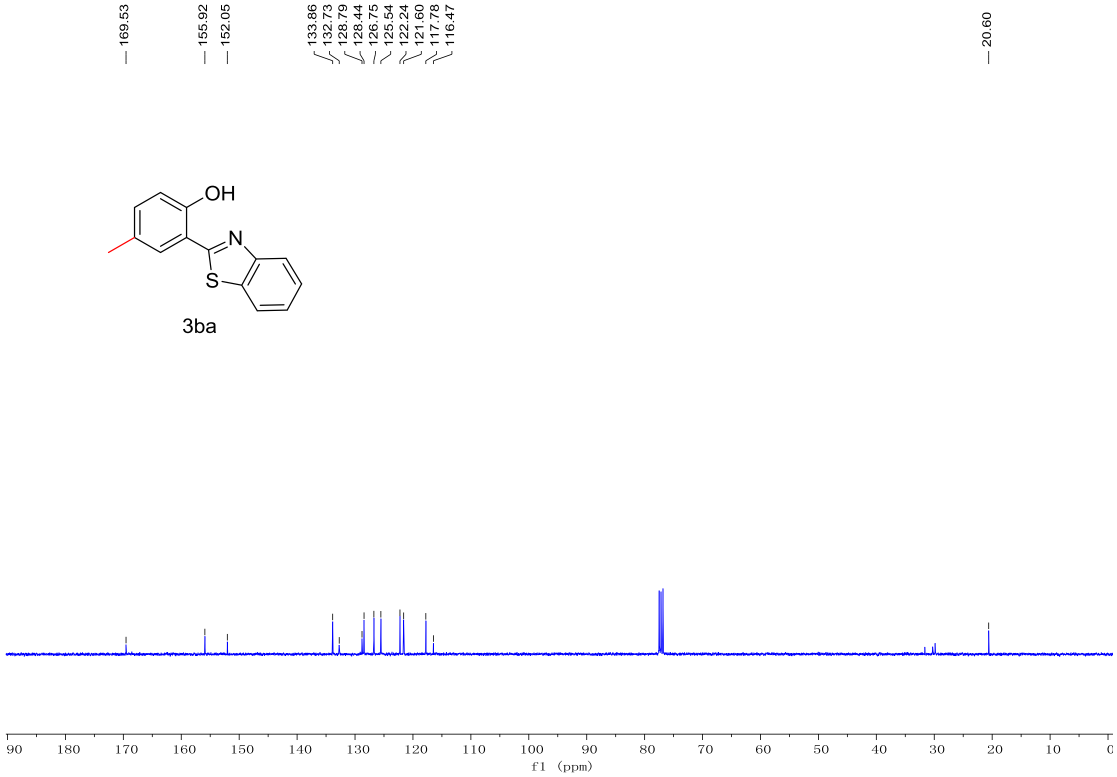
169.53
155.92
152.05
133.86
132.73
128.79
128.44
126.75
125.54
122.24
121.60
117.78
116.47
20.60
OH
N
S
3ba
f1 (ppm)

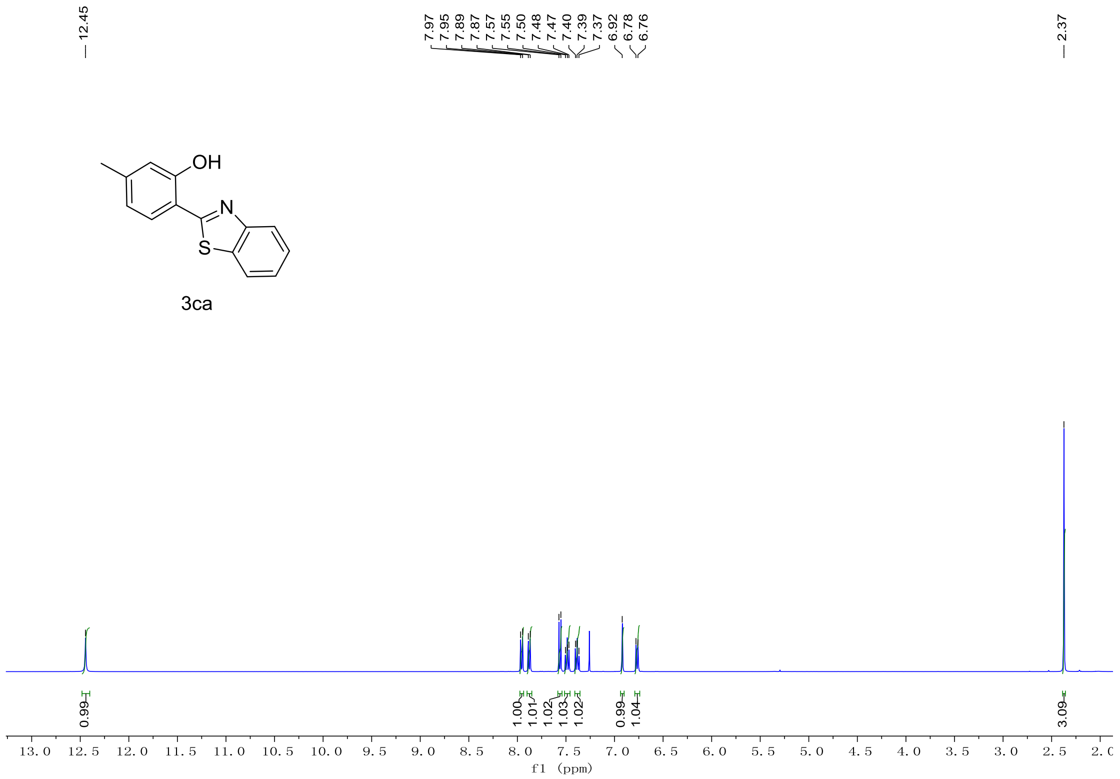
12.45
7.97
7.95
7.89
7.87
7.57
7.55
7.50
7.48
7.47
7.40
7.39
7.37
6.92
6.78
6.76
2.37
OH
N
S
3ca
0.99
1.00
1.01
1.02
1.03
1.02
0.99
1.04
3.09
f1 (ppm)

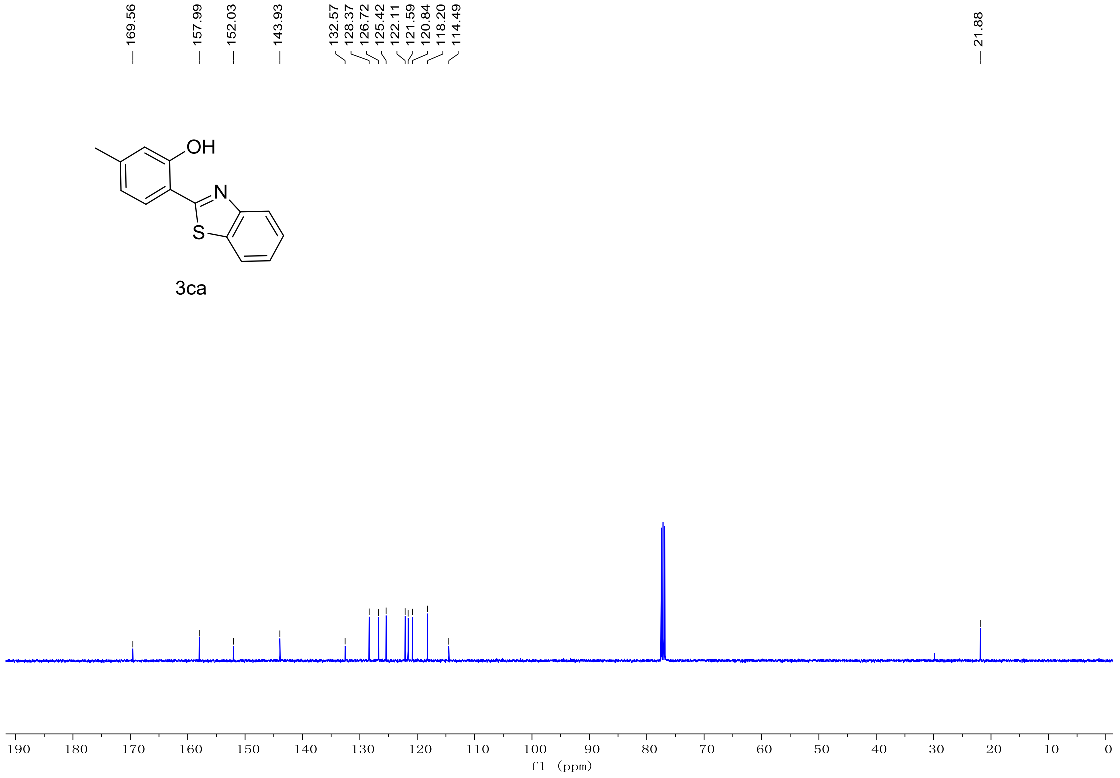
169.56
157.99
152.03
143.93
132.57
128.37
126.72
125.42
122.11
121.59
120.84
118.20
114.49
21.88
OH
N
S
3ca
f1 (ppm)

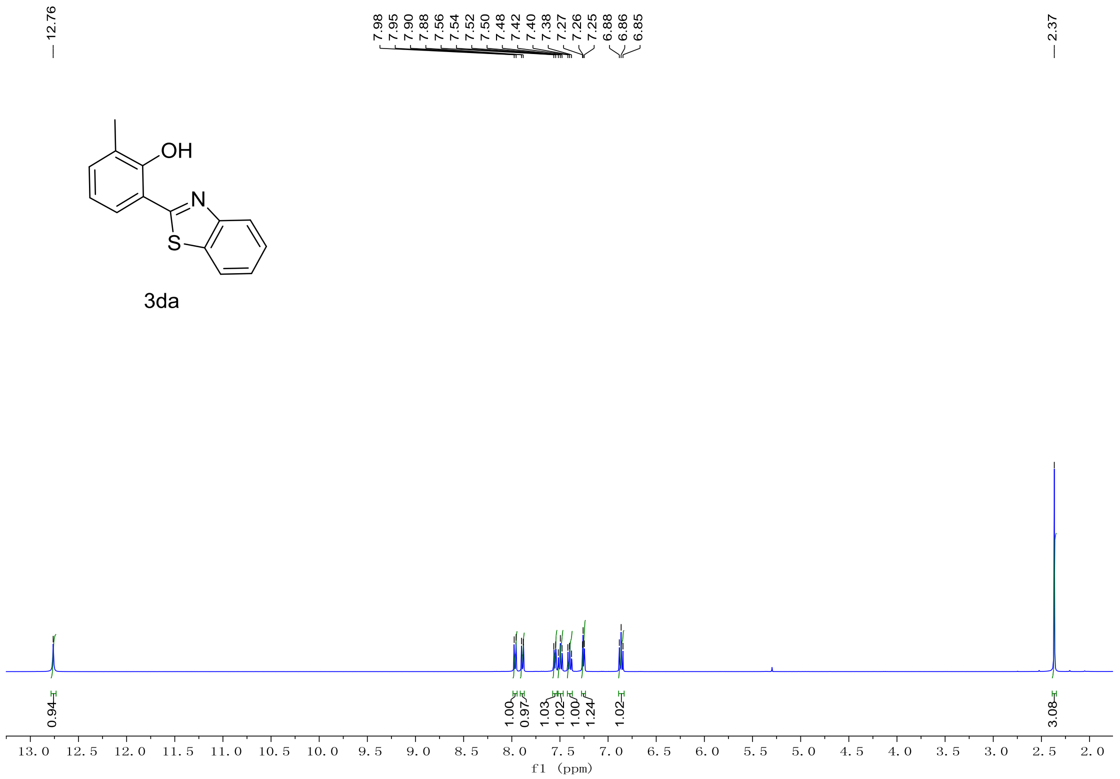
12.76
7.98
7.95
7.90
7.88
7.56
7.54
7.52
7.50
7.48
7.42
7.40
7.38
7.27
7.26
7.25
6.88
6.86
6.85
2.37
OH
N
S
3da
0.94
1.00
0.97
1.03
1.02
1.00
1.24
1.02
3.08
13.0 12.5 12.0 11.5 11.0 10.5 10.0 9.5 9.0 8.5 8.0 7.5 7.0 6.5 6.0 5.5 5.0 4.5 4.0 3.5 3.0 2.5 2.0
f1 (ppm)

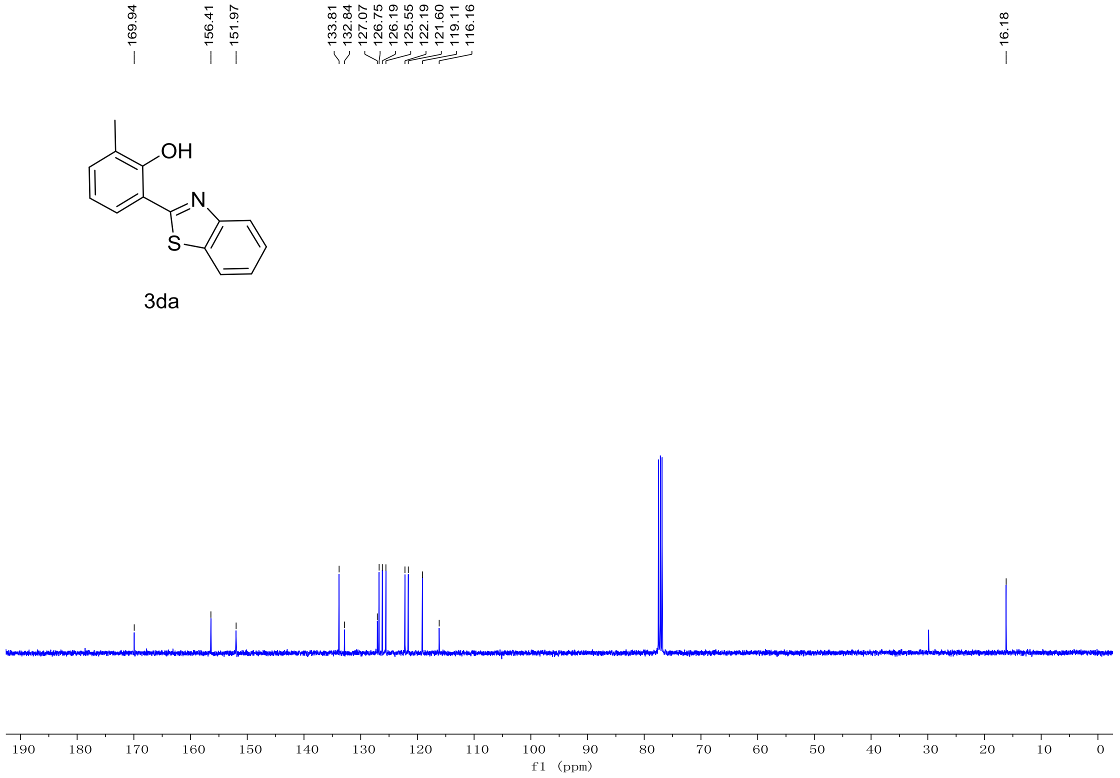
169.94
156.41
151.97
133.81
132.84
127.07
126.75
126.19
125.55
122.19
121.60
119.11
116.16
16.18
OH
N
S
3da
190 180 170 160 150 140 130 120 110 100 90 80 70 60 50 40 30 20 10 0
f1 (ppm)

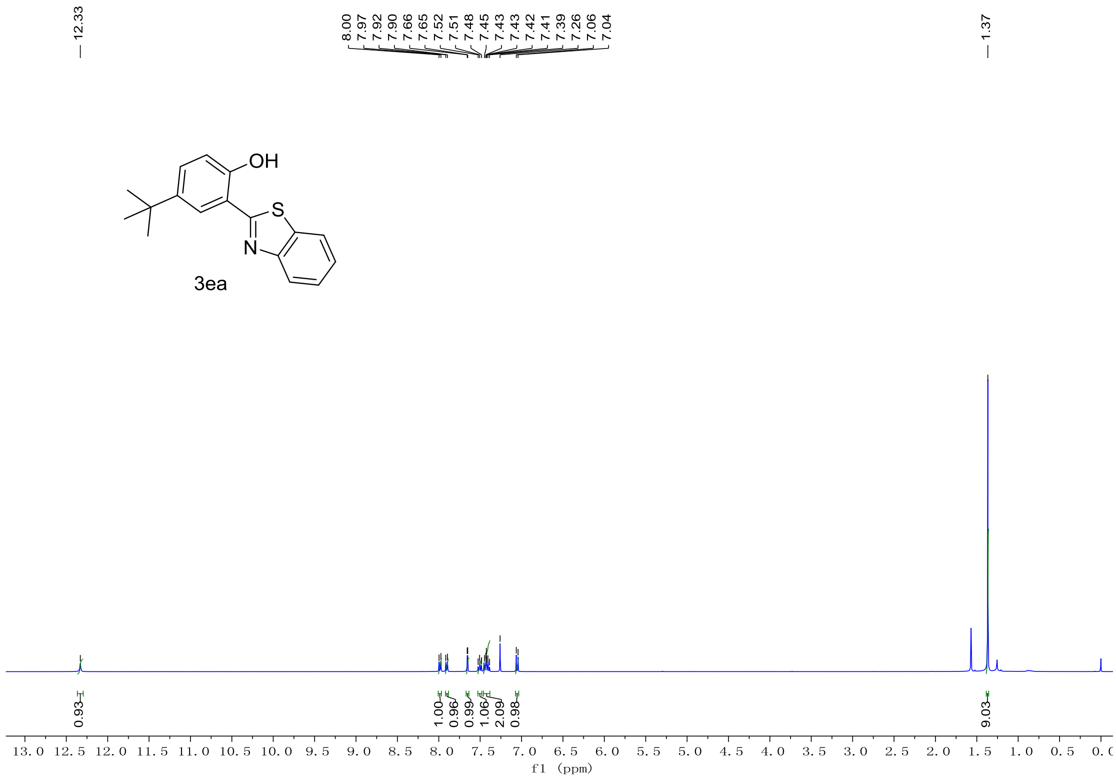
12.33
8.00
7.97
7.92
7.90
7.66
7.65
7.52
7.51
7.48
7.45
7.43
7.43
7.42
7.41
7.39
7.26
7.06
7.04
1.37
0.93
1.00
0.96
0.99
1.06
2.09
0.98
9.03
13.0 12.5 12.0 11.5 11.0 10.5 10.0 9.5 9.0 8.5 8.0 7.5 7.0 6.5 6.0 5.5 5.0 4.5 4.0 3.5 3.0 2.5 2.0 1.5 1.0 0.5 0.0
f1 (ppm)

3ea

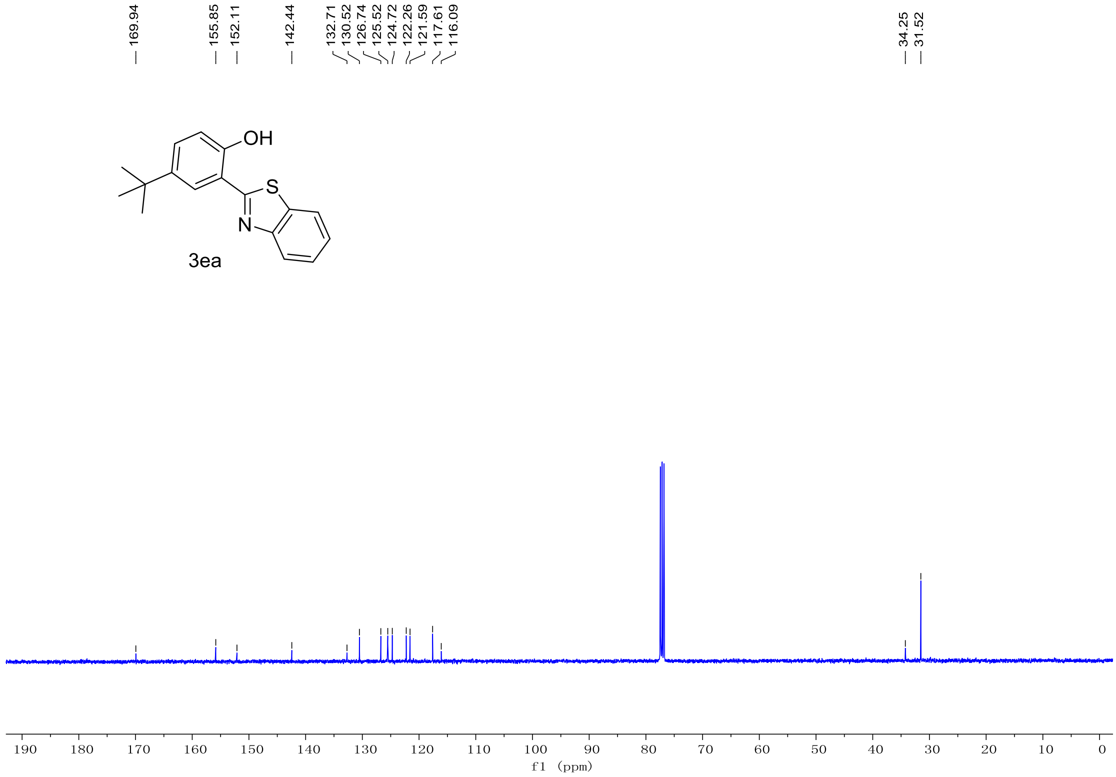
169.94
155.85
152.11
142.44
132.71
130.52
126.74
125.52
124.72
122.26
121.59
117.61
116.09
34.25
31.52
190 180 170 160 150 140 130 120 110 100 90 80 70 60 50 40 30 20 10 0
f1 (ppm)

3ea

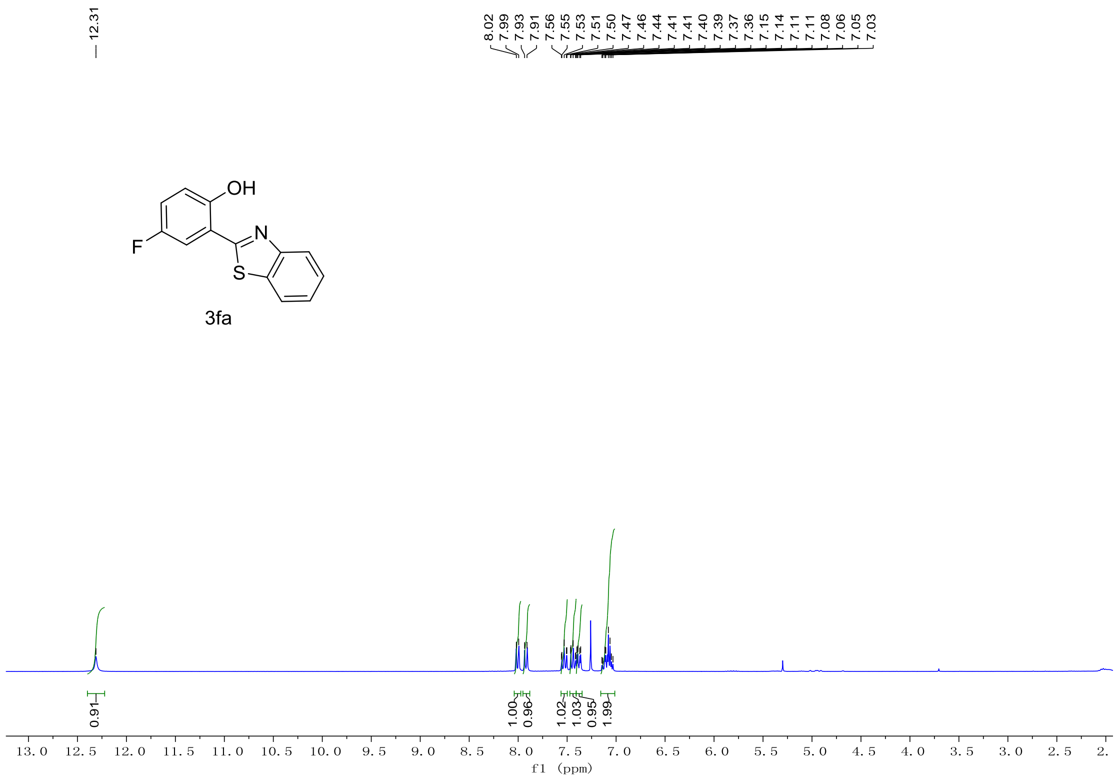

12.31
8.02
7.99
7.93
7.91
7.56
7.55
7.53
7.51
7.50
7.47
7.46
7.44
7.41
7.41
7.40
7.39
7.37
7.36
7.15
7.14
7.11
7.11
7.08
7.06
7.05
7.03
OH
F
N
S
3fa
0.91
1.00
0.96
1.02
1.03
0.95
1.99
f1 (ppm)

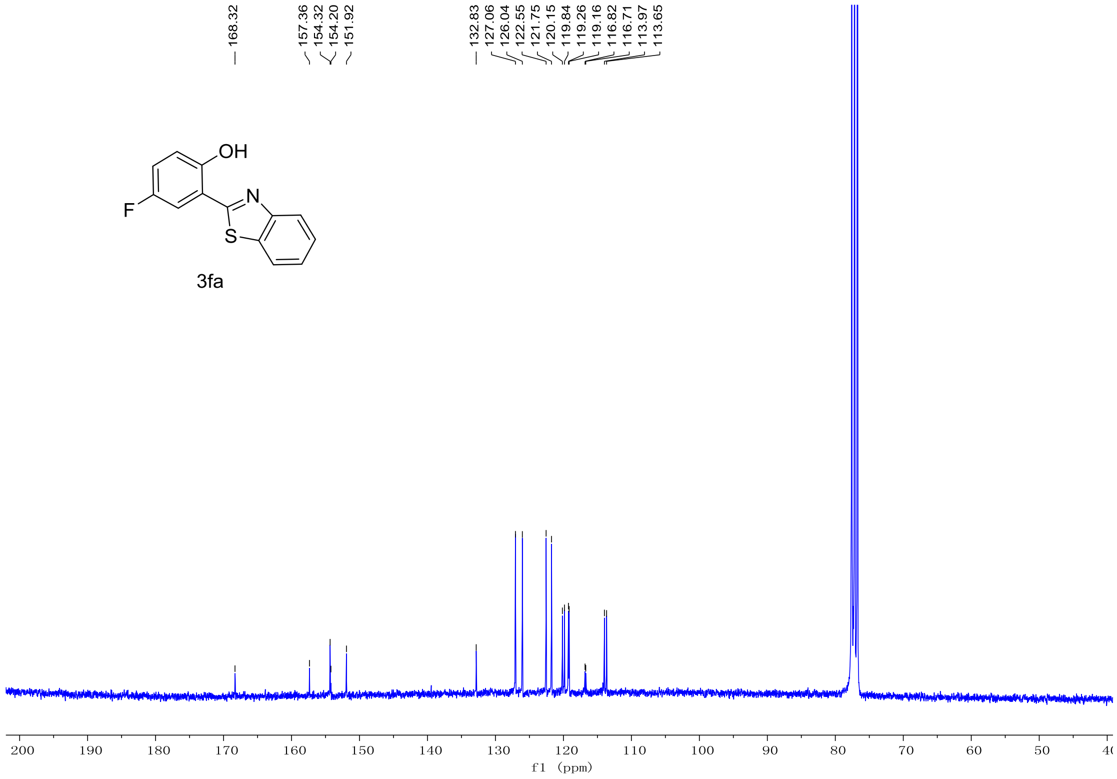

168.32
157.36
154.32
154.20
151.92
132.83
127.06
126.04
122.55
121.75
120.15
119.84
119.26
119.16
116.82
116.71
113.97
113.65
OH
F
N
S
3fa
f1 (ppm)

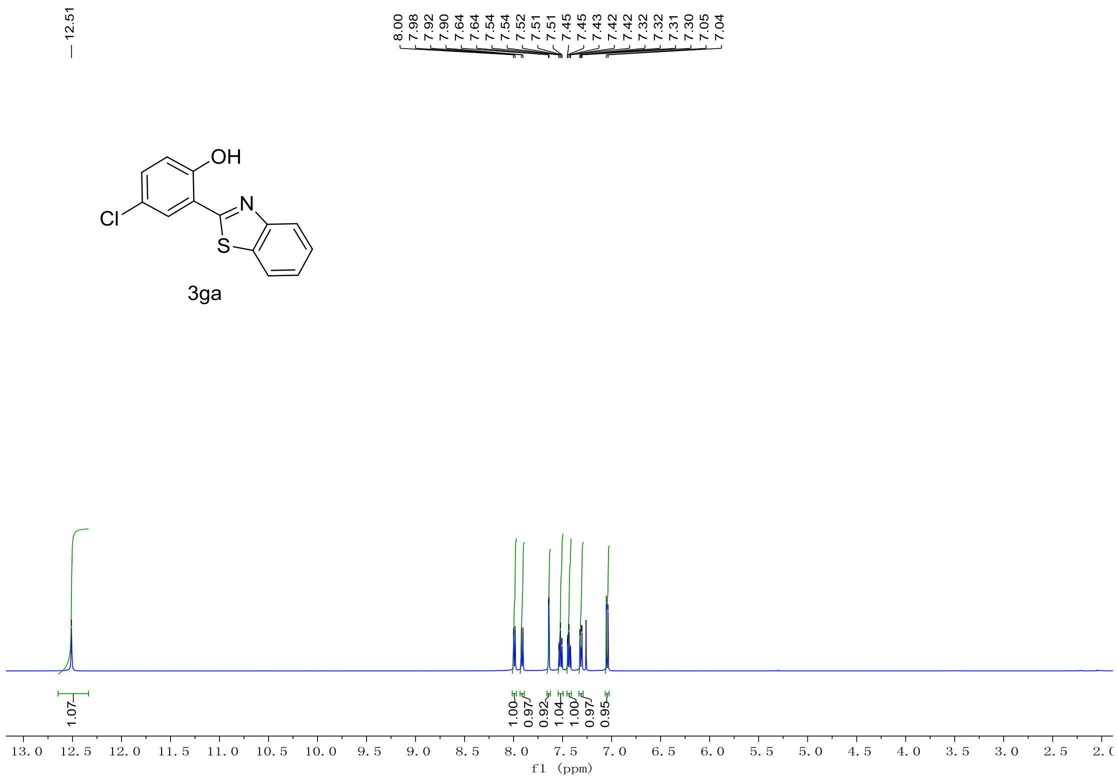
12.51
8.00
7.98
7.92
7.90
7.64
7.64
7.54
7.54
7.52
7.51
7.51
7.45
7.45
7.43
7.42
7.42
7.32
7.32
7.31
7.30
7.05
7.04
OH
N
Cl
S
3ga
1.07
1.00
0.97
0.92
1.04
1.00
0.97
0.95
13.0 12.5 12.0 11.5 11.0 10.5 10.0 9.5 9.0 8.5 8.0 7.5 7.0 6.5 6.0 5.5 5.0 4.5 4.0 3.5 3.0 2.5
f1 (ppm)

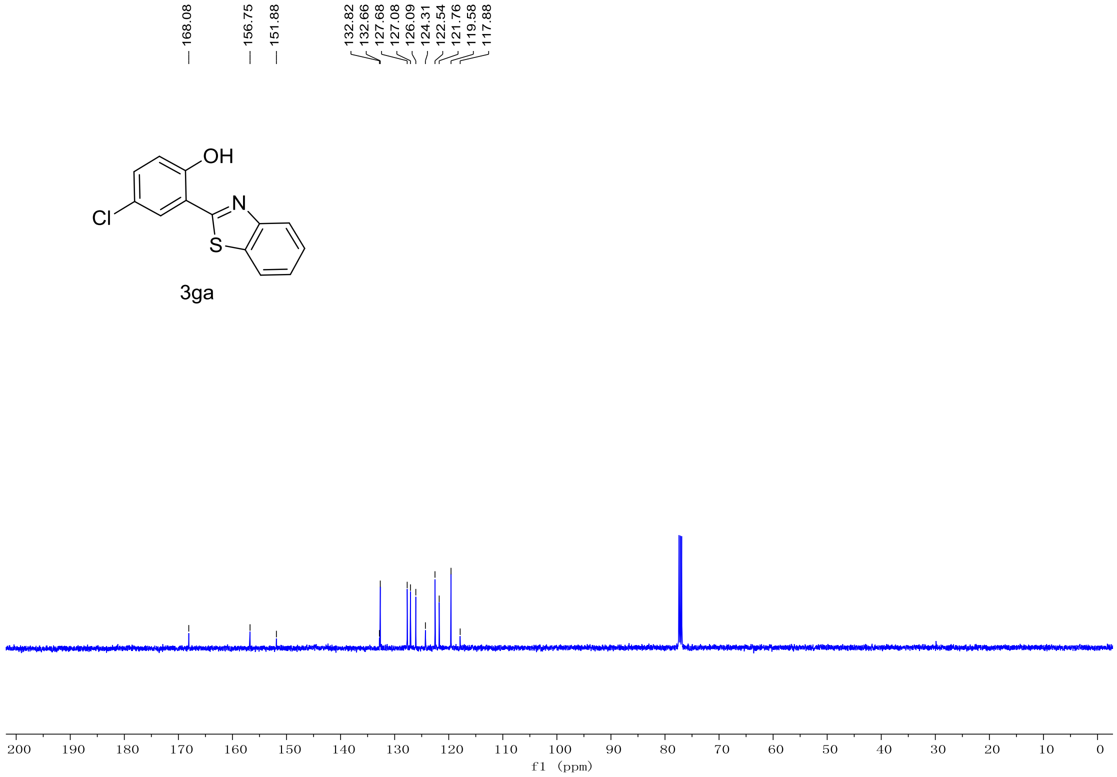
168.08
156.75
151.88
132.82
132.66
127.68
127.08
126.09
124.31
122.54
121.76
119.58
117.88
OH
N
Cl
S
3ga
200 190 180 170 160 150 140 130 120 110 100 90 80 70 60 50 40 30 20 10 0
f1 (ppm)

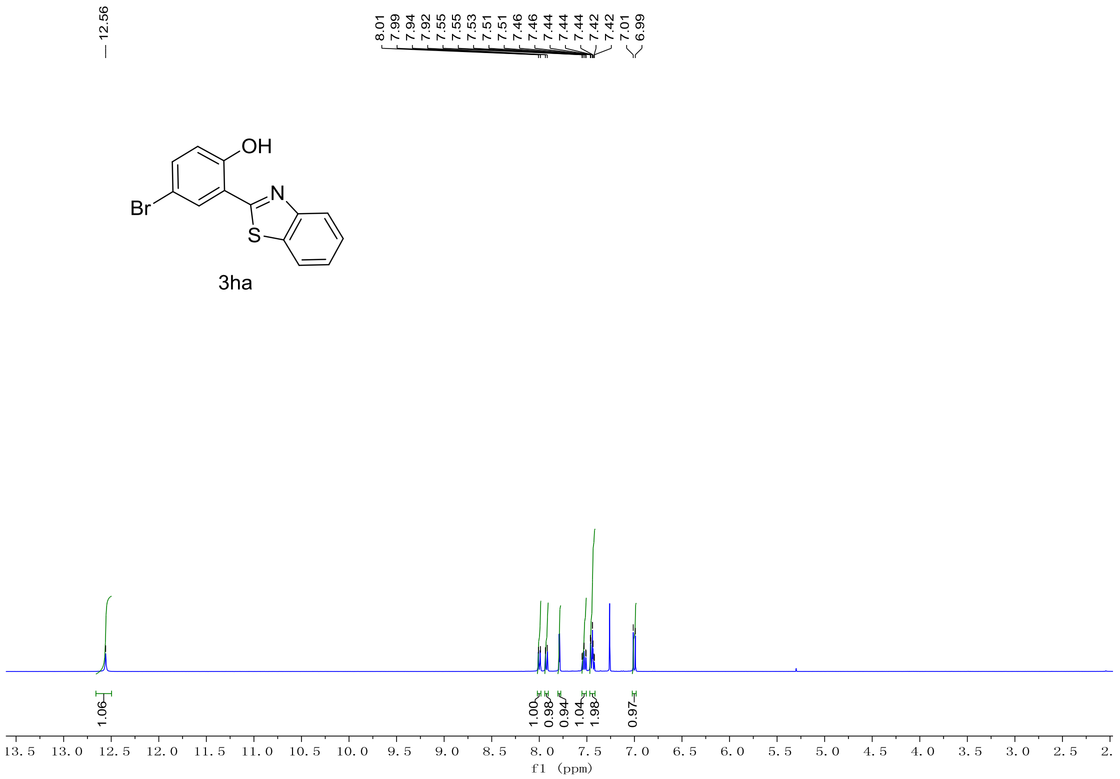
12.56
8.01
7.99
7.94
7.92
7.55
7.55
7.53
7.51
7.51
7.46
7.46
7.44
7.44
7.44
7.42
7.42
7.01
6.99
OH
Br
N
S
3ha
1.06
1.00
0.98
0.94
1.04
1.98
0.97
f1 (ppm)

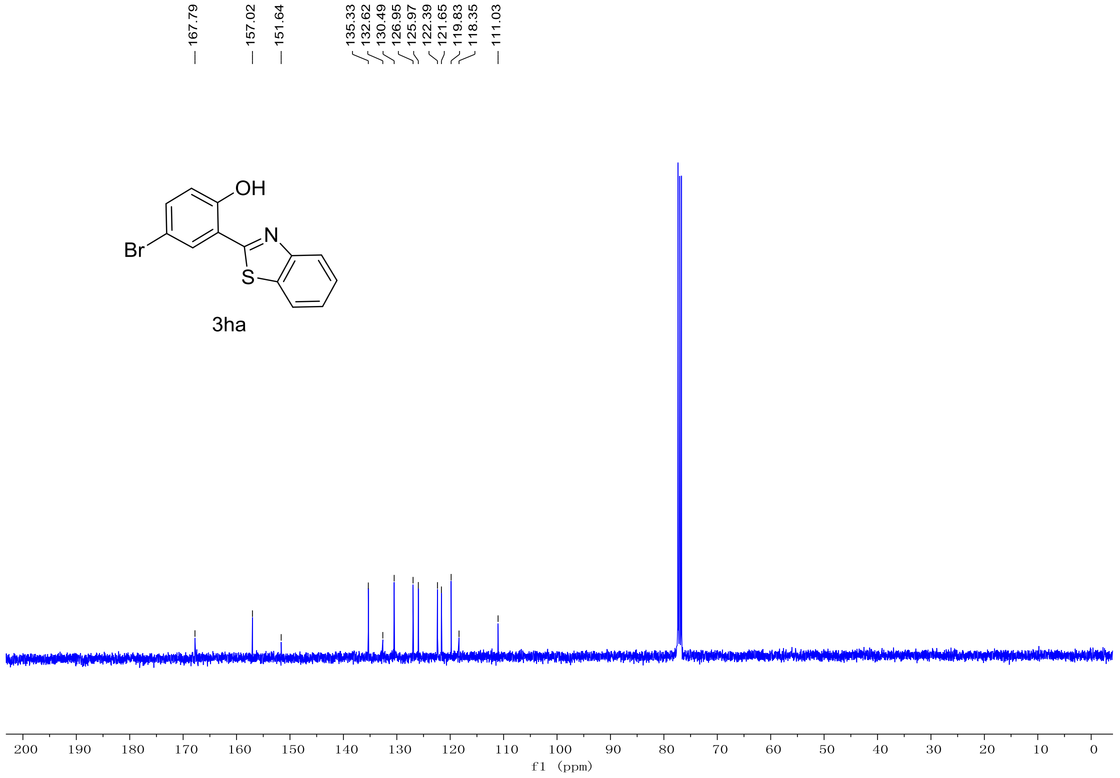
167.79
157.02
151.64
135.33
132.62
130.49
126.95
125.97
122.39
121.65
119.83
118.35
111.03
OH
Br
N
S
3ha
f1 (ppm)

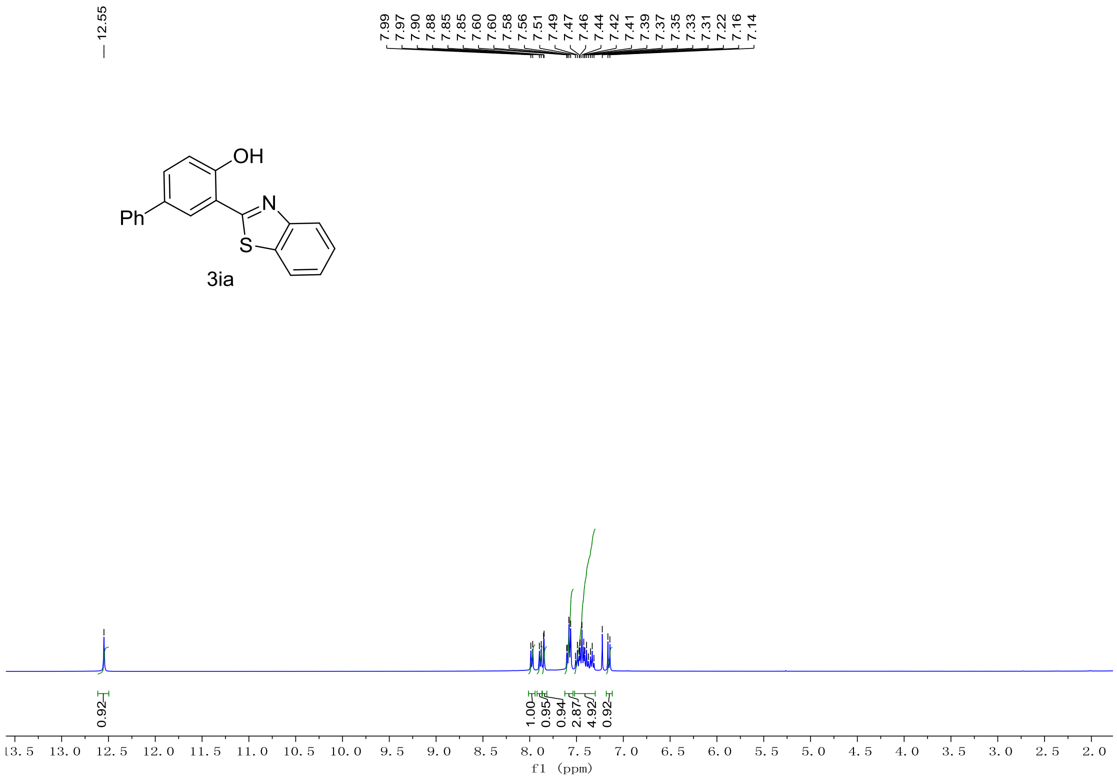
12.55
7.99 7.97 7.90 7.88 7.85 7.85 7.60 7.60 7.58 7.56 7.51 7.49 7.47 7.46 7.44 7.42 7.41 7.39 7.37 7.35 7.33 7.31 7.22 7.16 7.14
OH
Ph
N
S
3ia
0.92
1.00 0.95 0.94 2.87 4.92 0.92
f1 (ppm)

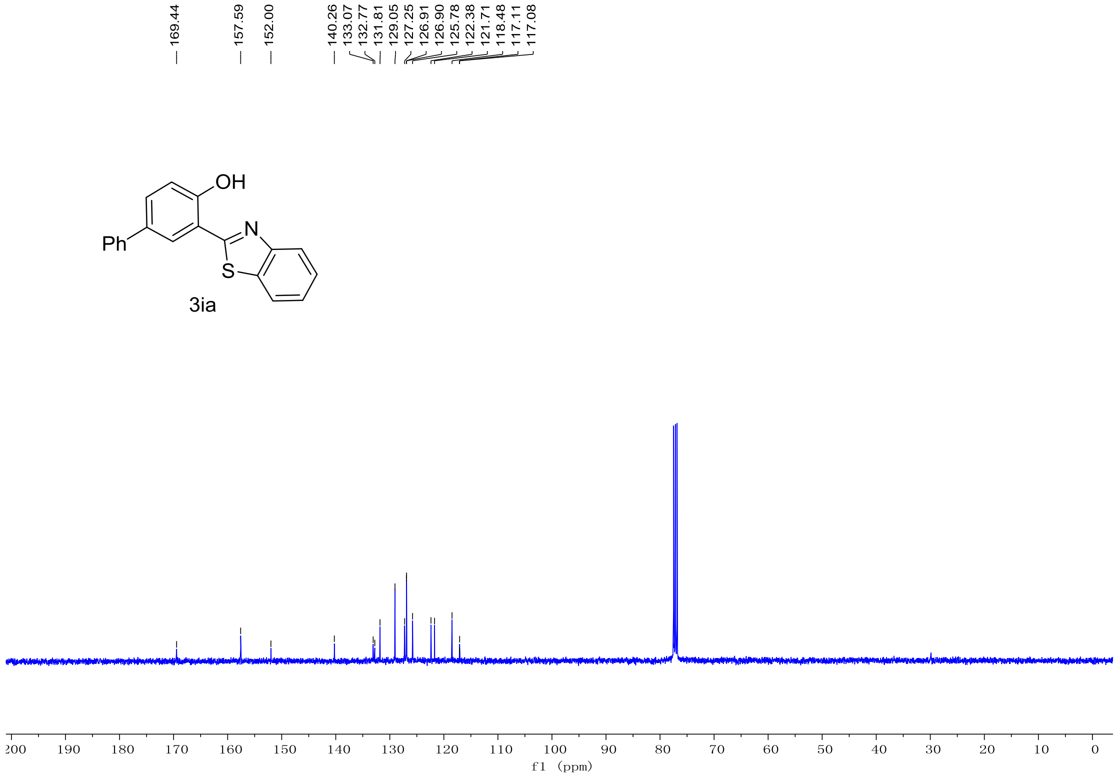
169.44
157.59
152.00
140.26 133.07 132.77 131.81 129.05 127.25 126.91 126.90 125.78 122.38 121.71 118.48 117.11 117.08
OH
Ph
N
S
3ia
f1 (ppm)

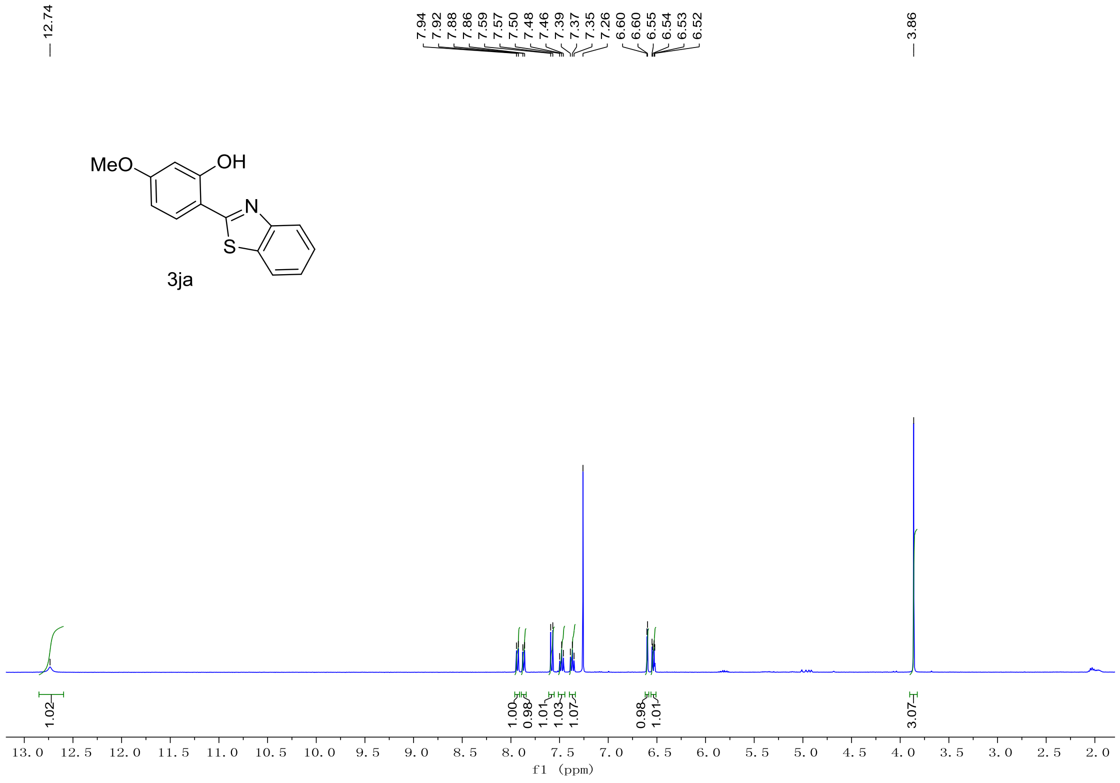
12.74
7.94
7.92
7.88
7.86
7.59
7.57
7.50
7.48
7.46
7.39
7.37
7.35
7.26
6.60
6.55
6.54
6.53
6.52
3.86
MeO
OH
N
S
3ja
1.02
1.00
0.98
1.01
1.03
1.07
0.98
1.01
3.07
f1 (ppm)

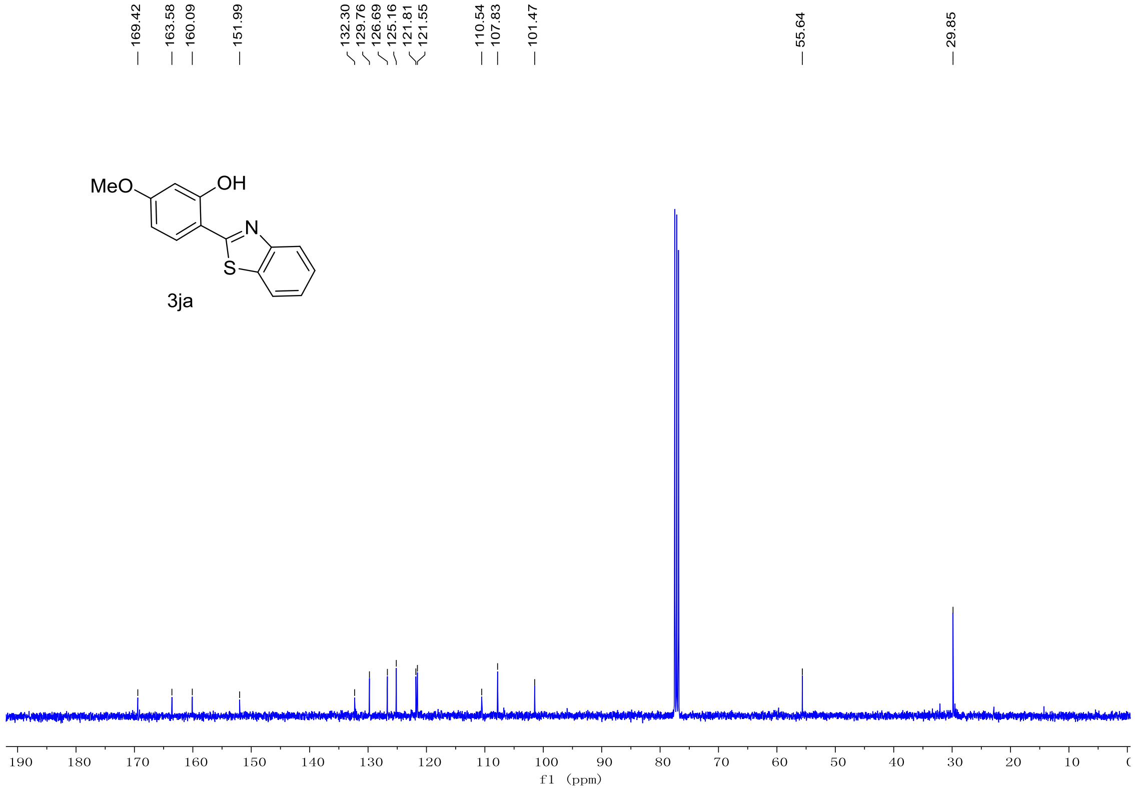
169.42
163.58
160.09
151.99
132.30
129.76
126.69
125.16
121.81
121.55
110.54
107.83
101.47
55.64
29.85
MeO
OH
N
S
3ja
f1 (ppm)

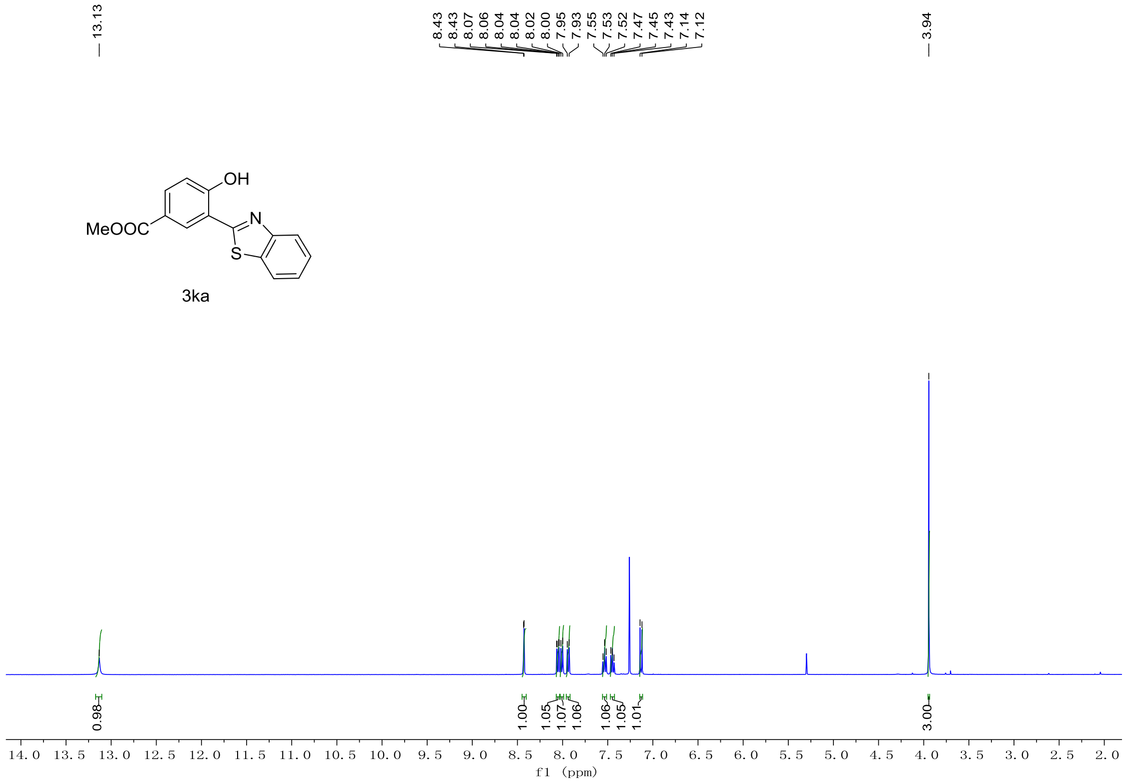
13.13
8.43
8.43
8.07
8.06
8.04
8.04
8.02
8.00
7.95
7.93
7.55
7.53
7.52
7.47
7.45
7.43
7.14
7.12
3.94
OH
MeOOC
N
S
3ka
0.98
1.00
1.05
1.07
1.06
1.06
1.05
1.01
3.00
f1 (ppm)

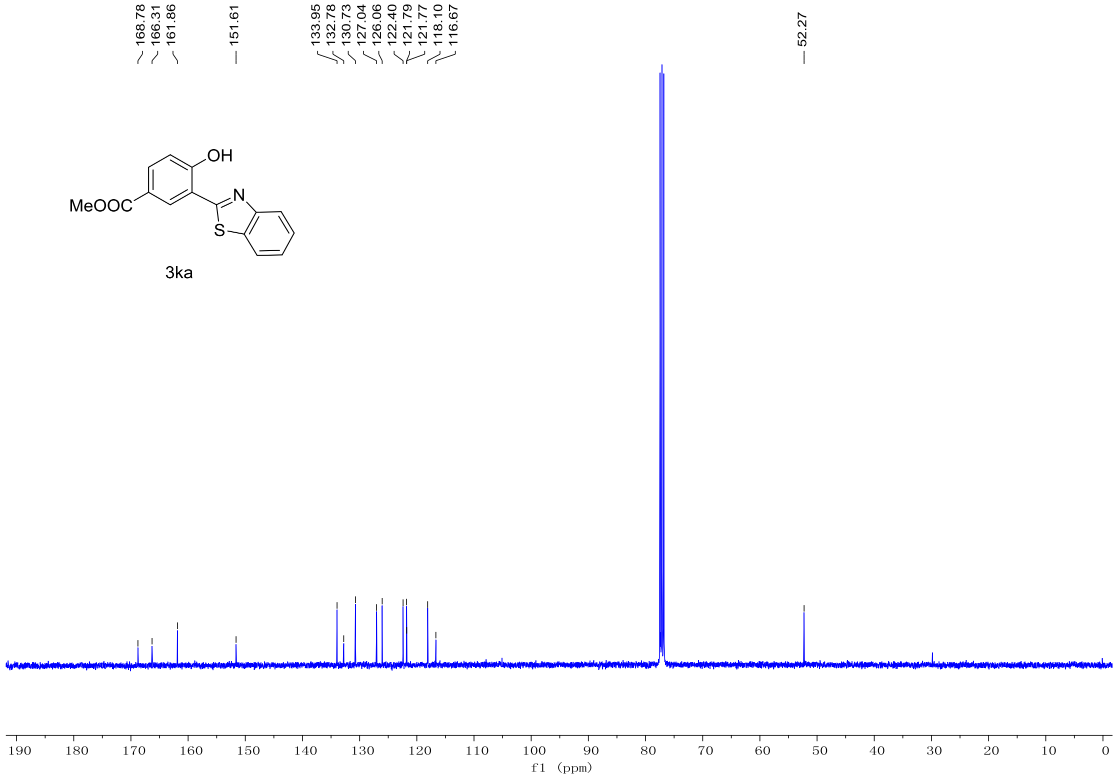
168.78
166.31
161.86
151.61
133.95
132.78
130.73
127.04
126.06
122.40
121.79
121.77
118.10
116.67
52.27
OH
MeOOC
N
S
3ka
f1 (ppm)

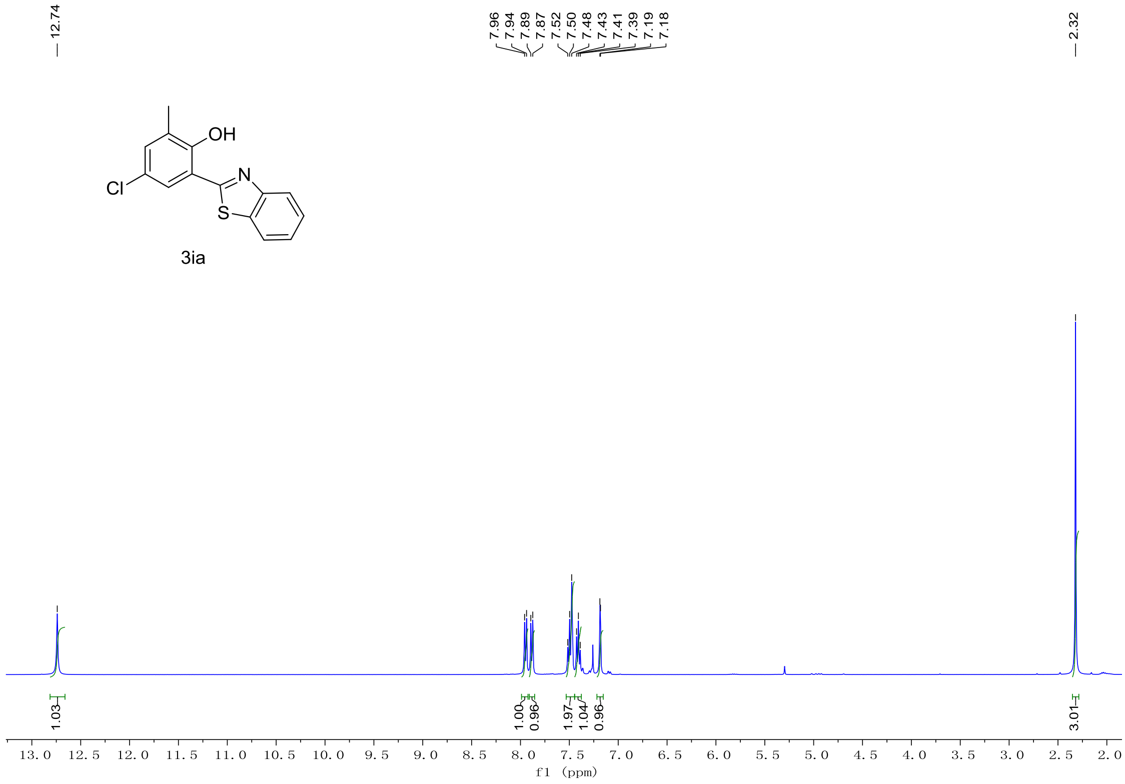
12.74
7.96
7.94
7.89
7.87
7.52
7.50
7.48
7.43
7.41
7.39
7.19
7.18
2.32
OH
N
S
Cl
3ia
1.03
1.00
0.96
1.97
1.04
0.96
3.01
f1 (ppm)

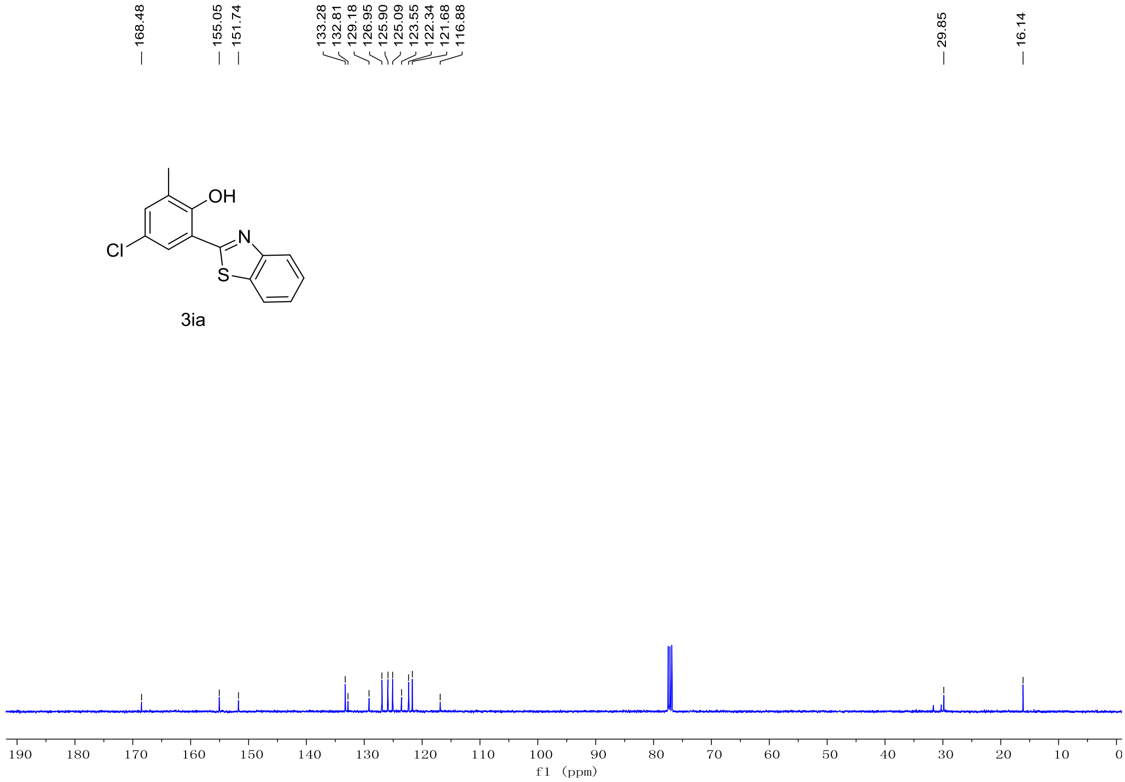
168.48
155.05
151.74
133.28
132.81
129.18
126.95
125.90
125.09
123.55
122.34
121.68
116.88
29.85
16.14
OH
N
S
Cl
3ia
f1 (ppm)

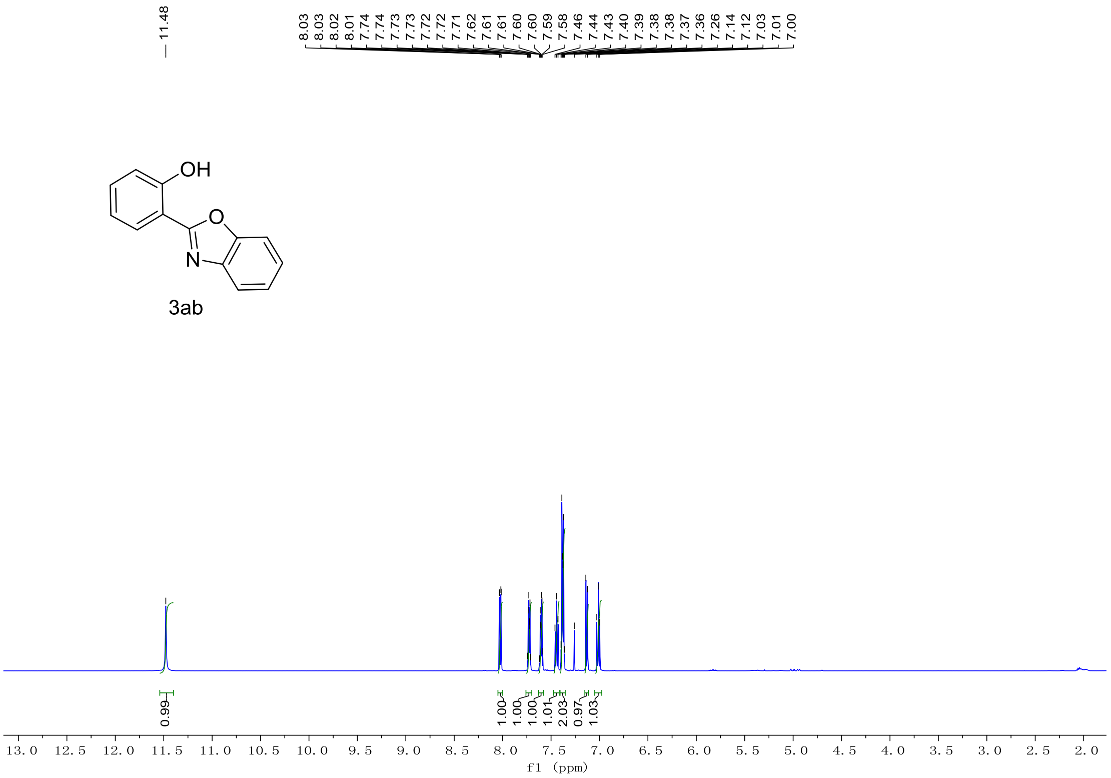
11.48
8.03
8.03
8.02
8.01
7.74
7.74
7.73
7.73
7.72
7.72
7.71
7.62
7.61
7.61
7.60
7.60
7.59
7.58
7.46
7.44
7.43
7.40
7.39
7.38
7.38
7.37
7.36
7.26
7.14
7.12
7.03
7.01
7.00
OH
O
N
3ab
0.99
1.00
1.00
1.00
1.01
2.03
0.97
1.03
13.0 12.5 12.0 11.5 11.0 10.5 10.0 9.5 9.0 8.5 8.0 7.5 7.0 6.5 6.0 5.5 5.0 4.5 4.0 3.5 3.0 2.5 2.0
f1 (ppm)

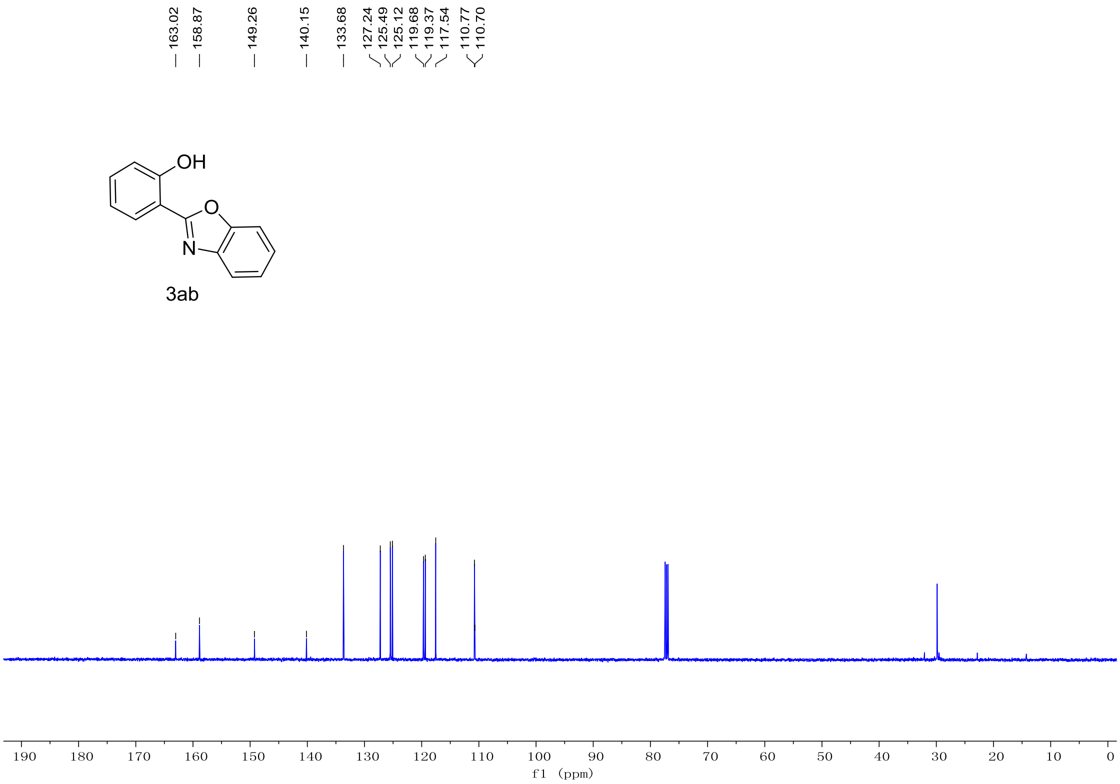
163.02
158.87
149.26
140.15
133.68
127.24
125.49
125.12
119.68
119.37
117.54
110.77
110.70
OH
O
N
3ab
190 180 170 160 150 140 130 120 110 100 90 80 70 60 50 40 30 20 10 0
f1 (ppm)

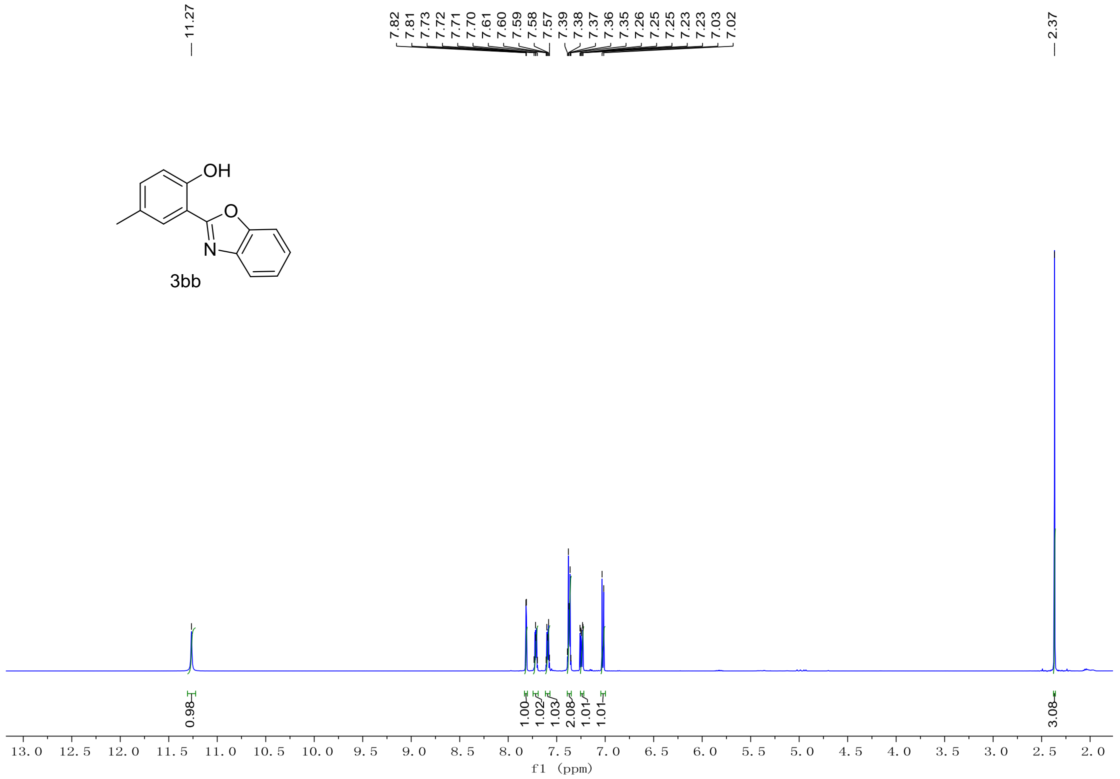
OH
O
N
11.27
7.82
7.81
7.73
7.72
7.71
7.70
7.61
7.60
7.59
7.58
7.57
7.39
7.38
7.37
7.36
7.35
7.26
7.25
7.25
7.23
7.23
7.03
7.02
2.37
0.98
1.00
1.02
1.03
2.08
1.01
1.01
3.08
f1 (ppm)

3bb

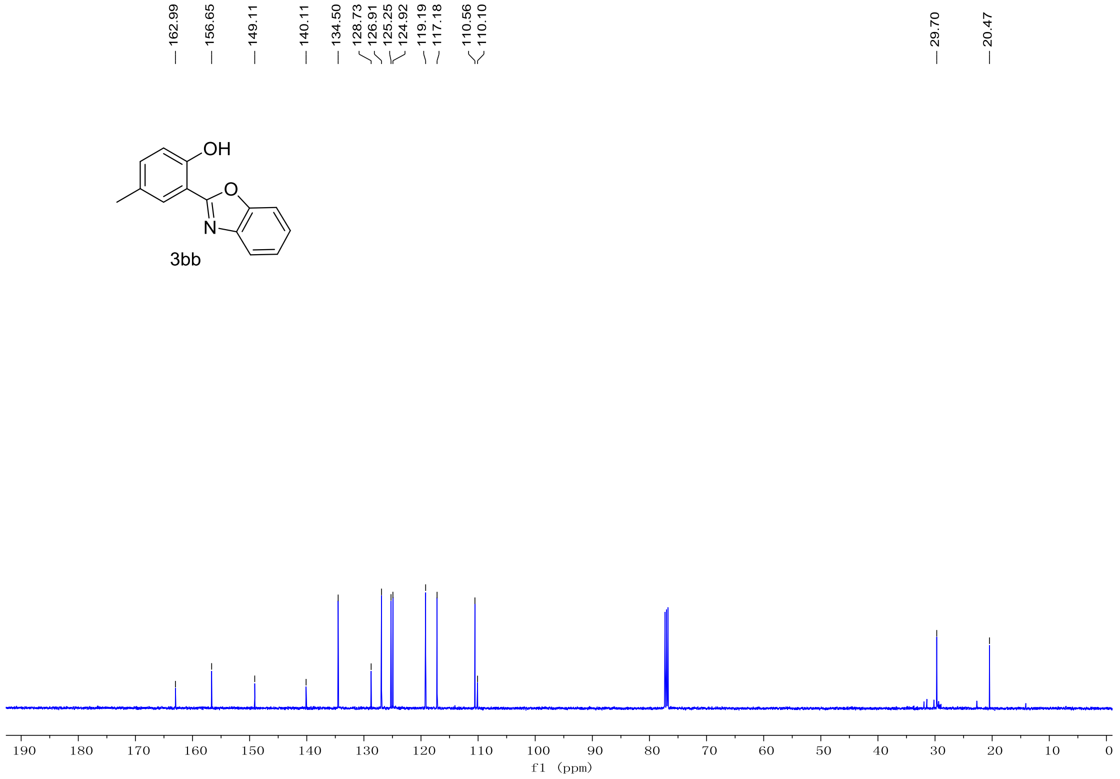
OH
O
N
162.99
156.65
149.11
140.11
134.50
128.73
126.91
125.25
124.92
119.19
117.18
110.56
110.10
29.70
20.47
f1 (ppm)

3bb

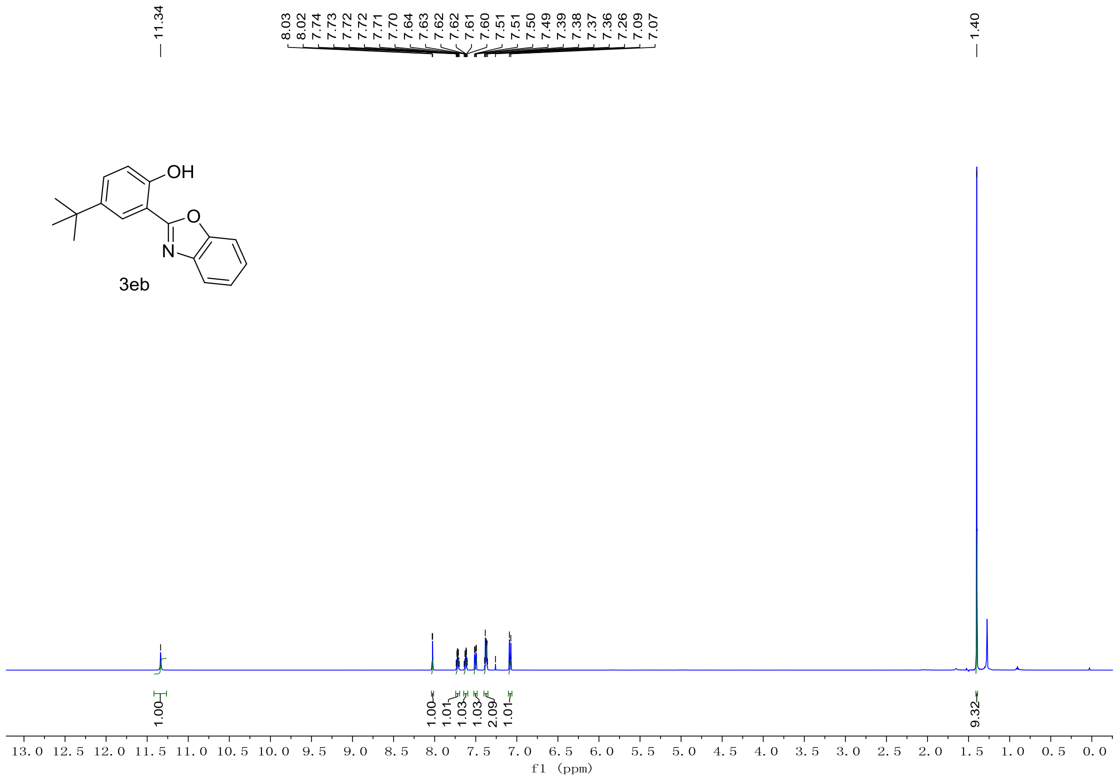
11.34
8.03 8.02 7.74 7.73 7.72 7.72 7.71 7.70 7.64 7.63 7.62 7.62 7.61 7.60 7.51 7.50 7.49 7.39 7.38 7.37 7.36 7.26 7.09 7.07
1.40
OH
O
N
3eb
1.00 1.00 1.01 1.03 1.03 2.09 1.01 9.32
13.0 12.5 12.0 11.5 11.0 10.5 10.0 9.5 9.0 8.5 8.0 7.5 7.0 6.5 6.0 5.5 5.0 4.5 4.0 3.5 3.0 2.5 2.0 1.5 1.0 0.5 0.0
f1 (ppm)

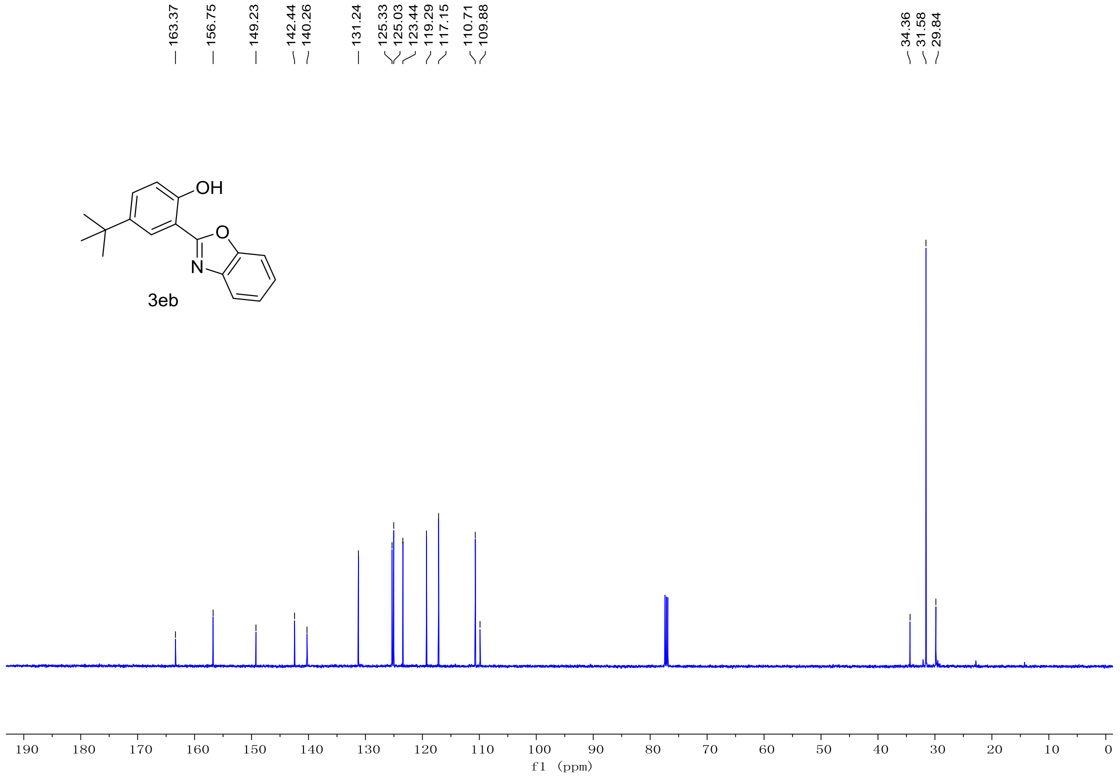
163.37 156.75 149.23 142.44 140.26 131.24 125.33 125.03 123.44 119.29 117.15 110.71 109.88
34.36 31.58 29.84
OH
O
N
3eb
190 180 170 160 150 140 130 120 110 100 90 80 70 60 50 40 30 20 10 0
f1 (ppm)

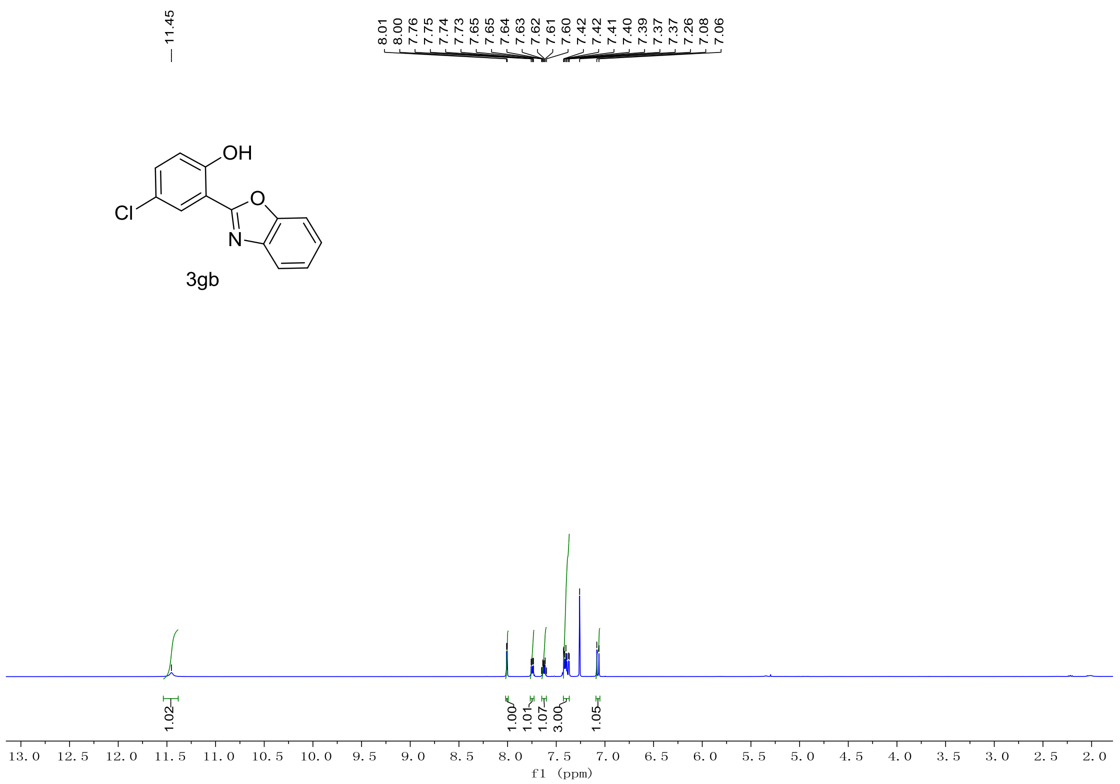
11.45
8.01 8.00 7.76 7.75 7.74 7.73 7.65 7.65 7.64 7.63 7.62 7.61 7.60 7.42 7.42 7.41 7.40 7.39 7.37 7.37 7.26 7.08 7.06
OH
Cl
O
N
3gb
1.02 1.00 1.01 1.07 3.00 1.05
13.0 12.5 12.0 11.5 11.0 10.5 10.0 9.5 9.0 8.5 8.0 7.5 7.0 6.5 6.0 5.5 5.0 4.5 4.0 3.5 3.0 2.5 2.0
f1 (ppm)

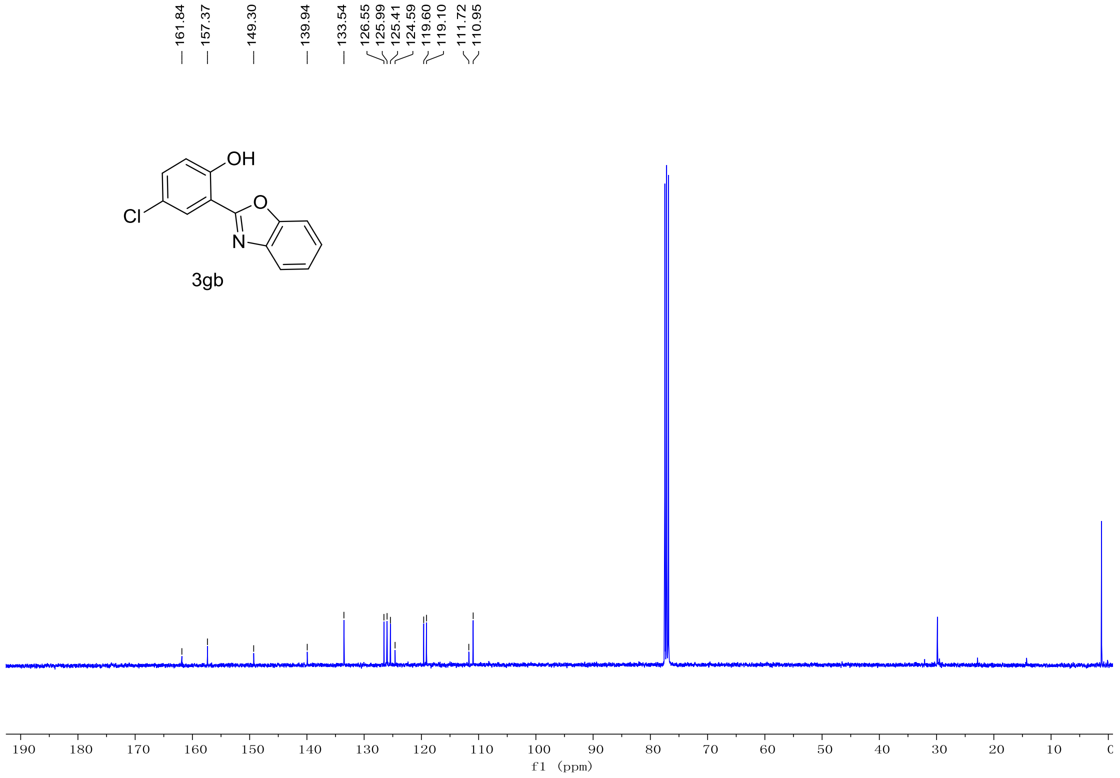
161.84 157.37 149.30 139.94 133.54 126.55 125.99 125.41 124.59 119.60 119.10 111.72 110.95
OH
Cl
O
N
3gb
190 180 170 160 150 140 130 120 110 100 90 80 70 60 50 40 30 20 10 0
f1 (ppm)

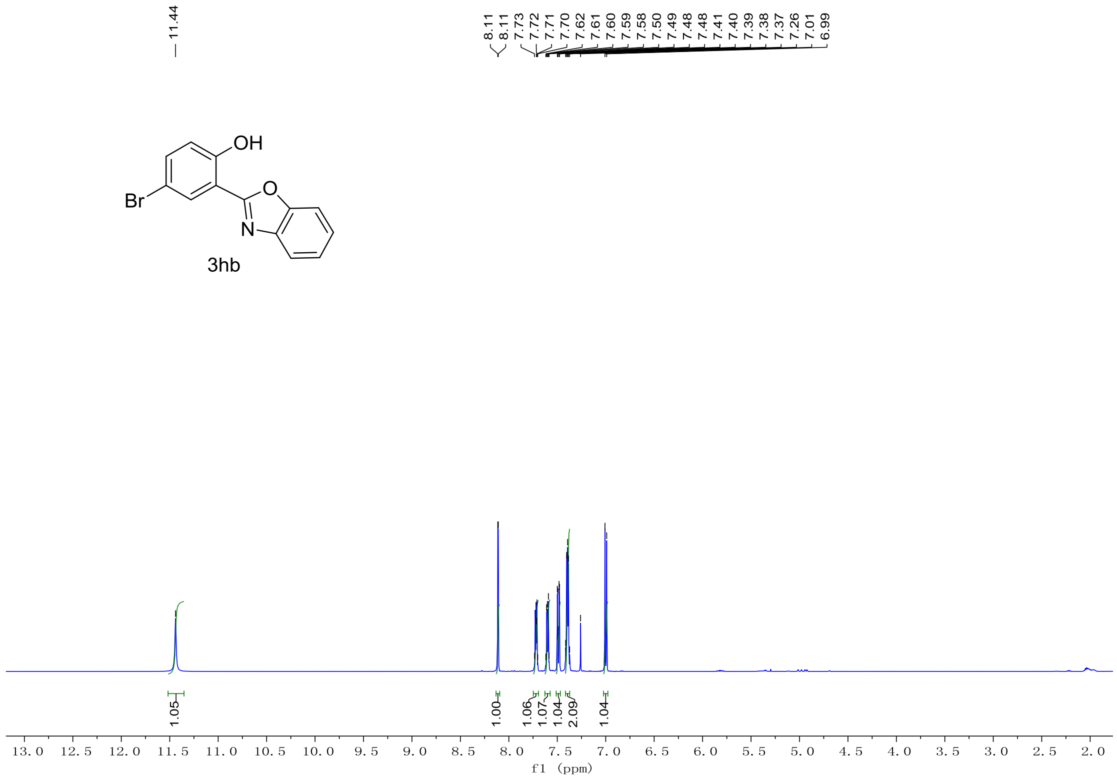

11.44
8.11
8.11
7.73
7.72
7.71
7.70
7.62
7.61
7.60
7.59
7.58
7.50
7.49
7.48
7.48
7.41
7.40
7.39
7.38
7.37
7.26
7.01
6.99
OH
Br
O
N
3hb
1.05
1.00
1.06
1.07
1.04
2.09
1.04
13.0 12.5 12.0 11.5 11.0 10.5 10.0 9.5 9.0 8.5 8.0 7.5 7.0 6.5 6.0 5.5 5.0 4.5 4.0 3.5 3.0 2.5 2.0
f1 (ppm)

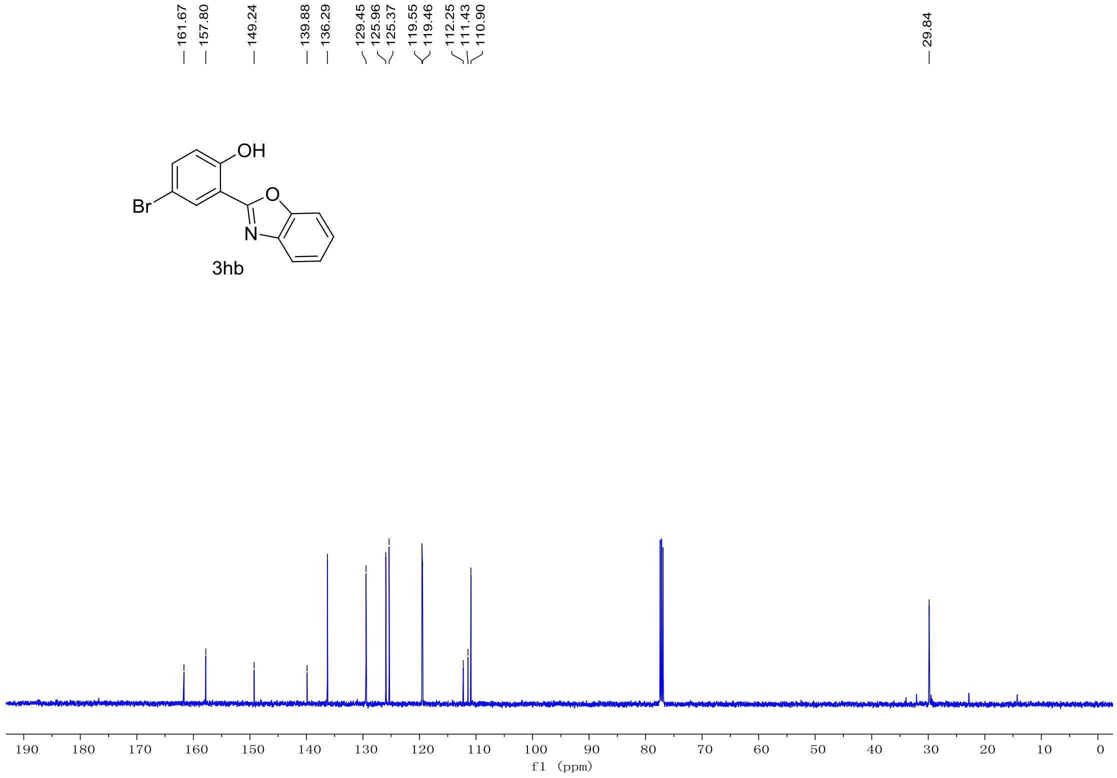

161.67
157.80
149.24
139.88
136.29
129.45
125.96
125.37
119.55
119.46
112.25
111.43
110.90
29.84
OH
Br
O
N
3hb
190 180 170 160 150 140 130 120 110 100 90 80 70 60 50 40 30 20 10 0
f1 (ppm)

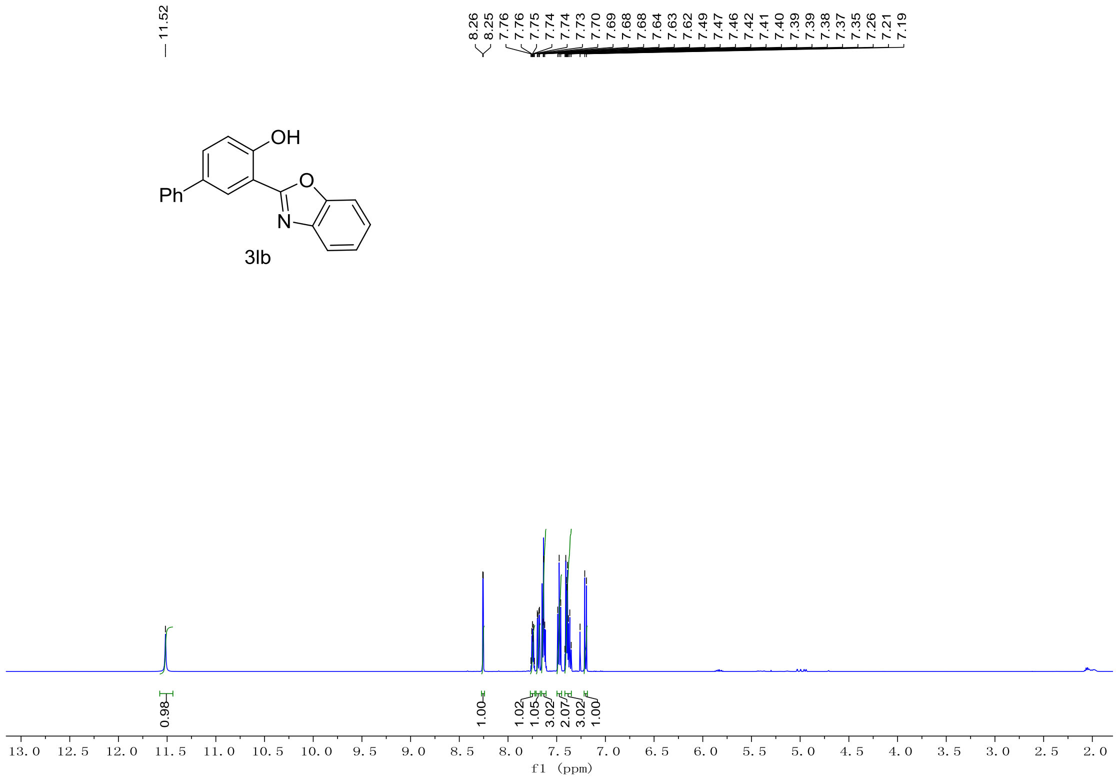
11.52
8.26
8.25
7.76
7.75
7.74
7.74
7.73
7.70
7.69
7.68
7.68
7.64
7.63
7.62
7.49
7.47
7.46
7.42
7.41
7.40
7.39
7.39
7.38
7.37
7.35
7.26
7.21
7.19
OH
Ph
O
N
3lb
0.98
1.00
1.02
1.05
3.02
2.07
3.02
1.00
13.0
12.5
12.0
11.5
11.0
10.5
10.0
9.5
9.0
8.5
8.0
7.5
7.0
6.5
6.0
5.5
5.0
4.5
4.0
3.5
3.0
2.5
2.0
f1 (ppm)

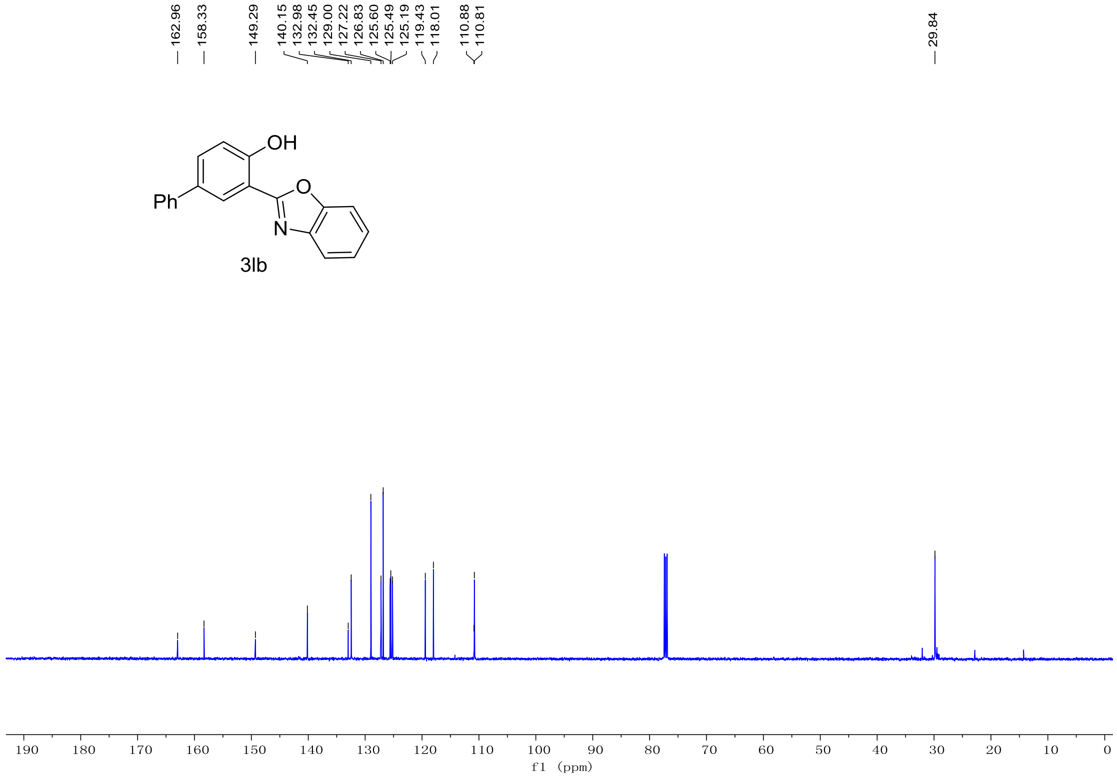
162.96
158.33
149.29
140.15
132.98
132.45
129.00
127.22
126.83
125.60
125.49
125.19
119.43
118.01
110.88
110.81
29.84
OH
Ph
O
N
3lb
190
180
170
160
150
140
130
120
110
100
90
80
70
60
50
40
30
20
10
0
f1 (ppm)

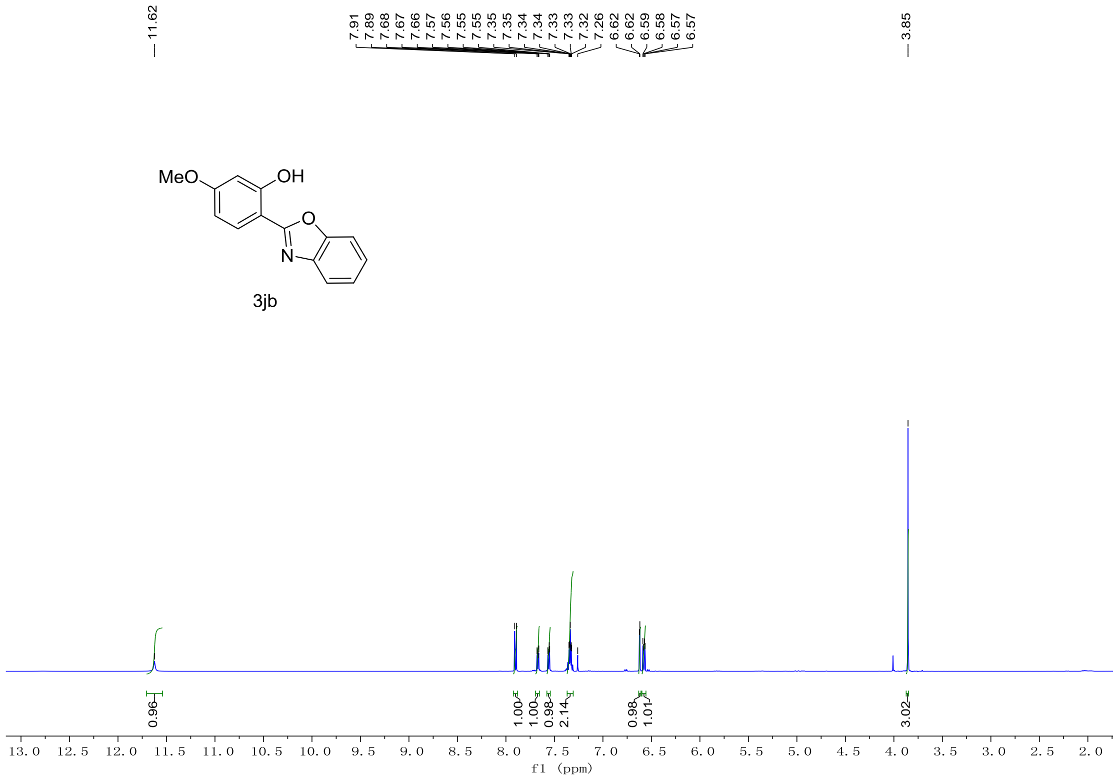

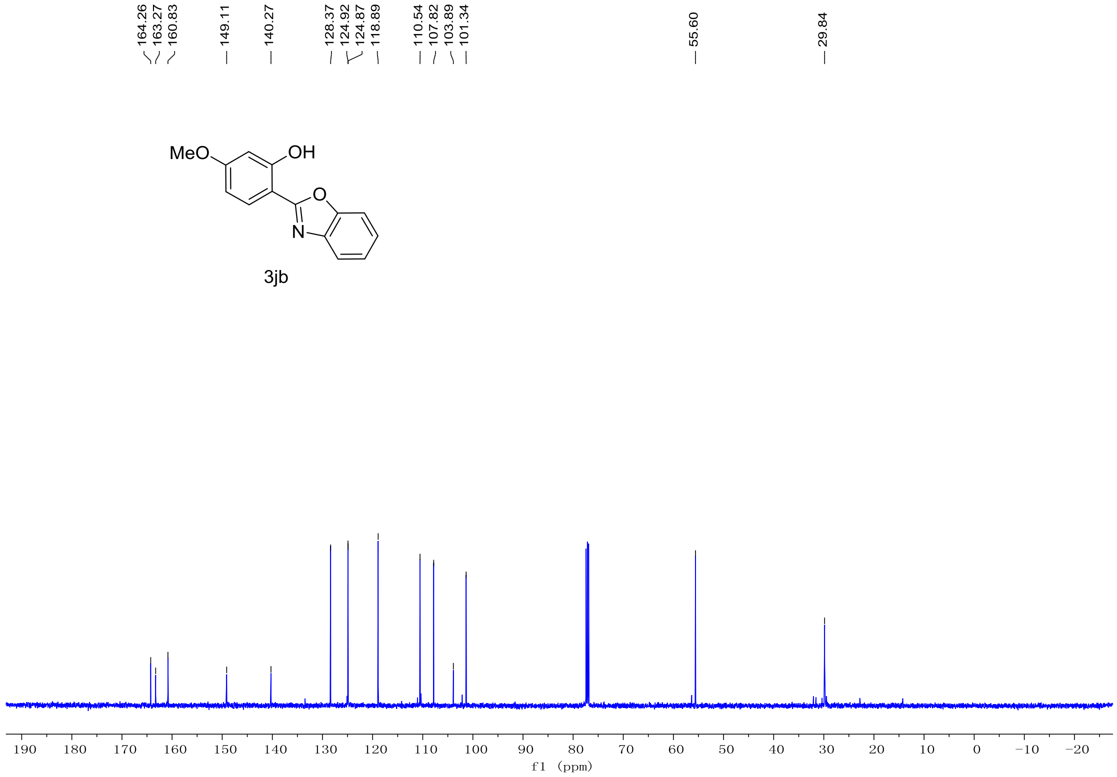

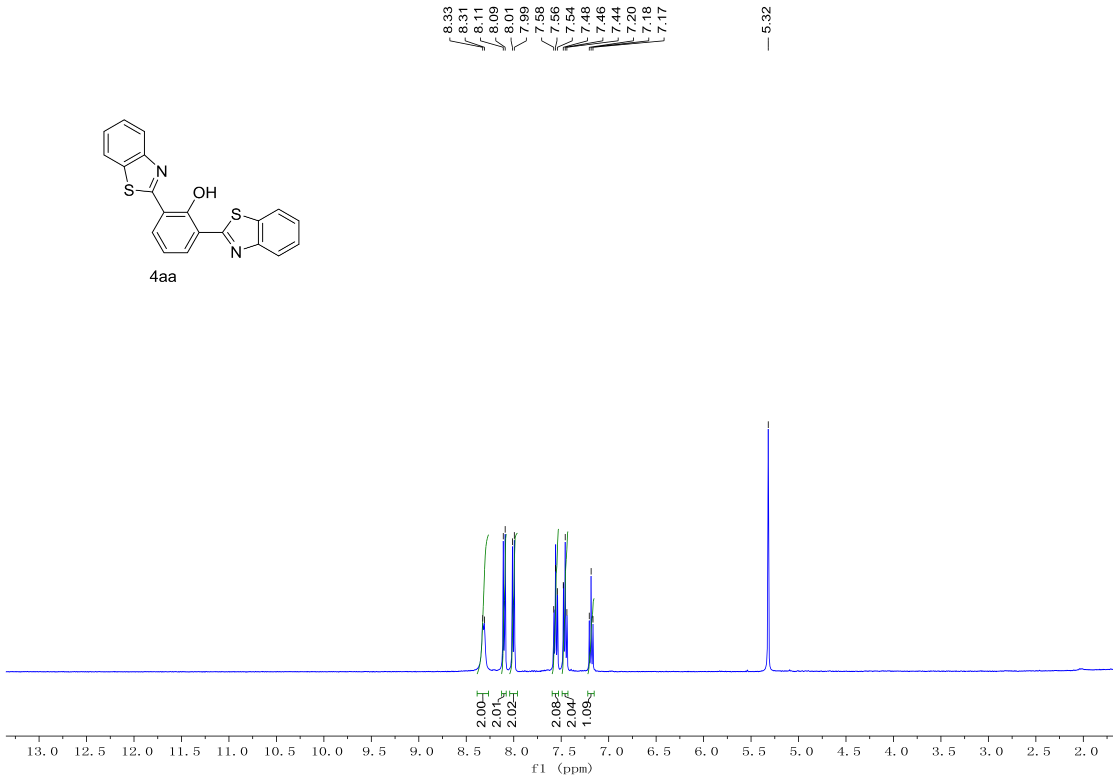
8.33
8.31
8.11
8.09
8.01
7.99
7.58
7.56
7.54
7.48
7.46
7.44
7.20
7.18
7.17
5.32
N
S
OH
S
N
2.00
2.01
2.02
2.08
2.04
1.09
13.0 12.5 12.0 11.5 11.0 10.5 10.0 9.5 9.0 8.5 8.0 7.5 7.0 6.5 6.0 5.5 5.0 4.5 4.0 3.5 3.0 2.5 2.0
f1 (ppm)

4aa

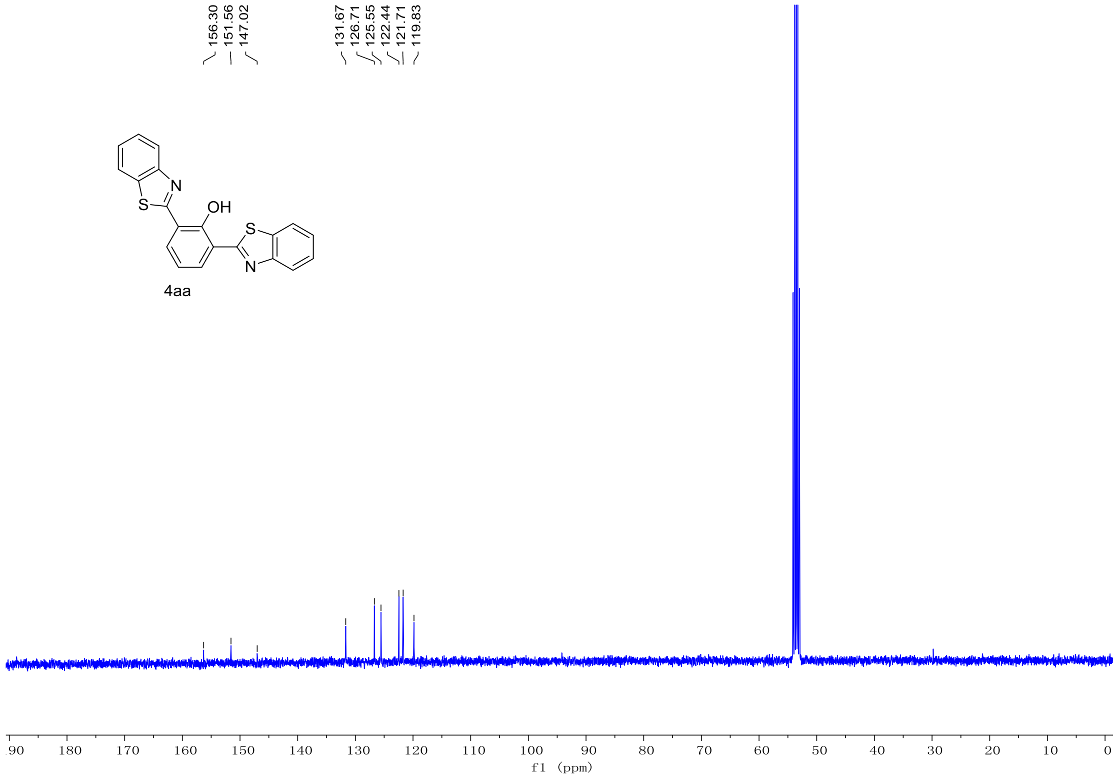
156.30
151.56
147.02
131.67
126.71
125.55
122.44
121.71
119.83
N
S
OH
S
N
190 180 170 160 150 140 130 120 110 100 90 80 70 60 50 40 30 20 10 0
f1 (ppm)

4aa

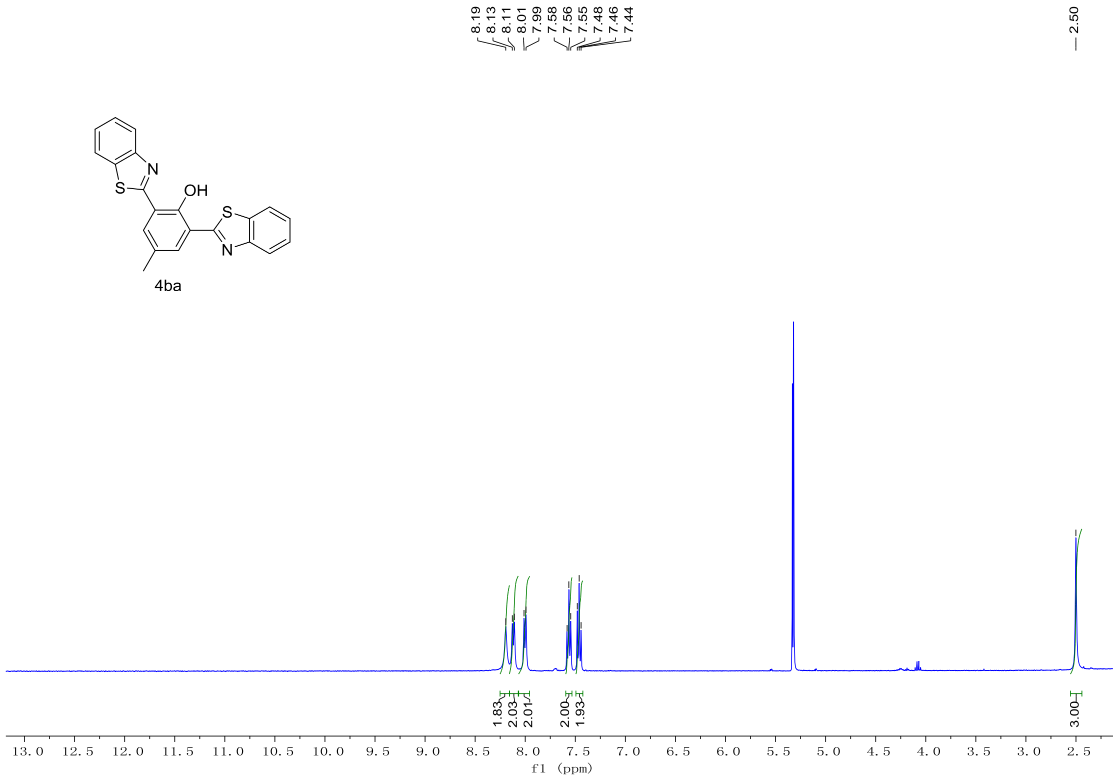

8.19
8.13
8.11
8.01
7.99
7.58
7.56
7.55
7.48
7.46
7.44
2.50
OH
S
N
4ba
1.83
2.03
2.01
2.00
1.93
3.00
13.0 12.5 12.0 11.5 11.0 10.5 10.0 9.5 9.0 8.5 8.0 7.5 7.0 6.5 6.0 5.5 5.0 4.5 4.0 3.5 3.0 2.5
f1 (ppm)

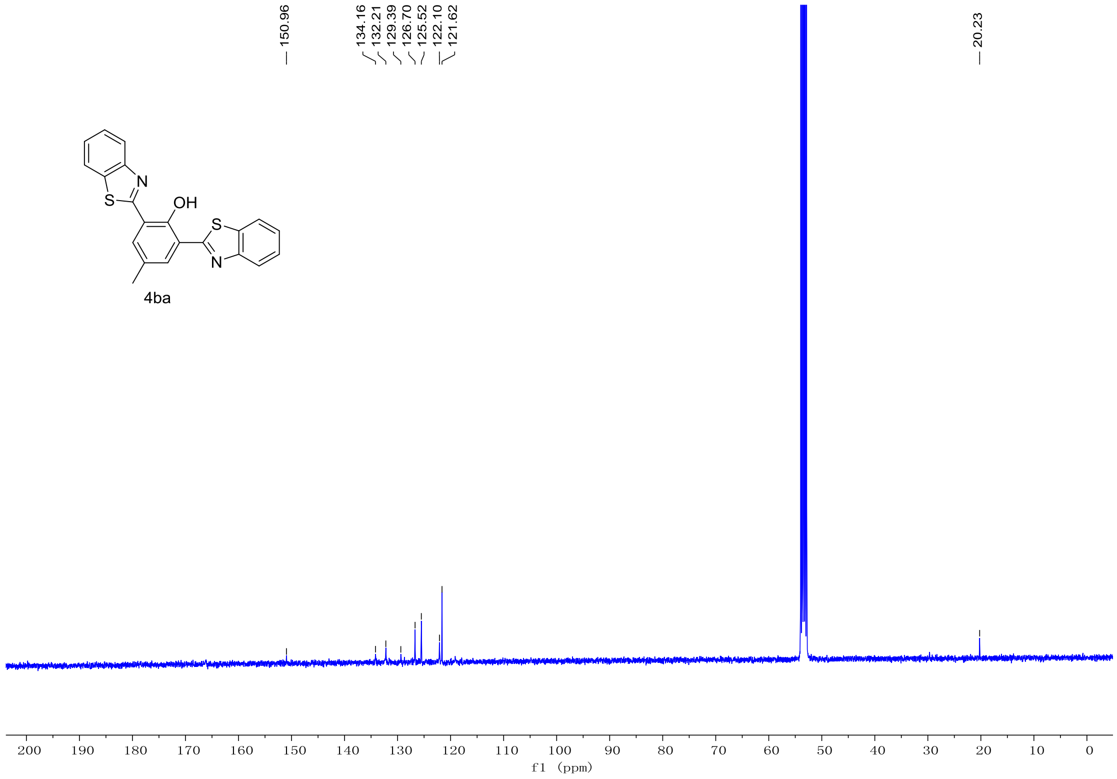

150.96
134.16
132.21
129.39
126.70
125.52
122.10
121.62
20.23
OH
S
N
4ba
200 190 180 170 160 150 140 130 120 110 100 90 80 70 60 50 40 30 20 10 0
f1 (ppm)

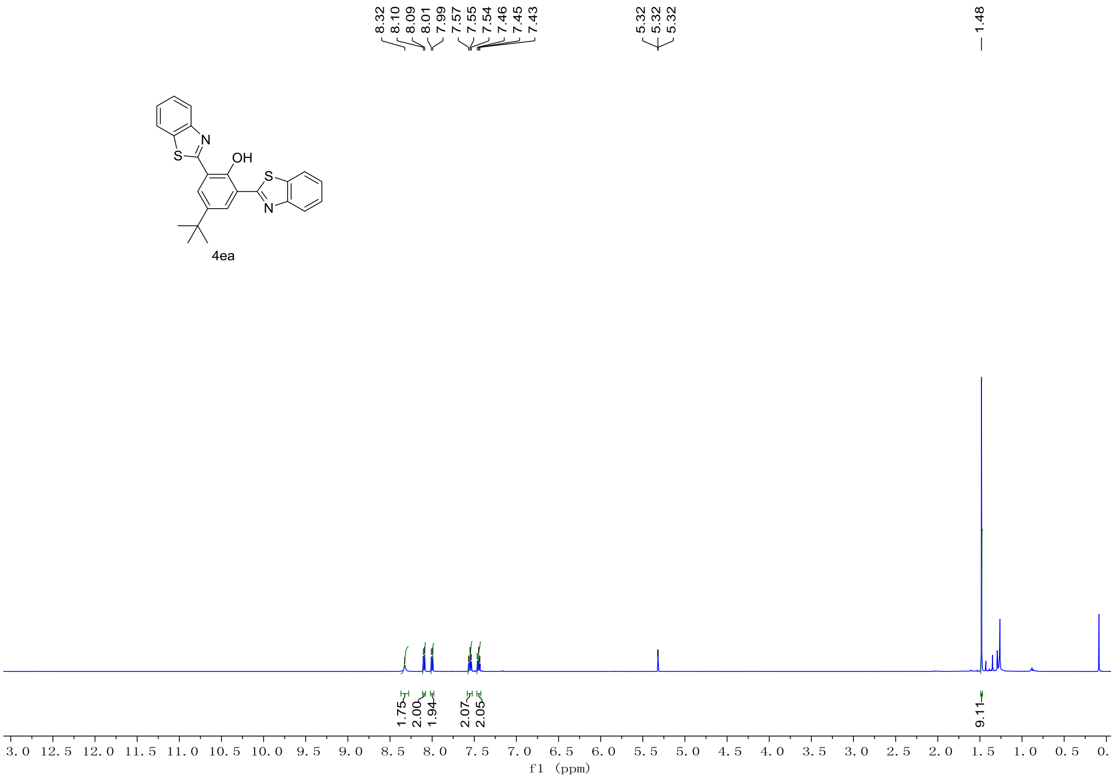
8.32
8.10
8.09
8.01
7.99
7.57
7.55
7.54
7.46
7.45
7.43
5.32
5.32
5.32
1.48
4ea
1.75
2.00
1.94
2.07
2.05
9.11
f1 (ppm)

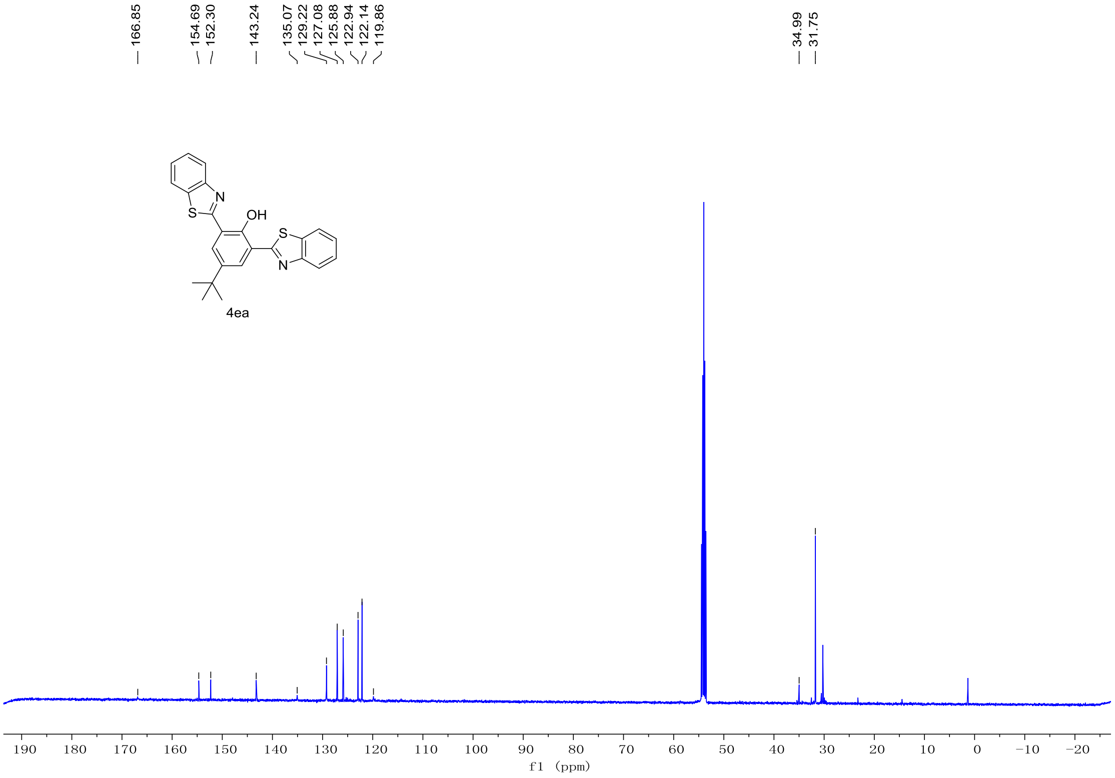
166.85
154.69
152.30
143.24
135.07
129.22
127.08
125.88
122.94
122.14
119.86
34.99
31.75
4ea
f1 (ppm)

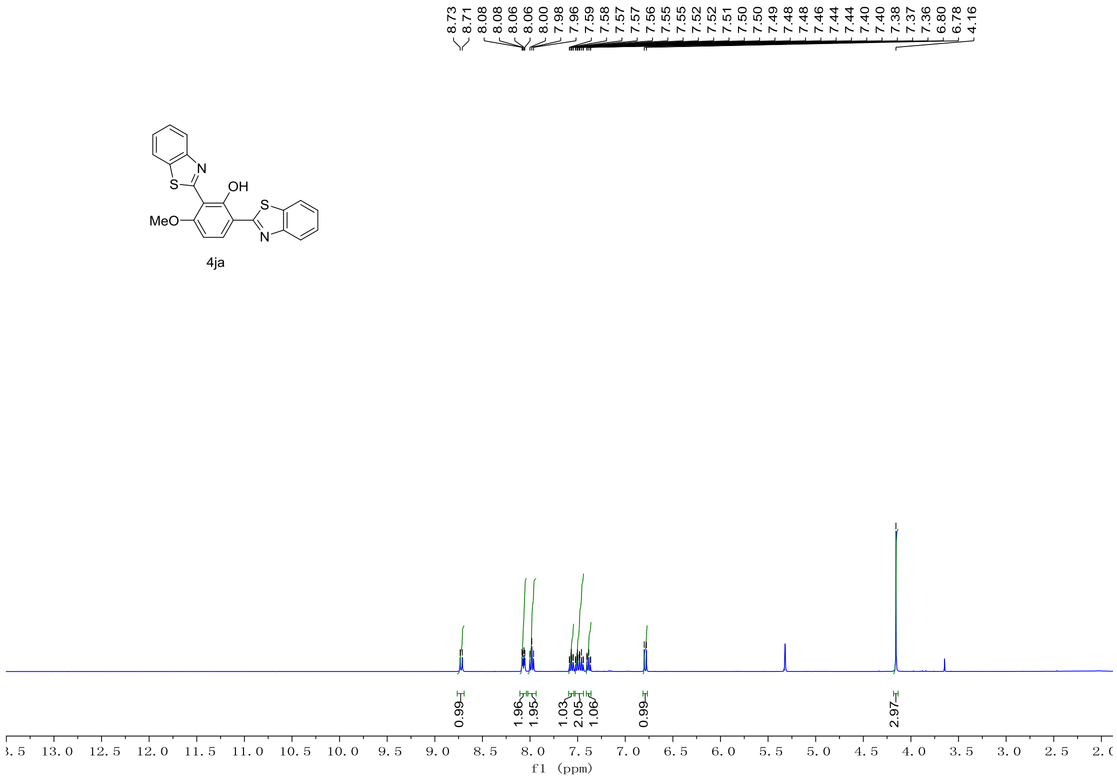
8.73
8.71
8.08
8.06
8.06
8.00
7.98
7.96
7.59
7.58
7.57
7.57
7.56
7.55
7.52
7.51
7.50
7.49
7.48
7.46
7.44
7.40
7.38
7.37
7.36
6.80
6.78
4.16
OH
MeO
N
S
4ja
0.99
1.96
1.95
1.03
2.05
1.06
0.99
2.97
13.0 12.5 12.0 11.5 11.0 10.5 10.0 9.5 9.0 8.5 8.0 7.5 7.0 6.5 6.0 5.5 5.0 4.5 4.0 3.5 3.0 2.5
f1 (ppm)

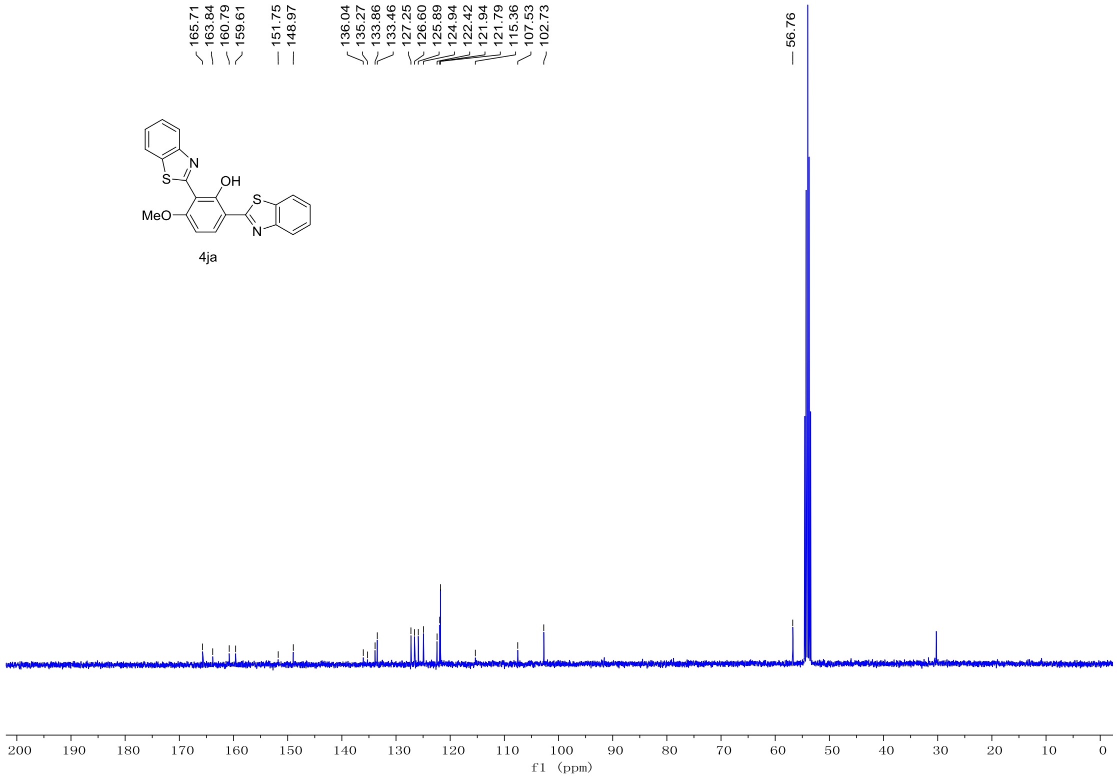
165.71
163.84
160.79
159.61
151.75
148.97
136.04
135.27
133.86
133.46
127.25
126.60
125.89
124.94
122.42
121.94
121.79
115.36
107.53
102.73
56.76
OH
MeO
N
S
4ja
200 190 180 170 160 150 140 130 120 110 100 90 80 70 60 50 40 30 20 10 0
f1 (ppm)

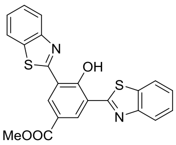
N
S
OH
S
N
MeOOC

4ka

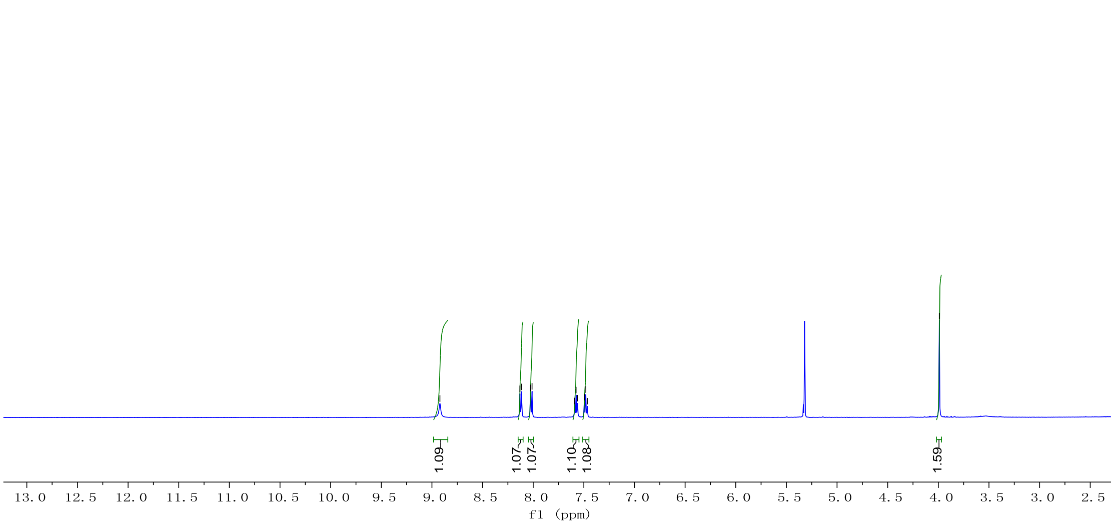
8.92
8.13
8.12
8.03
8.01
7.59
7.58
7.56
7.50
7.48
7.47
3.99
1.09
1.07
1.07
1.10
1.08
1.59
13.0 12.5 12.0 11.5 11.0 10.5 10.0 9.5 9.0 8.5 8.0 7.5 7.0 6.5 6.0 5.5 5.0 4.5 4.0 3.5 3.0 2.5
f1 (ppm)

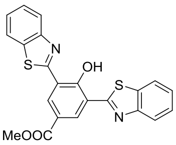
N
S
OH
S
N
MeOOC

4ka

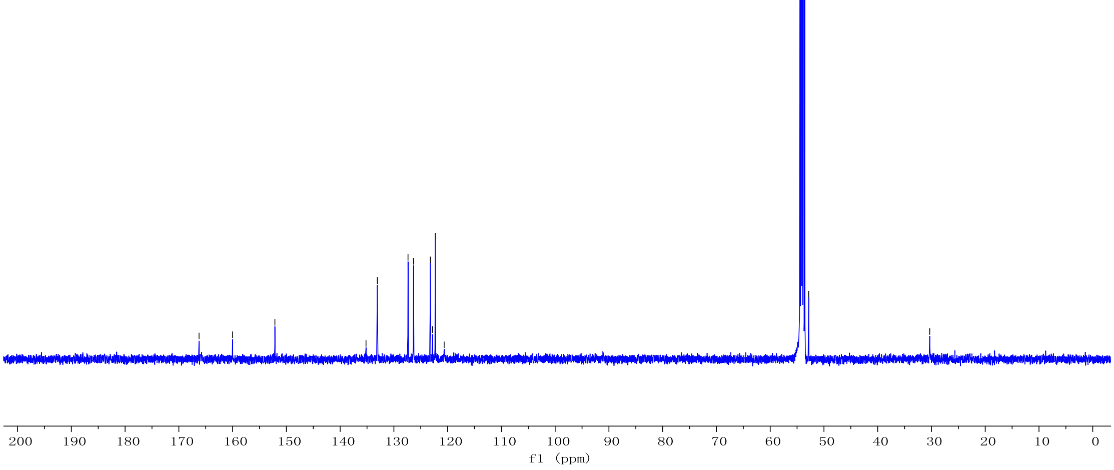
166.25
160.00
152.13
135.19
133.08
127.37
126.35
123.22
122.81
122.30
120.65
52.83
30.32
200 190 180 170 160 150 140 130 120 110 100 90 80 70 60 50 40 30 20 10 0
f1 (ppm)

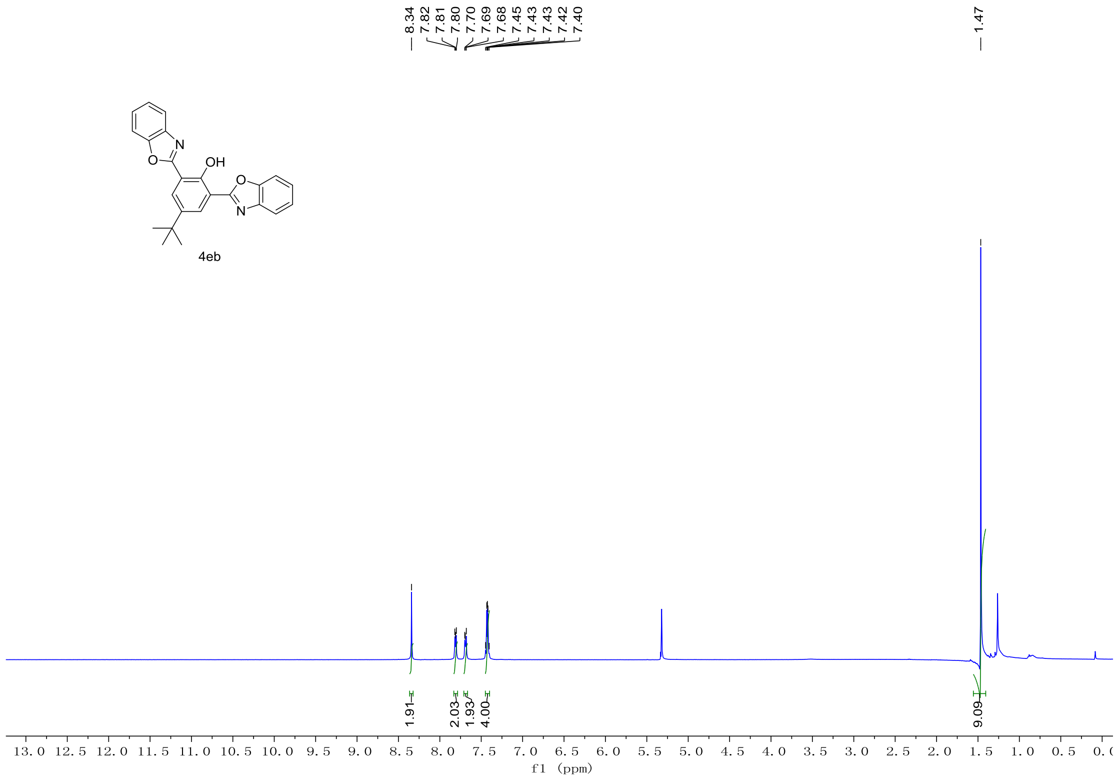
8.34
7.82
7.81
7.80
7.70
7.69
7.68
7.45
7.43
7.43
7.42
7.40
1.47
OH
O
N
4eb
1.91
2.03
1.93
4.00
9.09
13.0 12.5 12.0 11.5 11.0 10.5 10.0 9.5 9.0 8.5 8.0 7.5 7.0 6.5 6.0 5.5 5.0 4.5 4.0 3.5 3.0 2.5 2.0 1.5 1.0 0.5
f1 (ppm)

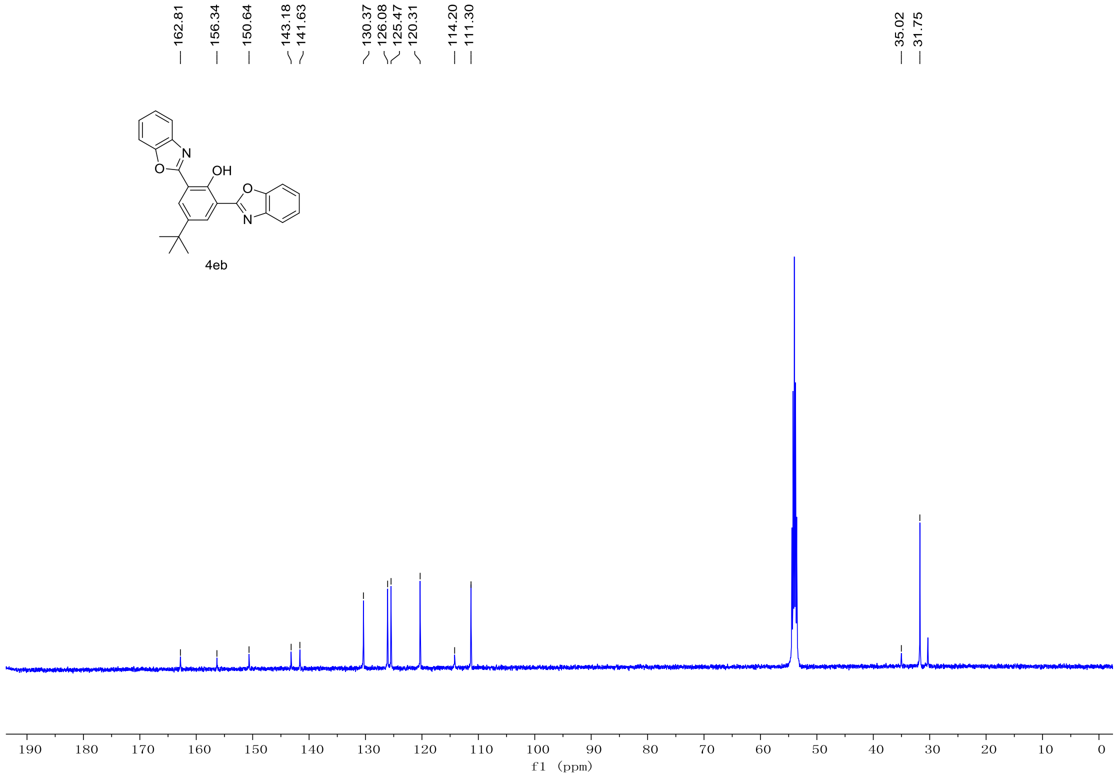
162.81
156.34
150.64
143.18
141.63
130.37
126.08
125.47
120.31
114.20
111.30
35.02
31.75
OH
O
N
4eb
190 180 170 160 150 140 130 120 110 100 90 80 70 60 50 40 30 20 10 0
f1 (ppm)

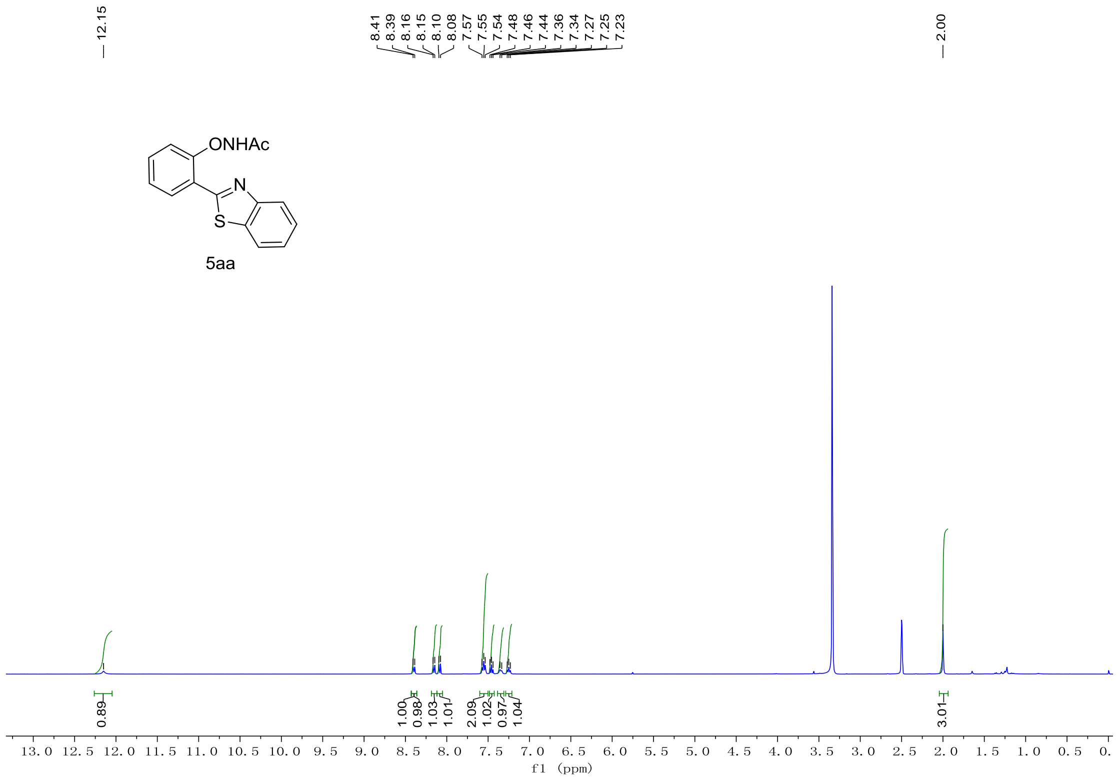
12.15
8.41
8.39
8.16
8.15
8.10
8.08
7.57
7.55
7.54
7.48
7.46
7.44
7.36
7.34
7.27
7.25
7.23
2.00
ONHAc
N
S
5aa
0.89
1.00
0.98
1.03
1.01
2.09
1.02
0.97
1.04
3.01
f1 (ppm)

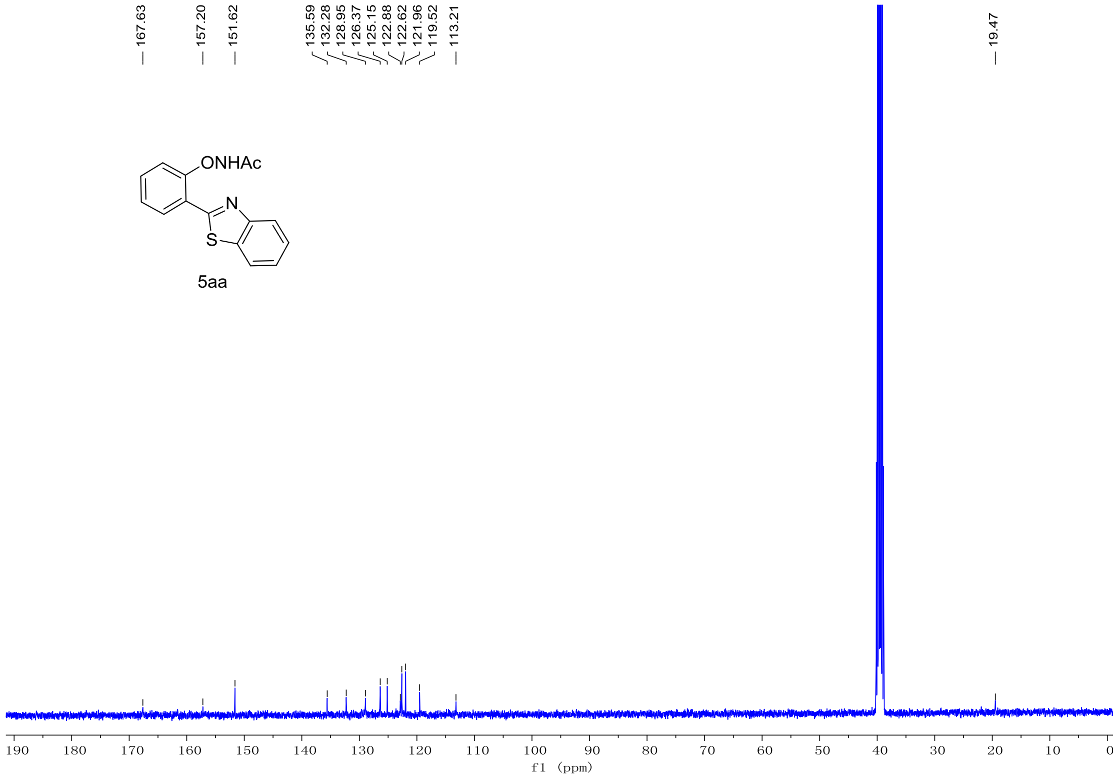
167.63
157.20
151.62
135.59
132.28
128.95
126.37
125.15
122.88
122.62
121.96
119.52
113.21
19.47
ONHAc
N
S
5aa
f1 (ppm)

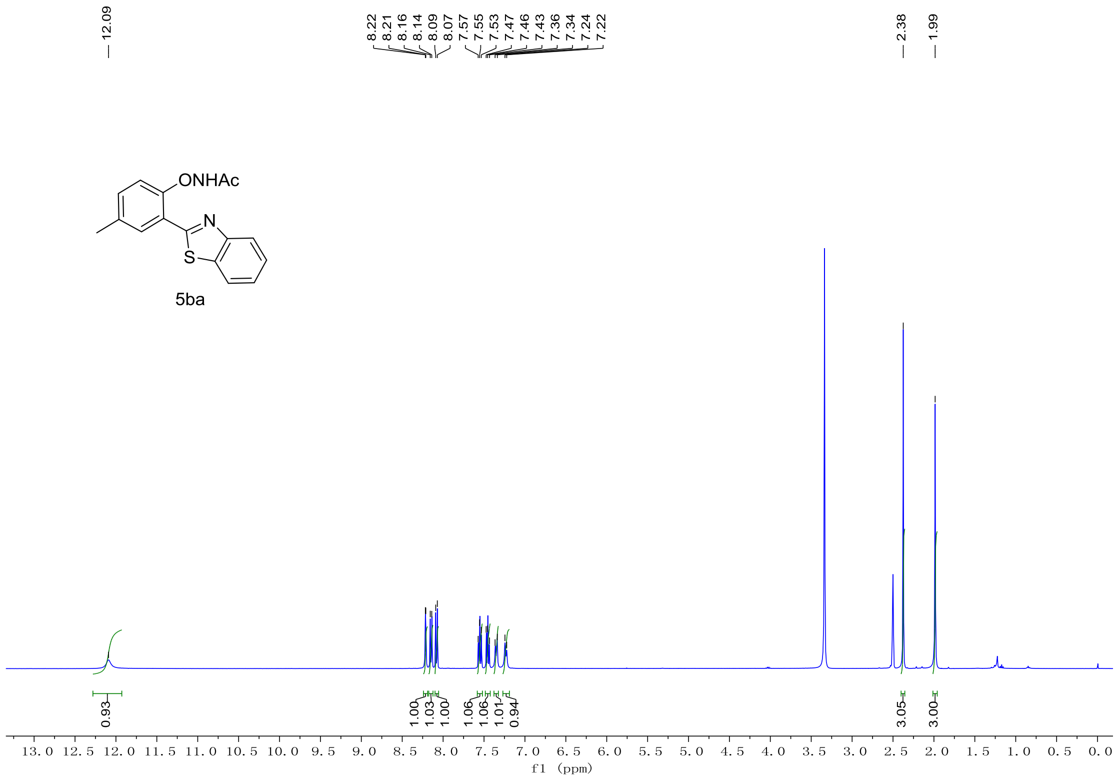
12.09
8.22
8.21
8.16
8.14
8.09
8.07
7.57
7.55
7.53
7.47
7.46
7.43
7.36
7.34
7.24
7.22
2.38
1.99
ONHAc
N
S
5ba
0.93
1.00
1.03
1.00
1.06
1.06
1.01
0.94
3.05
3.00
f1 (ppm)

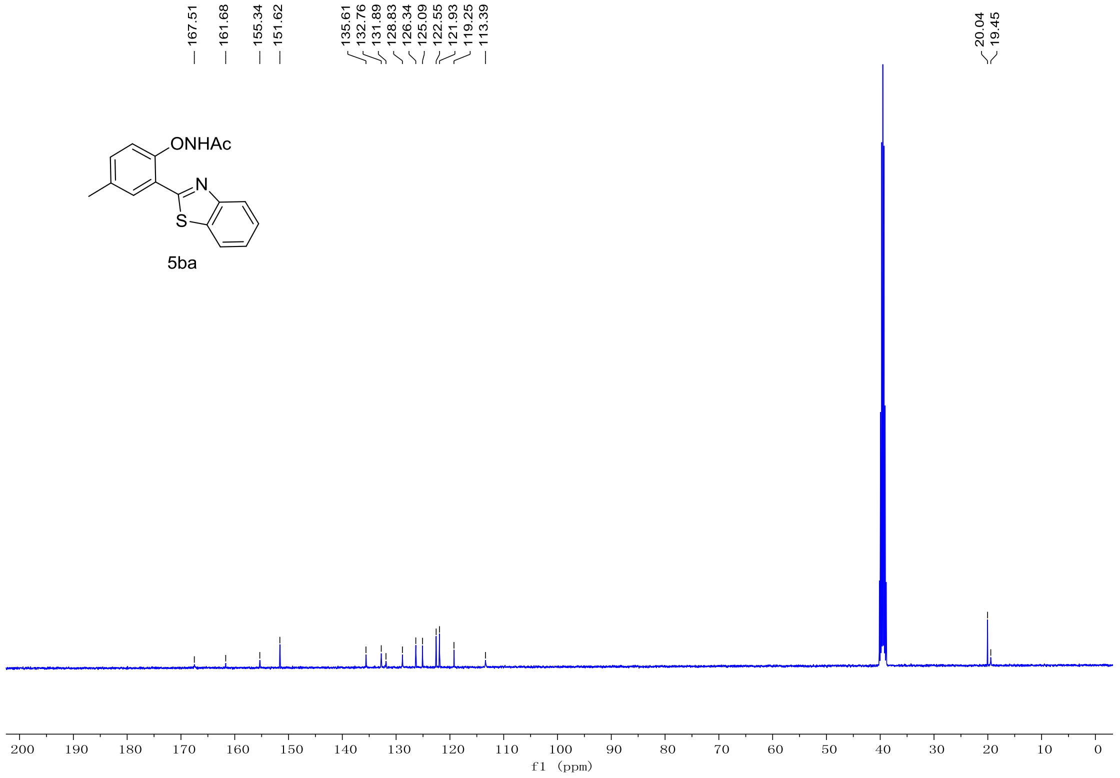
167.51
161.68
155.34
151.62
135.61
132.76
131.89
128.83
126.34
125.09
122.55
121.93
119.25
113.39
20.04
19.45
ONHAc
N
S
5ba
f1 (ppm)

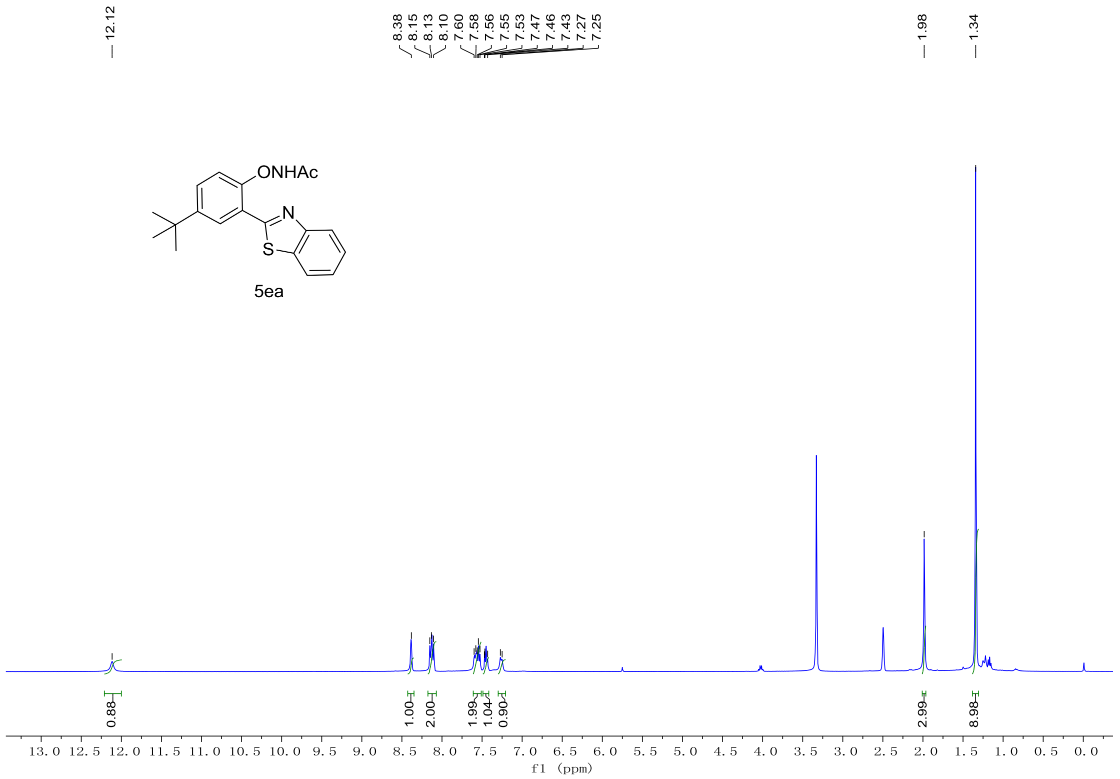
12.12
8.38
8.15
8.13
8.10
7.60
7.58
7.56
7.55
7.53
7.47
7.46
7.43
7.27
7.25
1.98
1.34
ONHAc
N
S
5ea
0.88
1.00
2.00
1.99
1.04
0.90
2.99
8.98
13.0 12.5 12.0 11.5 11.0 10.5 10.0 9.5 9.0 8.5 8.0 7.5 7.0 6.5 6.0 5.5 5.0 4.5 4.0 3.5 3.0 2.5 2.0 1.5 1.0 0.5 0.0
f1 (ppm)

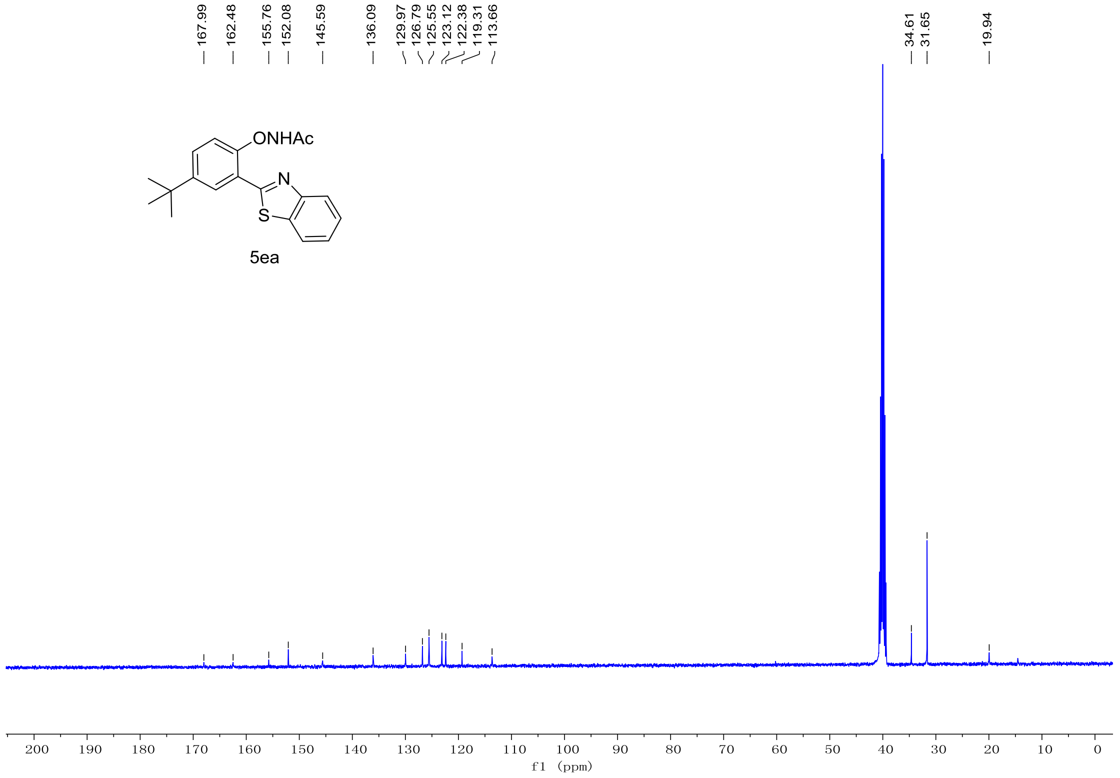
167.99
162.48
155.76
152.08
145.59
136.09
129.97
126.79
125.55
123.12
122.38
119.31
113.66
34.61
31.65
19.94
ONHAc
N
S
5ea
200 190 180 170 160 150 140 130 120 110 100 90 80 70 60 50 40 30 20 10 0
f1 (ppm)

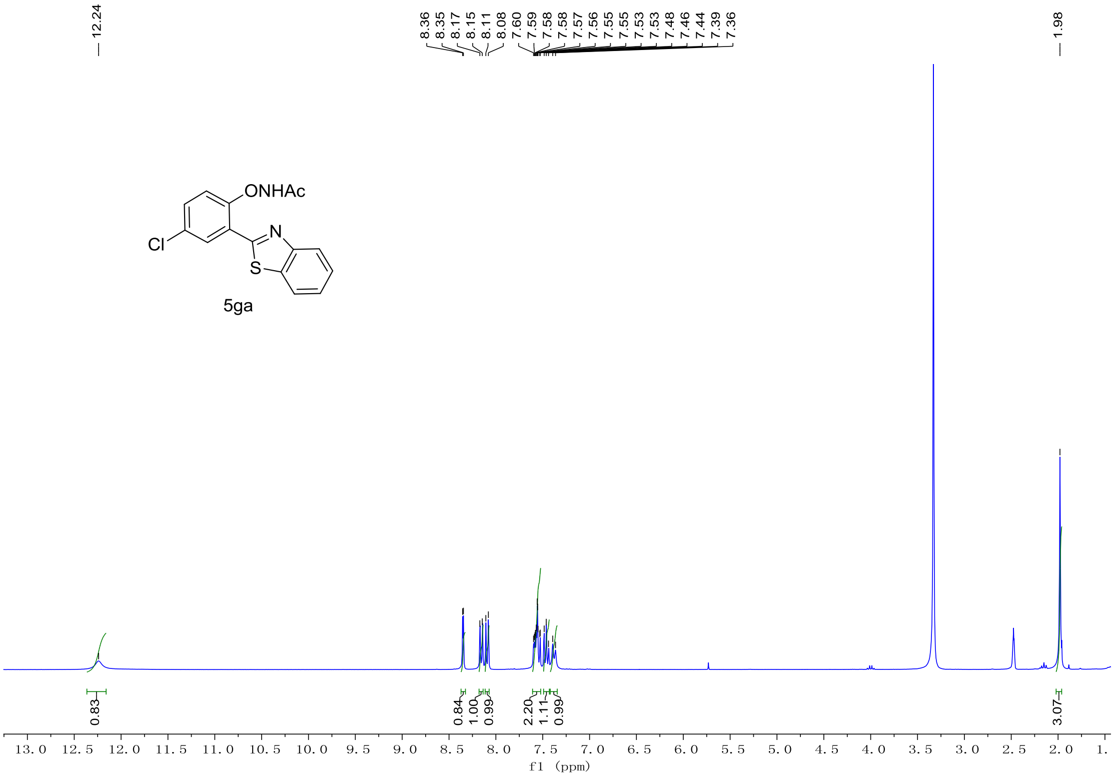
12.24
8.36
8.35
8.17
8.15
8.11
8.08
7.60
7.59
7.58
7.58
7.57
7.56
7.55
7.55
7.53
7.53
7.48
7.46
7.44
7.39
7.36
1.98
ONHAc
Cl
N
S
5ga
0.83
0.84
1.00
0.99
2.20
1.11
0.99
3.07
13.0 12.5 12.0 11.5 11.0 10.5 10.0 9.5 9.0 8.5 8.0 7.5 7.0 6.5 6.0 5.5 5.0 4.5 4.0 3.5 3.0 2.5 2.0 1.
f1 (ppm)

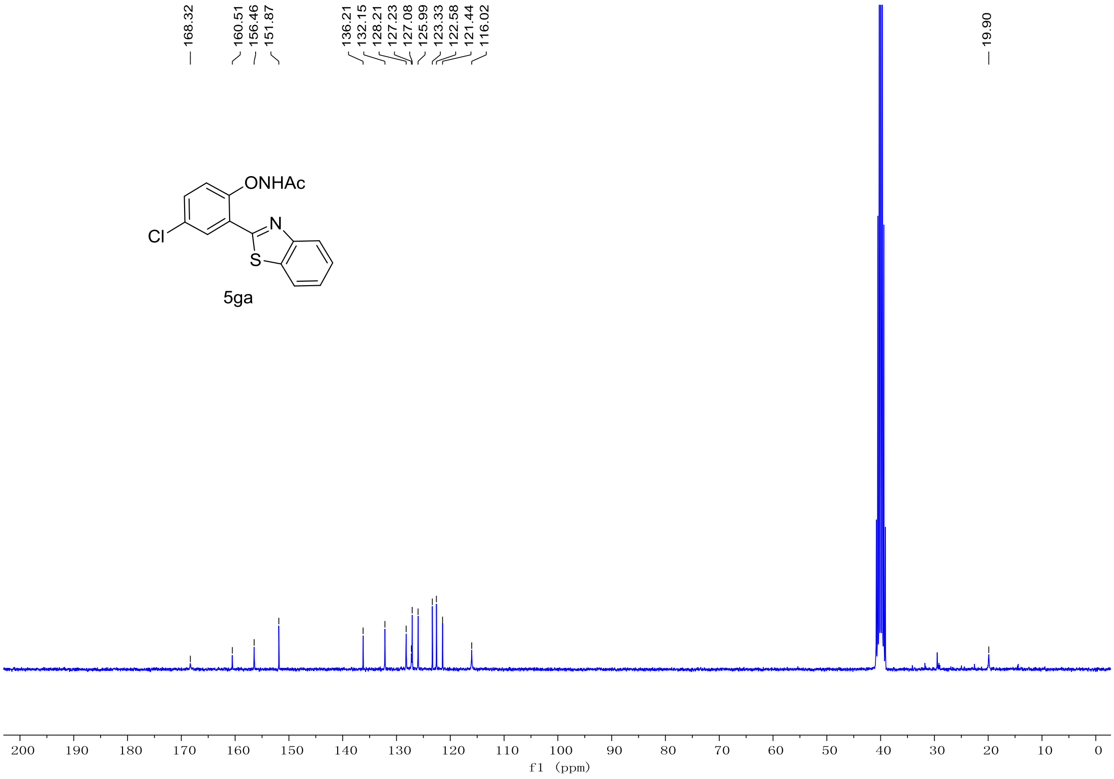
168.32
160.51
156.46
151.87
136.21
132.15
128.21
127.23
127.08
125.99
123.33
122.58
121.44
116.02
19.90
ONHAc
Cl
N
S
5ga
200 190 180 170 160 150 140 130 120 110 100 90 80 70 60 50 40 30 20 10 0
f1 (ppm)

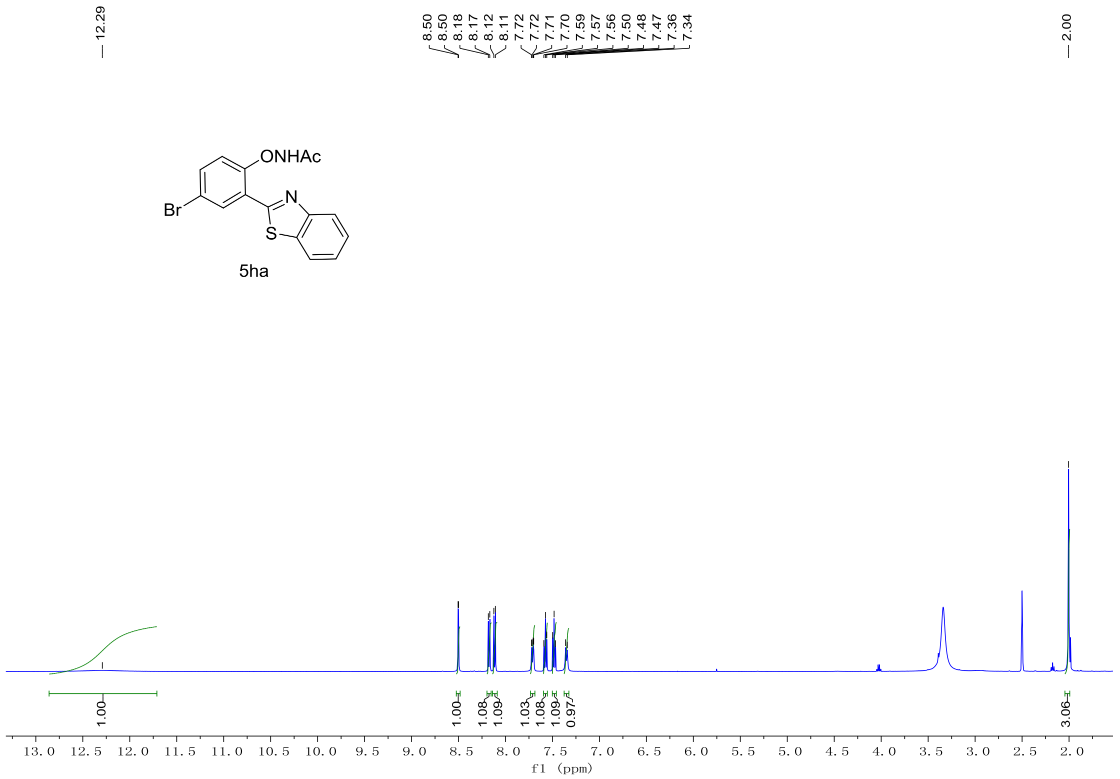
12.29
8.50
8.50
8.18
8.17
8.12
8.11
7.72
7.72
7.71
7.70
7.59
7.57
7.56
7.50
7.48
7.47
7.36
7.34
2.00
ONHAc
Br
N
S
5ha
1.00
1.00
1.08
1.09
1.03
1.08
1.09
0.97
3.06
f1 (ppm)

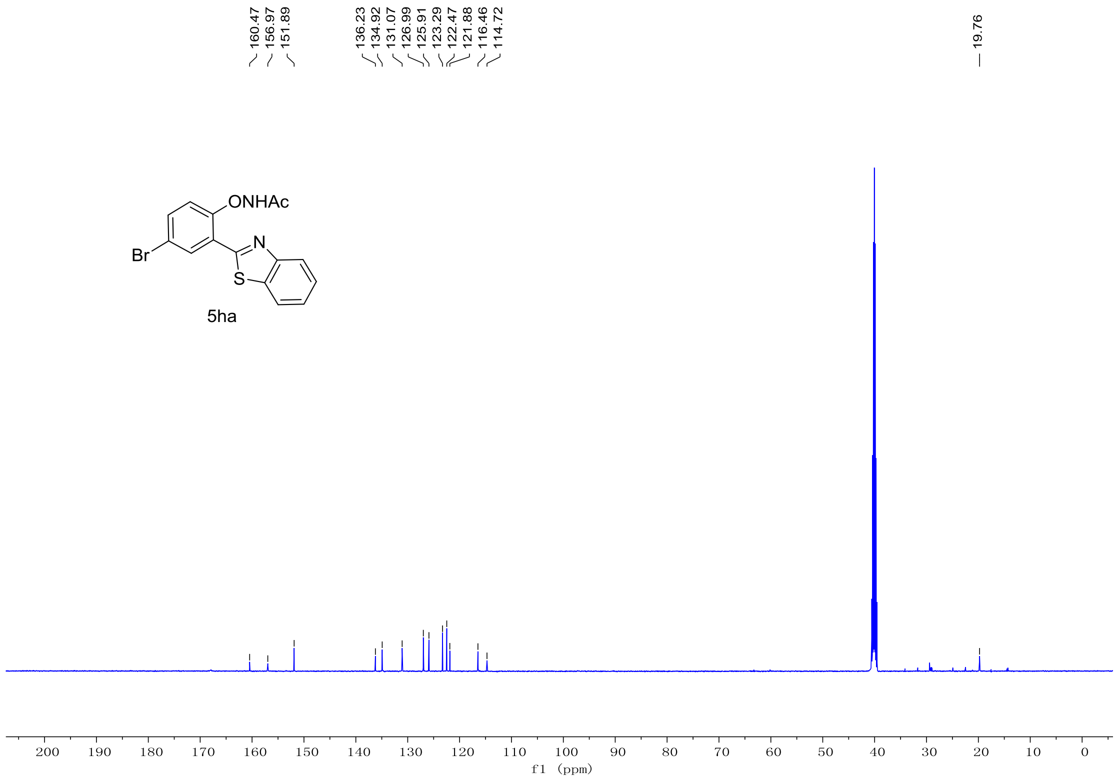
160.47
156.97
151.89
136.23
134.92
131.07
126.99
125.91
123.29
122.47
121.88
116.46
114.72
19.76
ONHAc
Br
N
S
5ha
f1 (ppm)

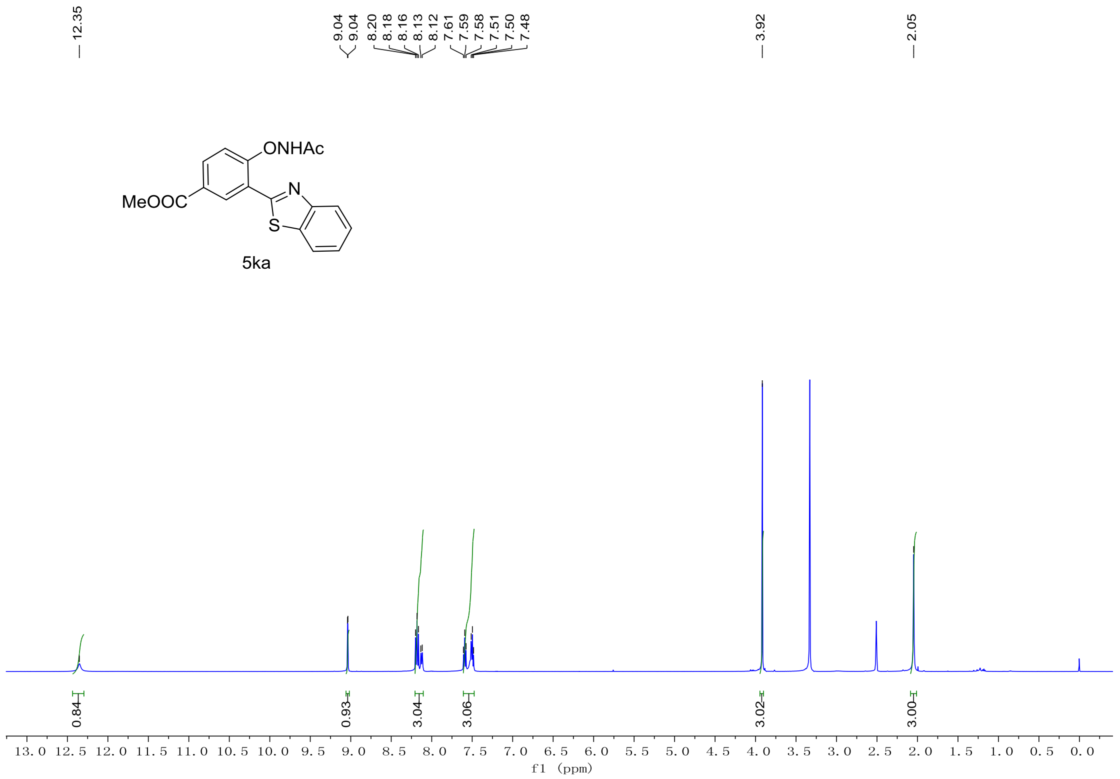
12.35
9.04
9.04
8.20
8.18
8.16
8.13
8.12
7.61
7.59
7.58
7.51
7.50
7.48
3.92
2.05
ONHAc
MeOOC
N
S
5ka
0.84
0.93
3.04
3.06
3.02
3.00
f1 (ppm)

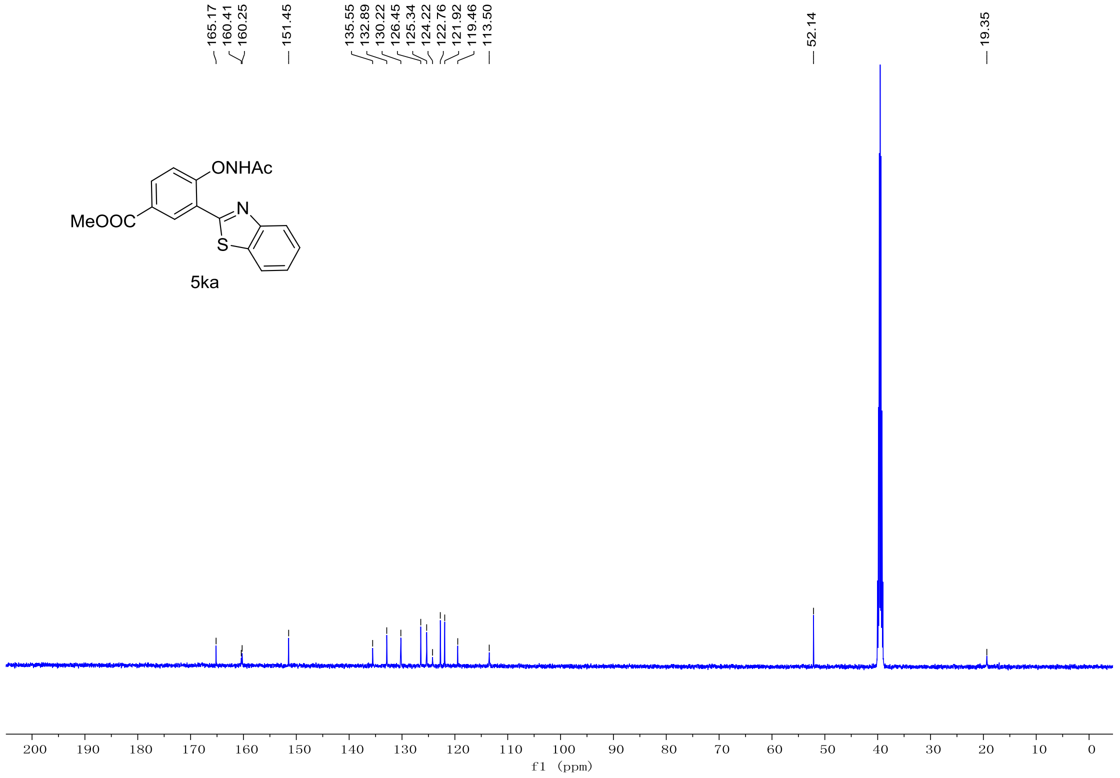
165.17
160.41
160.25
151.45
135.55
132.89
130.22
126.45
125.34
124.22
122.76
121.92
119.46
113.50
52.14
19.35
ONHAc
MeOOC
N
S
5ka
f1 (ppm)

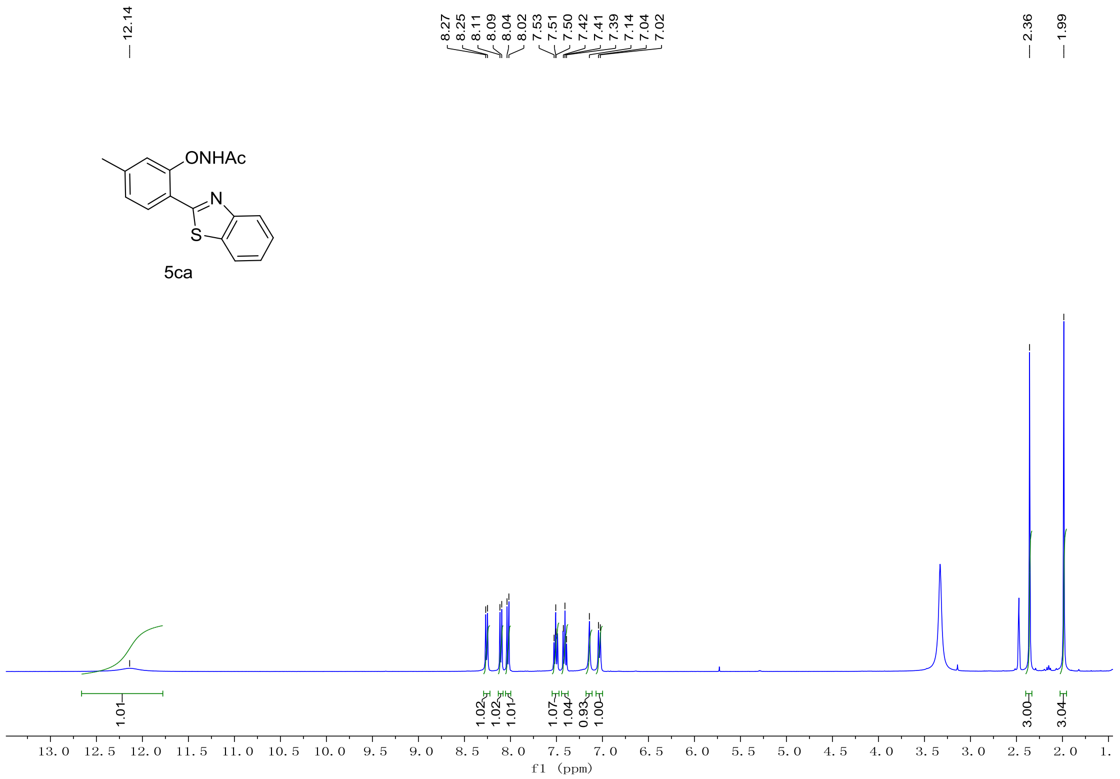

12.14
8.27
8.25
8.11
8.09
8.04
8.02
7.53
7.51
7.50
7.42
7.41
7.39
7.14
7.04
7.02
2.36
1.99
ONHAc
N
S
5ca
1.01
1.02
1.02
1.01
1.07
1.04
0.93
1.00
3.00
3.04
f1 (ppm)

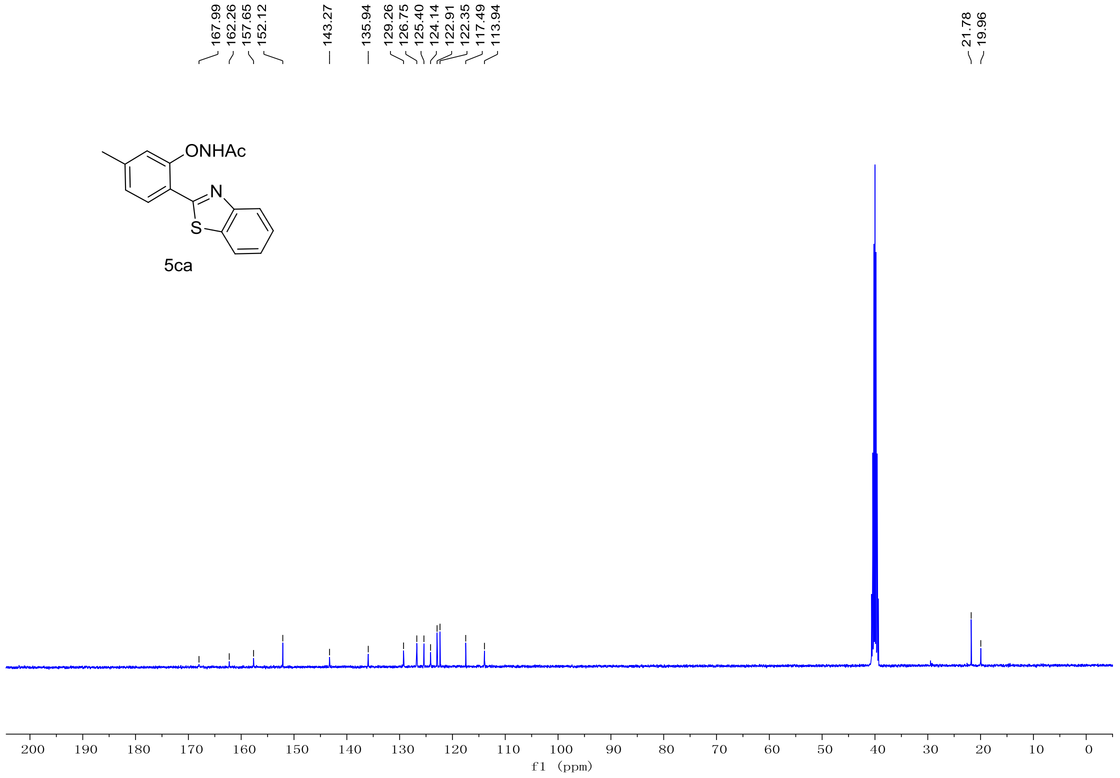

167.99
162.26
157.65
152.12
143.27
135.94
129.26
126.75
125.40
124.14
122.91
122.35
117.49
113.94
21.78
19.96
ONHAc
N
S
5ca
f1 (ppm)

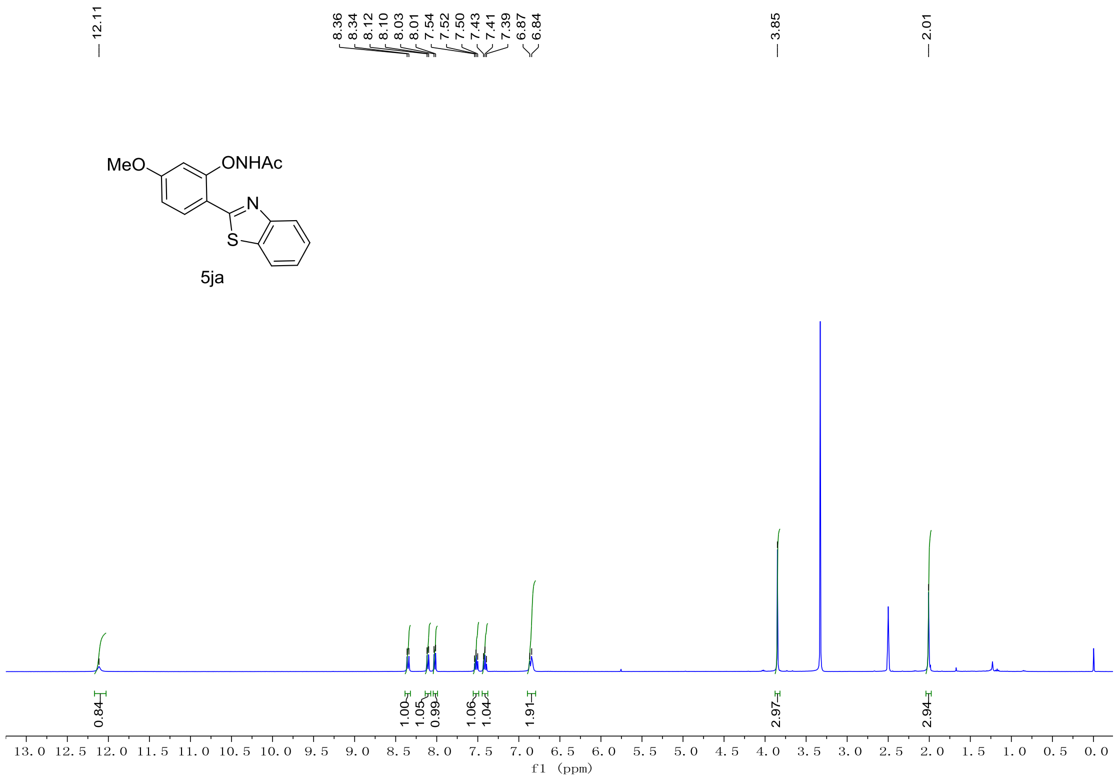
12.11
8.36
8.34
8.12
8.10
8.03
8.01
7.54
7.52
7.50
7.43
7.41
7.39
6.87
6.84
3.85
2.01
MeO
ONHAc
N
S
5ja
0.84
1.00
1.05
0.99
1.06
1.04
1.91
2.97
2.94
13.0 12.5 12.0 11.5 11.0 10.5 10.0 9.5 9.0 8.5 8.0 7.5 7.0 6.5 6.0 5.5 5.0 4.5 4.0 3.5 3.0 2.5 2.0 1.5 1.0 0.5 0.0
f1 (ppm)

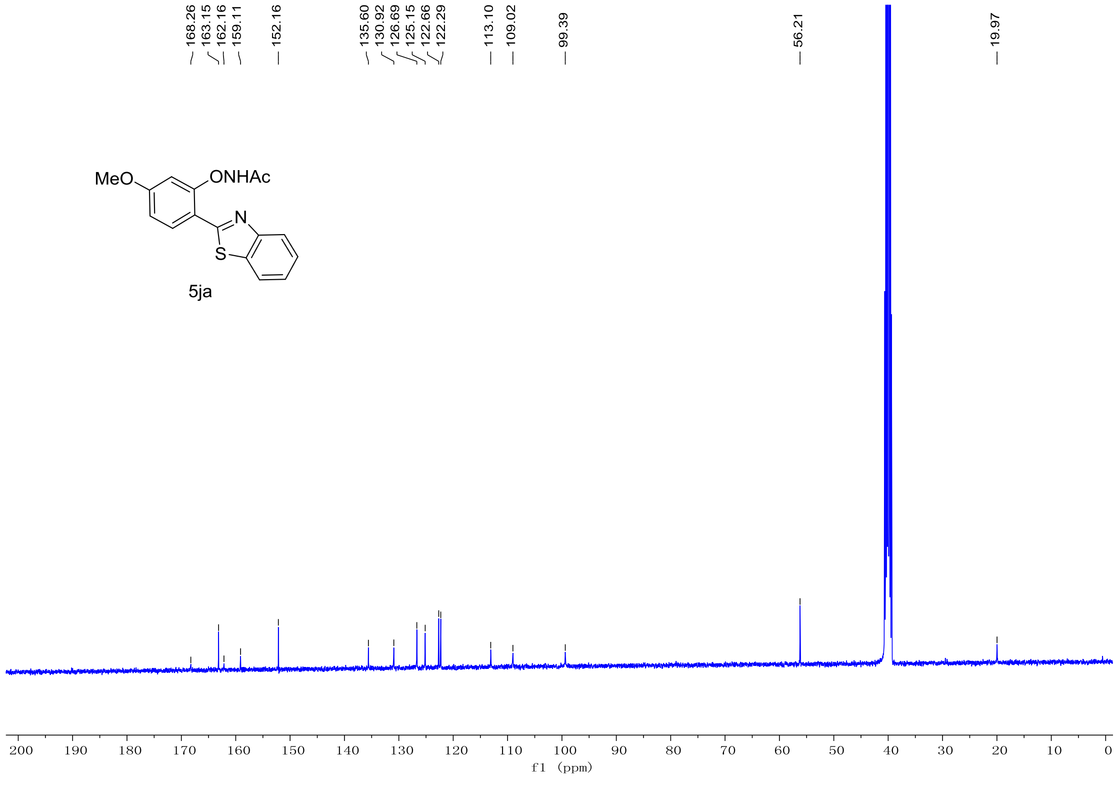
168.26
163.15
162.16
159.11
152.16
135.60
130.92
126.69
125.15
122.66
122.29
113.10
109.02
99.39
56.21
19.97
MeO
ONHAc
N
S
5ja
200 190 180 170 160 150 140 130 120 110 100 90 80 70 60 50 40 30 20 10 0
f1 (ppm)

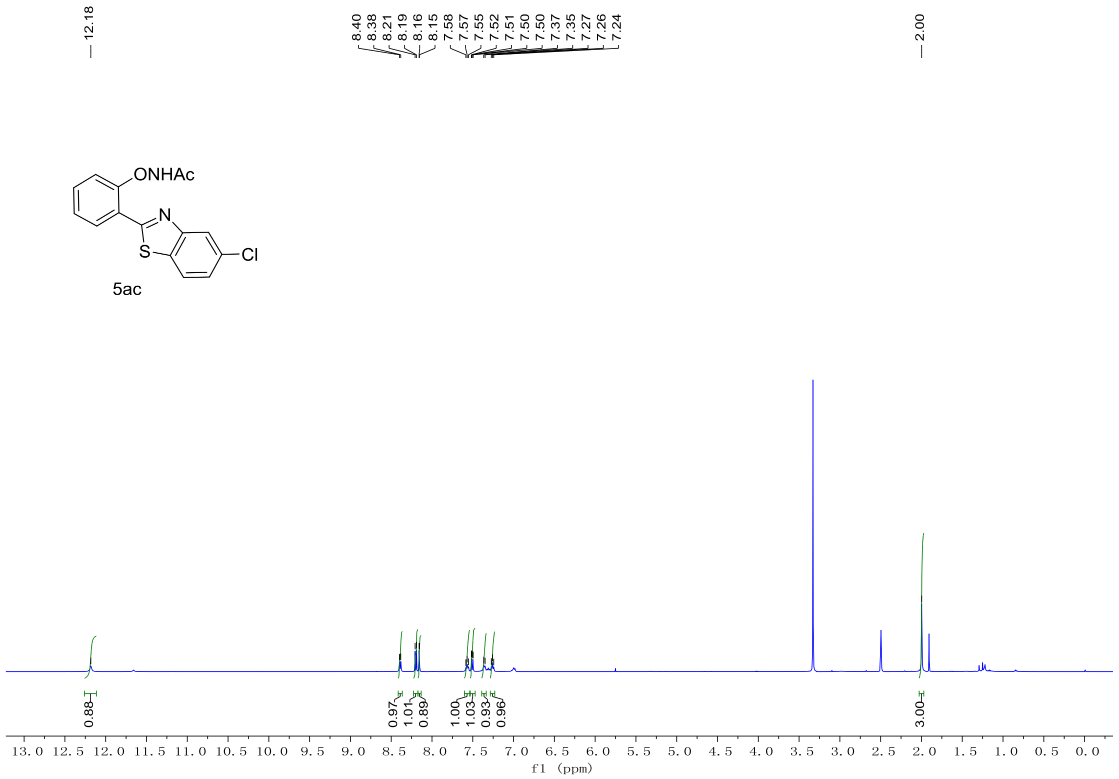
12.18
8.40
8.38
8.21
8.19
8.16
8.15
7.58
7.57
7.55
7.52
7.51
7.50
7.50
7.37
7.35
7.27
7.26
7.24
2.00
ONHAc
N
S
Cl
5ac
0.88
0.97
1.01
0.89
1.00
1.03
0.93
0.96
3.00
13.0 12.5 12.0 11.5 11.0 10.5 10.0 9.5 9.0 8.5 8.0 7.5 7.0 6.5 6.0 5.5 5.0 4.5 4.0 3.5 3.0 2.5 2.0 1.5 1.0 0.5 0.0
f1 (ppm)

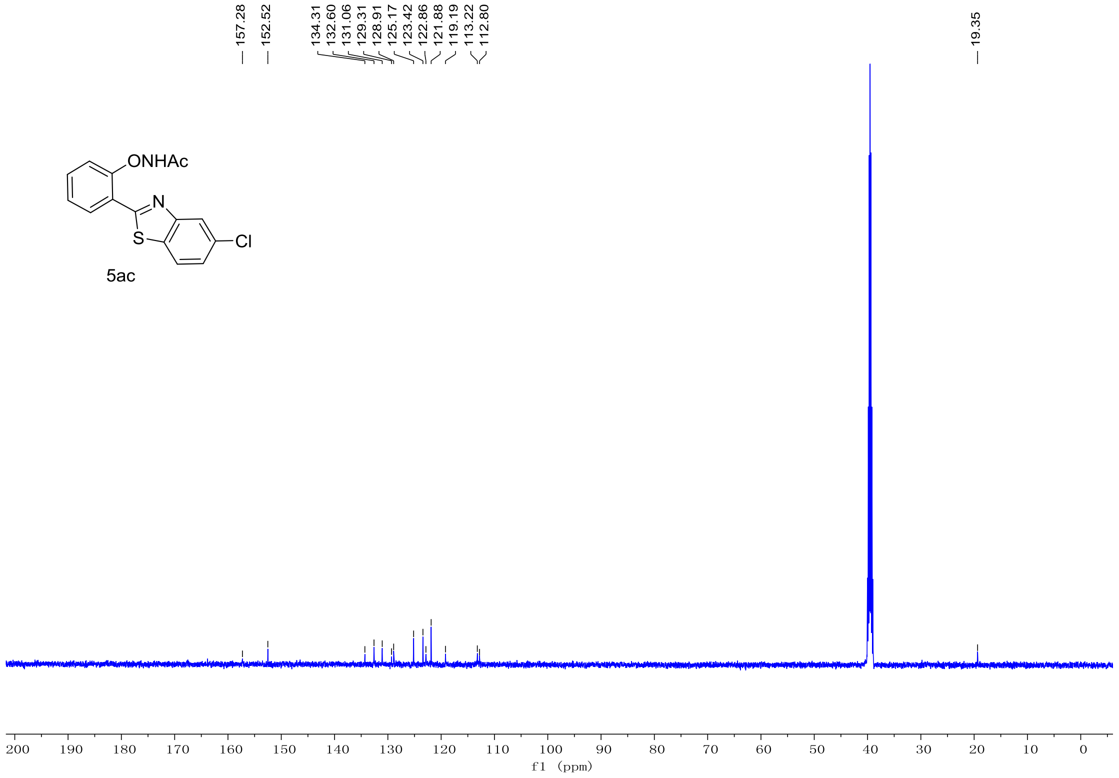
157.28
152.52
134.31
132.60
131.06
129.31
128.91
125.17
123.42
122.86
121.88
119.19
113.22
112.80
19.35
ONHAc
N
S
Cl
5ac
200 190 180 170 160 150 140 130 120 110 100 90 80 70 60 50 40 30 20 10 0
f1 (ppm)

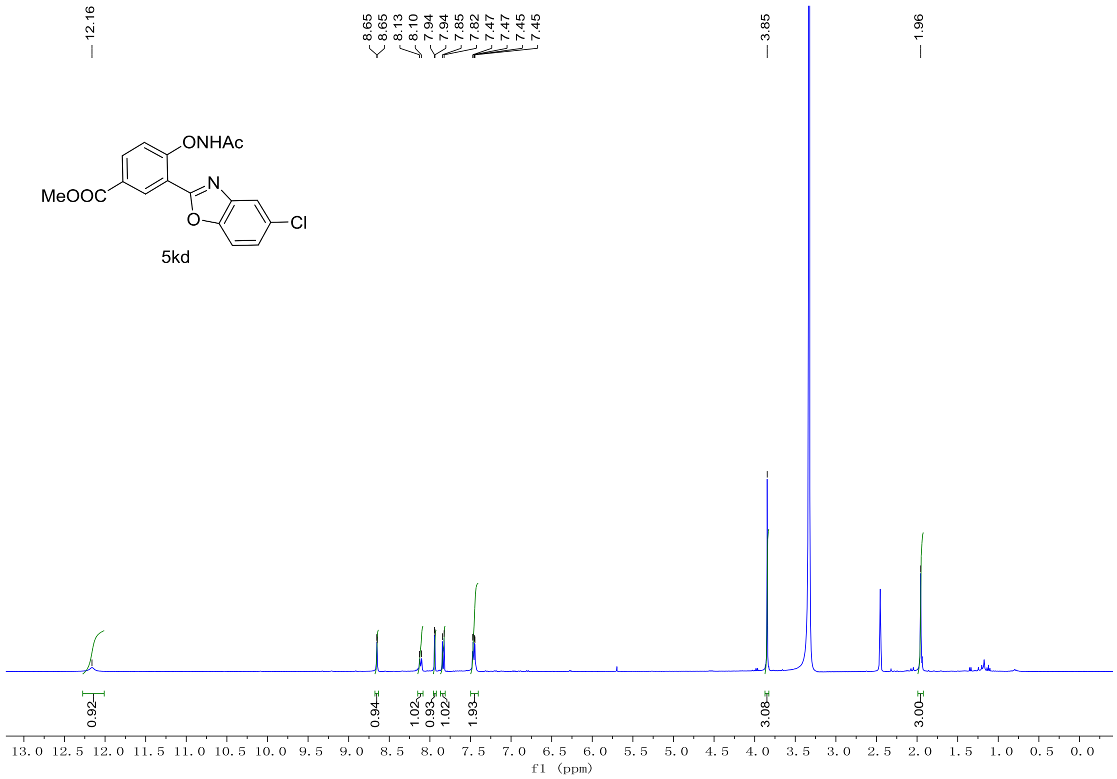
12.16
8.65
8.65
8.13
8.10
7.94
7.94
7.85
7.82
7.47
7.47
7.45
7.45
3.85
1.96
ONHAc
MeOOC
N
O
Cl
5kd
0.92
0.94
1.02
0.93
1.02
1.93
3.08
3.00
13.0 12.5 12.0 11.5 11.0 10.5 10.0 9.5 9.0 8.5 8.0 7.5 7.0 6.5 6.0 5.5 5.0 4.5 4.0 3.5 3.0 2.5 2.0 1.5 1.0 0.5 0.0
f1 (ppm)

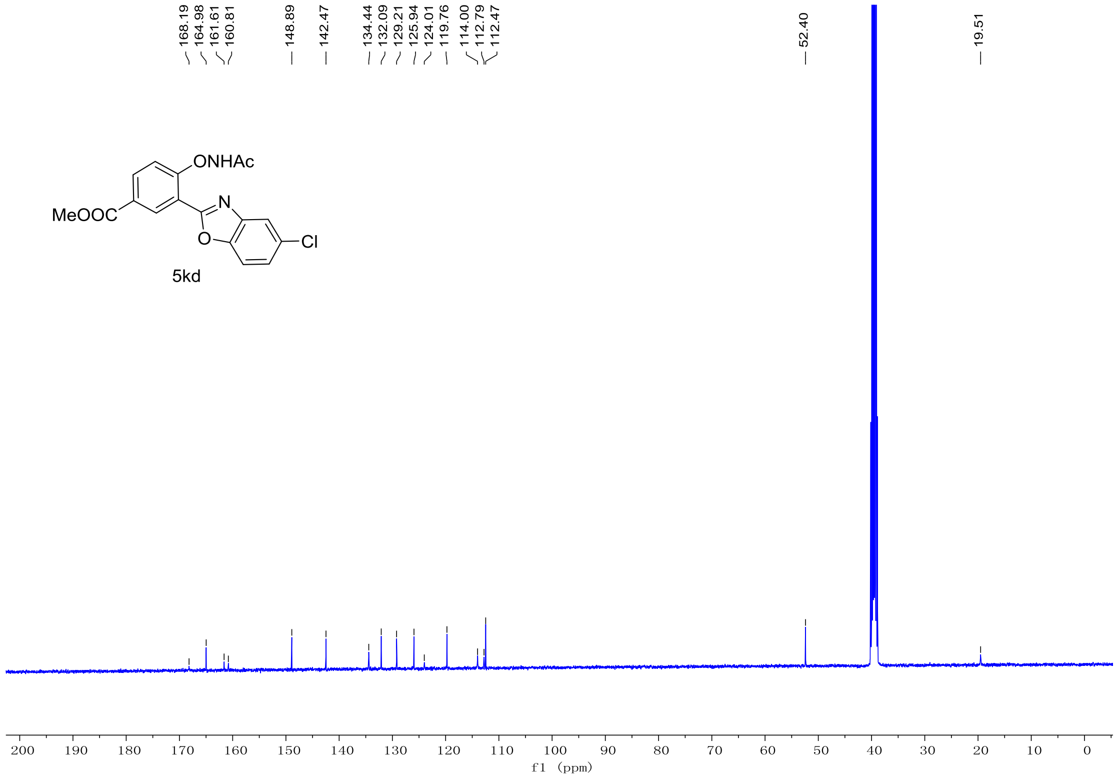
168.19
164.98
161.61
160.81
148.89
142.47
134.44
132.09
129.21
125.94
124.01
119.76
114.00
112.79
112.47
52.40
19.51
ONHAc
MeOOC
N
O
Cl
5kd
200 190 180 170 160 150 140 130 120 110 100 90 80 70 60 50 40 30 20 10 0
f1 (ppm)

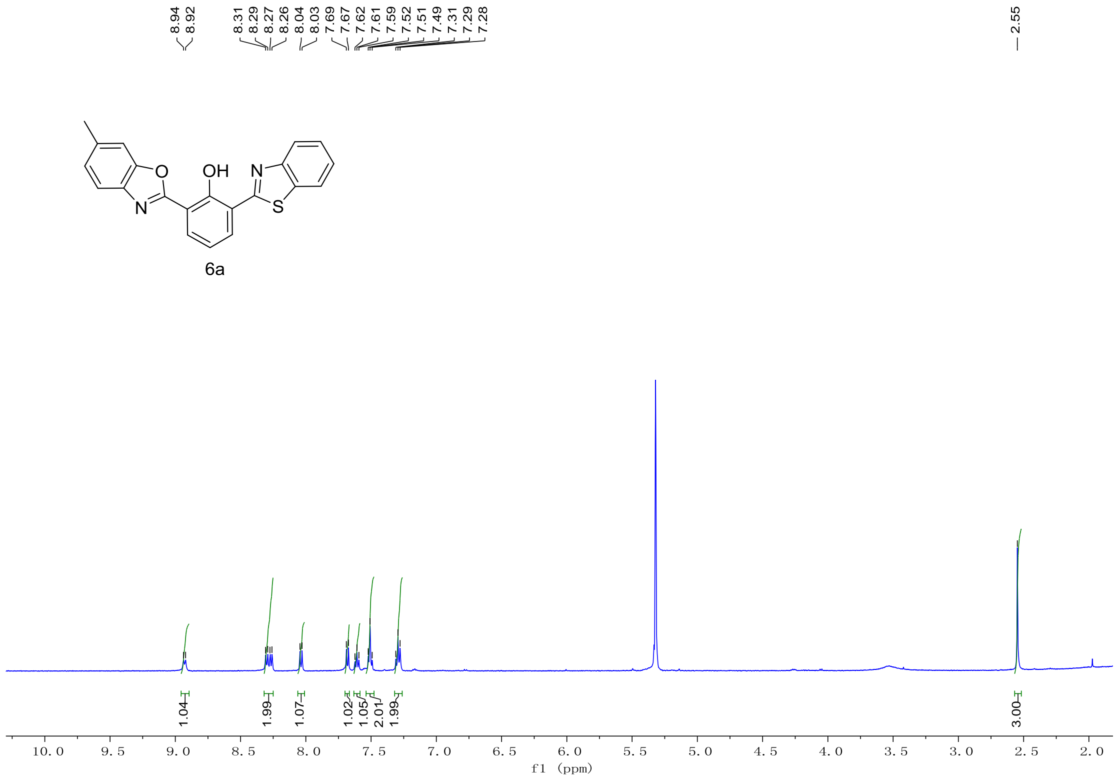
8.94
8.92
8.31
8.29
8.27
8.26
8.04
8.03
7.69
7.67
7.62
7.61
7.59
7.52
7.51
7.49
7.31
7.29
7.28
2.55
OH
N
O
S
6a
1.04
1.99
1.07
1.02
1.05
2.01
1.99
3.00
10.0 9.5 9.0 8.5 8.0 7.5 7.0 6.5 6.0 5.5 5.0 4.5 4.0 3.5 3.0 2.5 2.0
f1 (ppm)

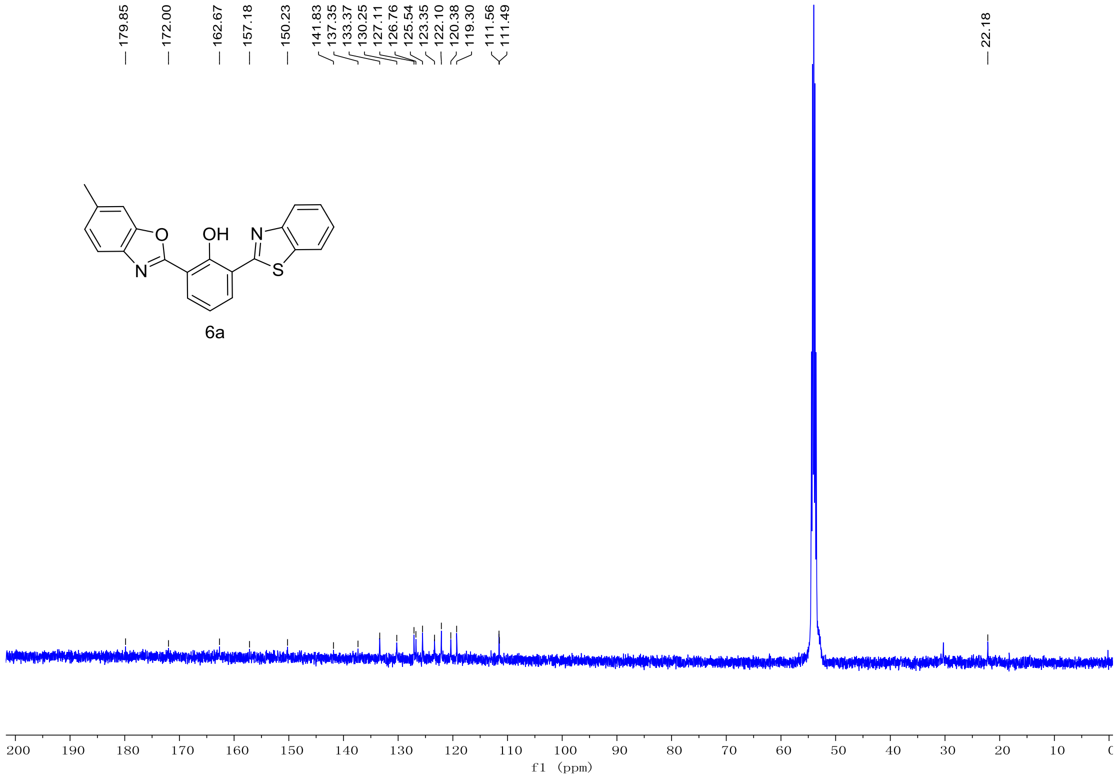
179.85
172.00
162.67
157.18
150.23
141.83
137.35
133.37
130.25
127.11
126.76
125.54
123.35
122.10
120.38
119.30
111.56
111.49
22.18
OH
N
O
S
6a
200 190 180 170 160 150 140 130 120 110 100 90 80 70 60 50 40 30 20 10
f1 (ppm)

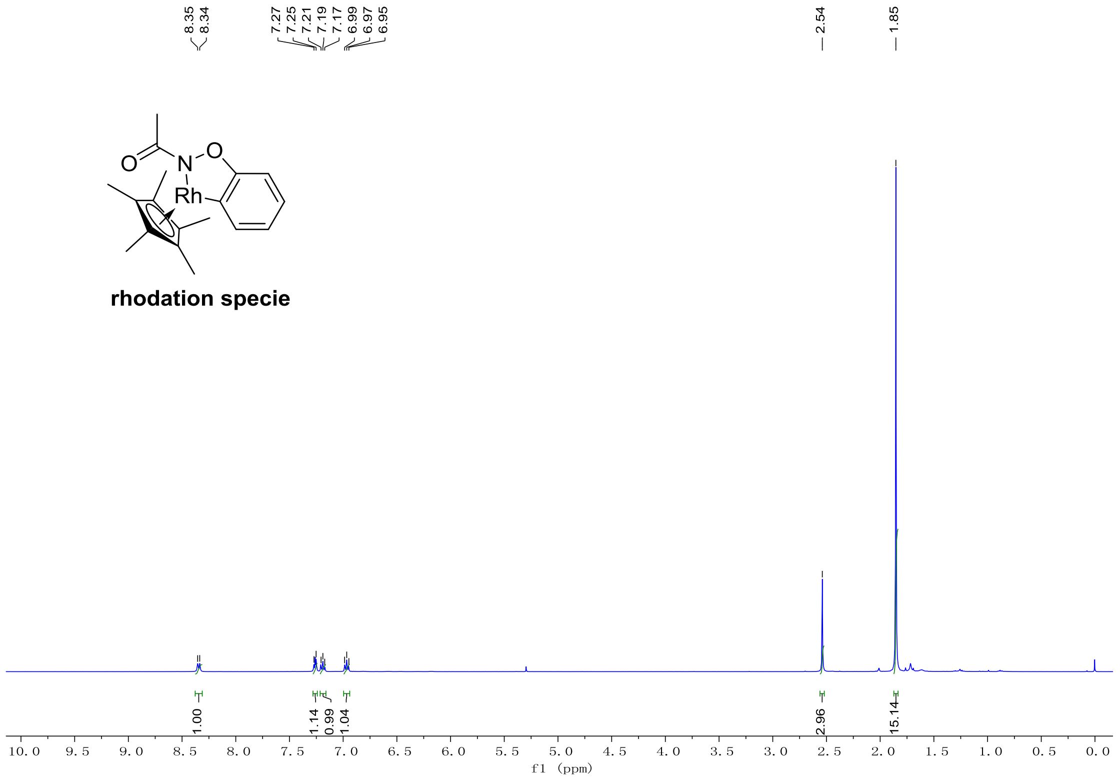
8.35
8.34
7.27
7.25
7.21
7.19
7.17
6.99
6.97
6.95
2.54
1.85
rhodation specie
1.00
1.14
0.99
1.04
2.96
15.14
f1 (ppm)

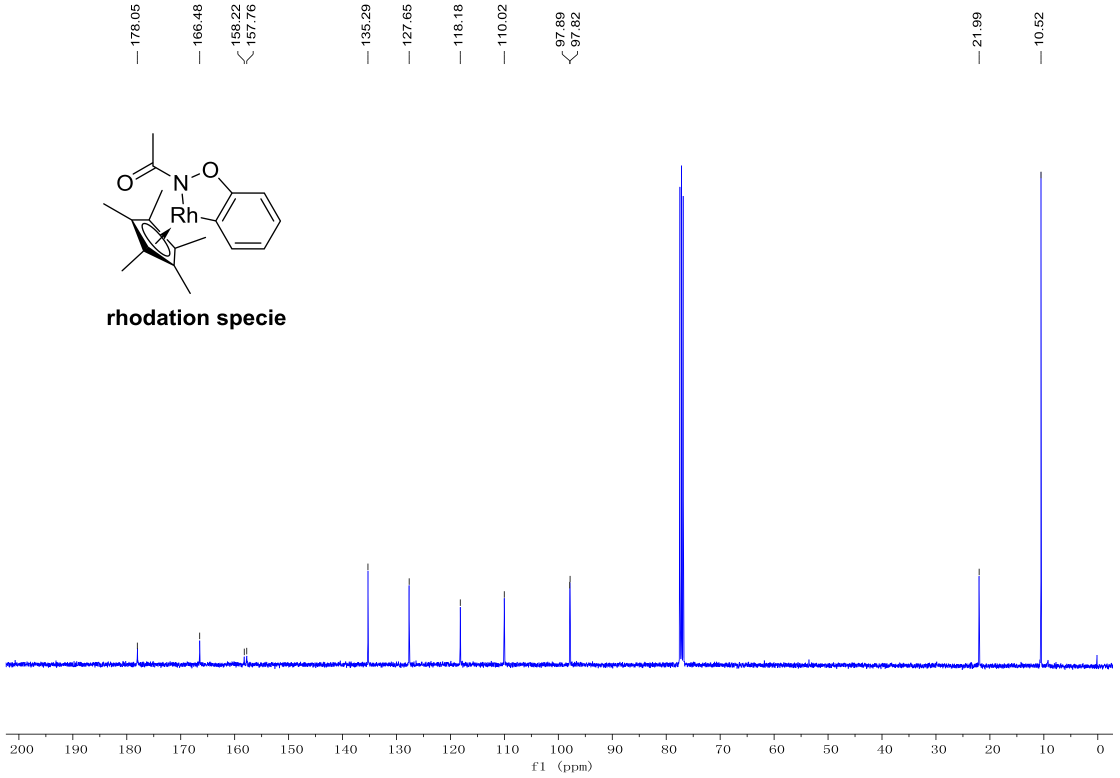
178.05
166.48
158.22
157.76
135.29
127.65
118.18
110.02
97.89
97.82
21.99
10.52
rhodation specie
f1 (ppm)
